# Supplementary material for: Zero-shot learning enables instant denoising and super-resolution in optical fluorescence microscopy
Source: Nat Commun. 2024 May 16;15:4180. doi: 10.1038/s41467-024-48575-9 (PMC11099110; doi:10.1038/s41467-024-48575-9)
Supplement: Supplementary file 1 — Supplementary Information [file 41467_2024_48575_MOESM1_ESM.pdf]

Supplementary Information for

Zero-shot learning enables instant denoising and super-resolution in optical fluorescence microscopy

Chang Qiao*, Yunmin Zeng*, Quan Meng*, Xingye Chen*, Haoyu Chen,
Tao Jiang, Rongfei Wei, Jiabao Guo, Wenfeng Fu, Huaide Lu, Di Li, Yuwang Wang,
Hui Qiao, Jiamin Wu, Dong Li†, Qionghai Dai†

*These authors contributed equally
†Correspondence: lidong@ibp.ac.cn and qhdai@tsinghua.edu.cn

This PDF file includes:

Supplementary Notes 1-4
Supplementary Figures 1-34
Supplementary Tables 1-4
Supplementary References

Contents

Section **Page**

Supplementary Notes..3

1. Theoretical basis of ZS-DeconvNet..3
   a. 2D ZS-DeconvNet..4
   b. 3D ZS-DeconvNet..7
   c. 2D ZS-DeconvNet-SIM 9
   d. 3D ZS-DeconvNet-SIM 14
2. Simulation of dynamic samples and images..16
   a. Simulation of ground truth image of tubular structure.................................16
   b. Simulation of noisy diffraction-limited wide-field images 16
3. Fiji integration for ZS-DeconvNet..18
   a. General consideration...18
   b. Training with ZS-DeconvNet Fiji plugin 18
   c. Inference with ZS-DeconvNet Fiji plugin...19
4. Parameters in the ZS-DeconvNet software..21
   a. Parameters defined in the loss function..21
   b. Recorruption parameters 22
   c. Training parameters..23

Supplementary Figures 25

Supplementary Tables 60

Supplementary Table 1. Implementation details of ZS-DeconvNet 60

Supplementary Table 2. Computation time of ZS-DeconvNet and other deconvolution methods 62

Supplementary Table 3. Imaging conditions of live-cell experiments...................64

Supplementary Table 4. Descriptions and suggested values of hyper-parameters in ZS-DeconvNet 65

Supplementary References...66

Supplementary Notes

1. Theoretical basis of ZS-DeconvNet

The objective function of ZS-DeconvNet is constructed based on the integration of the optical imaging forward model informed inverse problem solver and unsupervised denoising strategies, i.e., the image recorrupting scheme[1] for 2D data and spatially interleaved self-supervised (SiS) denoising scheme[2] for 3D image stacks. For 2D image deconvolution task, the deconvolution loss of ZS-DeconvNet can be formulated as:

$$\mathcal{L}_{dec}(\hat{\mathbf{y}}, \tilde{\mathbf{y}}) = \|f_{\boldsymbol{\theta}}(\hat{\mathbf{y}}) * \mathrm{PSF} - \tilde{\mathbf{y}}\|_2^2 + \lambda \mathcal{R}_{Hessian}\big(f_{\boldsymbol{\theta}}(\hat{\mathbf{y}})\big), \quad (1)$$

where $(\hat{\mathbf{y}}, \tilde{\mathbf{y}})$ indicates the recorrupted image pair, PSF denotes the point spread function (PSF) of the optical system, $f_{\boldsymbol{\theta}}(\hat{\mathbf{y}})$ is the output image of the deconvolution stage, $\mathcal{R}_{Hessian}(\cdot)$ is the Hessian regularization term used to regulating the solution space, and λ is the weighting scalar to balance the impact of the regularization.

And for 3D image stacks, we devised the deconvolution loss of 3D ZS-DeconvNet as follows:

$$\begin{aligned}\mathcal{L}_{dec}(\mathbf{z}) &= \left\|f_{\boldsymbol{\theta}}\big(S_{odd}(\mathbf{z})\big) * \mathrm{PSF} - S_{even}(\mathbf{z})\right\|_2^2 \\ &+\gamma \left\|f_{\boldsymbol{\theta}}\big(S_{odd}(\mathbf{z})\big) * \mathrm{PSF} - S_{even}(\mathbf{z}) - S_{odd}\left(f_{\boldsymbol{\theta}'}(\mathbf{z})\right) + S_{even}\left(f_{\boldsymbol{\theta}'}(\mathbf{z})\right)\right\|_2^2 \\ &+\lambda \mathcal{R}_{Hessian}\left(f_{\boldsymbol{\theta}}\big(S_{odd}(\mathbf{z})\big)\right), \quad (2)\end{aligned}$$

where $\mathbf{z}$ is the 3D noisy image stack, $f_{\boldsymbol{\theta}'}(\mathbf{z})$ is the output image of the denoising stage, $S_{odd}(\cdot)$ and $S_{even}(\cdot)$ represent the axial sampling operators which takes an image stack and returns its odd slices or even slices, respectively, stacked in the same order as the original stack, γ and λ are weighting scalars of the gap amending regularization (GAR) term[2] and the Hessian regularization term.

For implementation of both 2D and 3D ZS-DeconvNet, the ground truths (GT) used for supervising the training procedure are noisy raw images, which are augmented via recorruption or spatially interleaved sampling strategies, thus enabling a totally unsupervised learning scheme. In this note, we mainly showed that the loss function defined on such noisy image pairs for 2D/3D ZS-DeconvNet in Eq. (1) and (2) is statistically equivalent to its supervised counterpart defined on the noisy/clear image pairs from a theoretical perspective. Furthermore, we reasoned that the proposed zero-shot deconvolution scheme can be generalized to multimodal SIM by exploiting the linearity of the classic Wiener-based SIM reconstruction procedure[3], and demonstrated the post-reconstruction multimodal SIM images could be denoised and super-resolved

using ZS-DeconvNet methods in an unsupervised manner.

a. 2D ZS-DeconvNet

In the loss function of ZS-DeconvNet described in Eq. (1), the input and target image pairs $(\hat{\mathbf{y}}, \tilde{\mathbf{y}})$ was generated via a recorruption scheme under the mixed Poisson and Gaussian noise assumption:

$$\hat{\mathbf{y}} = \mathbf{y} + D\mathbf{g}, \tag{3}$$

$$\tilde{\mathbf{y}} = \mathbf{y} - D^{-1}\mathbf{g}, \tag{4}$$

where $D = \alpha I$ is an invertible matrix defined as a magnified unit matrix by a factor of α, which controls the overall magnitude of added noises, and $\mathbf{g}$ is a random noise map ideally following the same noise distribution with the original noisy image $\mathbf{y}$. In optical fluorescence imaging, the digital signal detected by the sensor, e.g., sCMOS camera, typically represents the sum of two variables given by photoelectrons and readout noise, which follows Poisson and Gaussian distributions, respectively[4].

Let's denote the Poisson distribution by $\mathcal{P}(\lambda)$, where λ is the intensity of photoelectrons, and the zero-mean Gaussian noise by $\mathcal{N}(0, \sigma_G^2)$, where σ_G is the standard deviation of the Gaussian noise. Given a typical value of σ_G, e.g., 3.5, which is measured from a blank frame of the sCMOS camera of our imaging system, the mixed Poisson-Gaussian distribution can be well approximated by a single Gaussian distribution $\mathcal{N}(\lambda, \lambda + \sigma_G^2)$. We conducted Pearson correlation between the mixed Poisson-Gaussian distribution and discretized Gaussian distribution across different λ ranging from 1 to 100. Specifically, a discretized Gaussian probability distribution sequence $[G_{X=-x_{gs}}, G_{X=-x_{gs}+1}, \dots, G_{X=x_{gs}}]$ is generated following the distribution $\mathcal{N}(0, \sigma_G^2)$ by listing each probability value for events $\mathrm{X} = -x_{gs}, \mathrm{X} = -x_{gs} + 1, \dots, \mathrm{X} = x_{gs}$, where x_{gs} is the boundary that makes the corresponding accumulated probability achieves 99%. For each λ, a Poisson probability distribution sequence $[P_{X=0}, P_{X=1}, \dots, P_{X=x_{ps}^{\lambda}}]$ is generated in a similar way. Then the discretized mixed Poisson-Gaussian sequence $[M_{X=-x_{mixed}^{\lambda}+\lambda}, M_{X=-x_{mixed}^{\lambda}+\lambda+1}, \dots, M_{X=x_{mixed}^{\lambda}+\lambda}]$ can be calculated using the above Gaussian and Poisson sequence by:

$$M_{X=k} = \sum_{i=0}^{x_{ps}^{\lambda}} P_{X=i}\tilde{G}_{X=k-i}, \mathrm{k} = -x_{mixed}^{\lambda} + \lambda, \dots, x_{mixed}^{\lambda} + \lambda, \tag{5}$$

$$\tilde{G}_{X=p} = \begin{cases} G_{X=p}, p \in \{-x_{gs}, -x_{gs}+1, \dots, x_{gs}\} \\ 0, p \notin \{-x_{gs}, -x_{gs}+1, \dots, x_{gs}\} \end{cases}, \tag{6}$$

where $x_{mixed}^{\lambda} + \lambda$ is the 99% accumulated probability boundary of the Gaussian distribution $\mathcal{N}(\lambda, \lambda + \sigma_G^2)$, which is used to approximate the mixed Poisson-Gaussian distribution. As shown in Supplementary Fig. 32a, in the regions where λ is small, i.e., below 5, the Gaussian noise holds a dominating position in the mixed Poisson-Gaussian noise distribution; while in other regions where λ is larger, the Poisson distribution can be well approximated by a Gaussian distribution, resulting in a high correlation of more than 0.995 between the Gaussian distribution and the mixed distribution regardless of the λ (Supplementary Fig. 32b).

In this regard, the random noise map **g** was sampled from a Gaussian distribution with zero means in our experiments as follows:

$$\mathbf{g} \sim \mathcal{N}(0, \sigma^2 I), \tag{7}$$

$$\sigma^2 = \beta_1 \mathbf{x} + \beta_2, \tag{8}$$

where **x** is the fluorescence intensity from the sample, β_1 is the Poissonian factor affecting the variance of the signal-dependent shot noise, and β_2 is the Gaussian factor representing the variance of additive Gaussian noises, which is dependent to the camera and can be estimated from the sample-free region of the image itself or pre-calibrated following standard protocols[4]. To make the model more robust to the estimation error, β_2 is typically set as a range centering the estimated value $\hat{\beta}_2$, e.g., $[0.8\hat{\beta}_2, 1.2\hat{\beta}_2]$. Ideally, **x** should be the accurate photoelectron count for each pixel and the theoretically optimal value of β_1 is 1. However, we cannot access the clean target in the task of zero-shot deconvolution, so we use the noisy image **y** applied with a low-pass filter as the approximation of **x** in practice. Both the operations of approximating Poisson distribution with Gaussian distribution and applying an inaccurate intensity map introduce extra uncertainty, therefore we typically adopted random value of β_1 within a small range, e.g., [0.5, 1.5], in the image recorruption procedures to improve the robustness of ZS-DeconvNet models.

With the image recorruption scheme described above, the overall noises $\hat{\mathbf{n}}$ and $\tilde{\mathbf{n}}$ of $\hat{\mathbf{y}}$ and $\tilde{\mathbf{y}}$ are independent in that

$$\begin{bmatrix} \hat{\mathbf{n}} \\ \tilde{\mathbf{n}} \end{bmatrix} = \begin{bmatrix} \mathbf{n} + D\mathbf{g} \\ \mathbf{n} - D^{-1}\mathbf{g} \end{bmatrix} \sim \mathcal{N}(0, \Sigma'), \tag{9}$$

where $\mathbf{n} \sim \mathcal{N}(0, \Sigma_x)$ is the original noise of **y**, and Σ' satisfies

$$
\begin{aligned}
\Sigma' &= \begin{bmatrix} I & D \\ I & -D^{-1} \end{bmatrix} \begin{bmatrix} \Sigma_x & 0 \\ 0 & \sigma^2 I \end{bmatrix} \begin{bmatrix} I & I \\ D^T & -(D^{-1})^T \end{bmatrix} \\
&= \begin{bmatrix} \Sigma_x + \sigma^2 D D^T & \Sigma_x - \sigma^2 D (D^{-1})^T \\ \Sigma_x - \sigma^2 D^T D^{-1} & \Sigma_x + \sigma^2 D^{-1} (D^{-1})^T \end{bmatrix} \\
&= \begin{bmatrix} \Sigma_x + \sigma^2 \alpha^2 I & \Sigma_x - \sigma^2 I \\ \Sigma_x - \sigma^2 I & \Sigma_x + \sigma^2 \alpha^{-2} I \end{bmatrix} \\
&= \begin{bmatrix} \Sigma_x + \sigma^2 \alpha^2 I & 0 \\ 0 & \Sigma_x + \sigma^2 \alpha^{-2} I \end{bmatrix}.
\end{aligned} \tag{10}
$$

In the training phase, the overall objective function of ZS-DeconvNet consists of a denoising term and a deconvolution term, which respectively correspond to the denoising stage and the deconvolution stage of the network model:

$$
\mathcal{L}(\hat{\mathbf{y}}, \tilde{\mathbf{y}}) = \mu \mathcal{L}_{den}(\hat{\mathbf{y}}, \tilde{\mathbf{y}}) + (1 - \mu) \mathcal{L}_{dec}(\hat{\mathbf{y}}, \tilde{\mathbf{y}}) \tag{11}
$$

where μ is a scalar weighting factor to balance the two terms, which we empirically set as 0.5 in our experiments. We denote the denoising stage of the ZS-DeconvNet model as $f_{\boldsymbol{\theta}'}$, where $\boldsymbol{\theta}'$ is the trainable parameters of this stage, then the denoising term can be represented as the mean square error (MSE) between the output of the denoising stage and $\tilde{\mathbf{y}}$:

$$
\mathcal{L}_{den}(\hat{\mathbf{y}}, \tilde{\mathbf{y}}) = \left\| f_{\boldsymbol{\theta}'}(\hat{\mathbf{y}}) - \tilde{\mathbf{y}} \right\|_2^2 . \tag{12}
$$

Since the noise of $\hat{\mathbf{y}}$ and $\tilde{\mathbf{y}}$ are independent as illustrated in Eq. (10), the expectation of the denoising loss defined on the noisy image pair $(\hat{\mathbf{y}}, \tilde{\mathbf{y}})$ is statistically equivalent to its counterpart defined on the noisy/clear image pair $(\hat{\mathbf{y}}, \mathbf{x})$ up to a constant:

$$
\begin{aligned}
&\mathbb{E}_{\hat{\mathbf{y}}, \tilde{\mathbf{y}}} \left\{ \left\| f_{\boldsymbol{\theta}'}(\hat{\mathbf{y}}) - \tilde{\mathbf{y}} \right\|_2^2 \right\} \\
&= \mathbb{E}_{\hat{\mathbf{n}}, \tilde{\mathbf{n}}} \left\{ \left\| f_{\boldsymbol{\theta}'}(\mathbf{x} + \hat{\mathbf{n}}) - (\mathbf{x} + \tilde{\mathbf{n}}) \right\|_2^2 \right\} \\
&= \mathbb{E}_{\hat{\mathbf{n}}, \tilde{\mathbf{n}}} \left\{ \left\| f_{\boldsymbol{\theta}'}(\mathbf{x} + \hat{\mathbf{n}}) - \mathbf{x} \right\|_2^2 - 2 \tilde{\mathbf{n}}^{\mathrm{T}} \left(f_{\boldsymbol{\theta}'}(\mathbf{x} + \hat{\mathbf{n}}) - \mathbf{x} \right) + \|\tilde{\mathbf{n}}\|_2^2 \right\} \\
&= \mathbb{E}_{\hat{\mathbf{n}}, \tilde{\mathbf{n}}} \{ \left\| f_{\boldsymbol{\theta}'}(\mathbf{x} + \hat{\mathbf{n}}) - \mathbf{x} \right\|_2^2 \} - \mathbb{E}_{\hat{\mathbf{n}}, \tilde{\mathbf{n}}} \{ 2 \tilde{\mathbf{n}}^{\mathrm{T}} (f_{\boldsymbol{\theta}'}(\mathbf{x} + \hat{\mathbf{n}}) - \mathbf{x}) \} + \mathrm{const} \\
&= \mathbb{E}_{\hat{\mathbf{n}}, \tilde{\mathbf{n}}} \{ \left\| f_{\boldsymbol{\theta}'}(\mathbf{x} + \hat{\mathbf{n}}) - \mathbf{x} \right\|_2^2 \} + \mathrm{const}.
\end{aligned} \tag{13}
$$

Next, we denote by $\mathbf{s}$ the fluorescent sample and H the PSF matrix defined such that the clear diffraction limited image $\mathbf{x}$ writes as the matrix multiplication $\mathbf{x} = H\mathbf{s}$ and the deconvolution loss defined in Eq. (1) rewrites as

$$\mathcal{L}_{dec}(\hat{\mathbf{y}}, \tilde{\mathbf{y}}) = ||Hf_{\boldsymbol{\theta}}(\hat{\mathbf{y}}) - \tilde{\mathbf{y}}||_2^2 + \lambda\mathcal{R}_{Hessian}\big(f_{\boldsymbol{\theta}}(\hat{\mathbf{y}})\big). \tag{14}$$

Of note, $f_{\boldsymbol{\theta}}$ is the entire dual-stage neural network with all trainable parameters $\boldsymbol{\theta}$. Taking the independence between $\hat{\mathbf{n}}$ and $\tilde{\mathbf{n}}$ into consideration, the expectation of the first term, i.e., the degradation term, in Eq. (14) suffices that

$$\begin{aligned}
&\mathbb{E}_{\hat{\mathbf{y}},\tilde{\mathbf{y}}}\{||Hf_{\boldsymbol{\theta}}(\hat{\mathbf{y}}) - \tilde{\mathbf{y}}||_2^2\} \\
&= \mathbb{E}_{\hat{\mathbf{n}},\tilde{\mathbf{n}}}\{||Hf_{\boldsymbol{\theta}}(\mathbf{x}+\hat{\mathbf{n}}) - \mathbf{x}||_2^2 - 2\tilde{\mathbf{n}}^{\mathrm{T}}(Hf_{\boldsymbol{\theta}}(\mathbf{x}+\hat{\mathbf{n}}) - \mathbf{x}) + ||\tilde{\mathbf{n}}||_2^2\} \\
&= \mathbb{E}_{\hat{\mathbf{n}},\tilde{\mathbf{n}}}\{||Hf_{\boldsymbol{\theta}}(\mathbf{x}+\hat{\mathbf{n}}) - \mathbf{x}||_2^2\} - \mathbb{E}_{\hat{\mathbf{n}},\tilde{\mathbf{n}}}\{2\tilde{\mathbf{n}}^{\mathrm{T}}(Hf_{\boldsymbol{\theta}}(\mathbf{x}+\hat{\mathbf{n}}) - \mathbf{x})\} + \mathrm{const} \\
&= \mathbb{E}_{\hat{\mathbf{n}},\tilde{\mathbf{n}}}\{||Hf_{\boldsymbol{\theta}}(\mathbf{x}+\hat{\mathbf{n}}) - H\mathbf{s}||_2^2\} + \mathrm{const}.
\end{aligned} \tag{15}$$

Because the PSF matrix H is a linear operator, the minimization of Eq. (15) defined on recorrupted image pairs $(\hat{\mathbf{y}}, \tilde{\mathbf{y}})$ will optimally converges to the solution that satisfies $f_{\boldsymbol{\theta}}(\mathbf{x}+\hat{\mathbf{n}}) = \mathbf{s}$, which indicates that with abundant recorrupted image pairs, a well-trained ZS-DeconvNet model $f_{\boldsymbol{\theta}}$ is able to recover the clear super-resolved image of the fluorescent sample $\mathbf{s}$ directly from its diffraction-limited noisy counterpart $\hat{\mathbf{y}}$. Moreover, in that the gain of the normalized PSF matrix H is 1, the overall intensity of the network output $f_{\boldsymbol{\theta}}(\mathbf{x}+\hat{\mathbf{n}})$ is the same as the input image without noises, i.e., $\mathbf{x}$, which underlies the intensity conservation characteristic of ZS-DeconvNet (Supplementary Fig. 7e-g).

In practical implementations, we typically up-sampled the super-resolved image in ZS-DeconvNet by a factor of 2 so as to permit higher output resolution which could be limited by the Nyquist sampling criterion otherwise, and then down-sampled it back to the original size after convolving PSF in loss calculation. Moreover, the second term in Eq. (14), i.e., the Hessian continuity regularization term, was applied to regulate the training process of ZS-DeconvNet, empirically yielding a more stable convergence in training and better deconvolution performance.

b. 3D ZS-DeconvNet

The objective function of 3D ZS-DeconvNet is constructed based on the 3D imaging model informed inverse problem solver equipped with our previously proposed spatially interleaved sampling strategy[2], which makes use of the spatial continuity and noise independence between z-slices of a single image stack to realize the unsupervised denoising. Similar to 2D cases, the overall objective function consists of a denoising term and a deconvolution term for supervising the denoising stage and the deconvolution stage of 3D ZS-DeconvNet, respectively:

$$\mathcal{L}(\mathbf{z}) = \mu\mathcal{L}_{den}(\mathbf{z}) + (1-\mu)\mathcal{L}_{dec}(\mathbf{z}), \tag{16}$$

where $\mathbf{z}$ is the 3D noisy image stack and $\mathcal{L}_{den}(\mathbf{z})$ is composed of a self-supervised term and a gap-amending regularization (GAR) term[2]:

$$\mathcal{L}_{den}(\mathbf{z}) = \left\| f_{\boldsymbol{\theta}'}\left(S_{odd}(\mathbf{z})\right) - S_{even}(\mathbf{z}) \right\|_2^2$$
$$+\gamma \left\| f_{\boldsymbol{\theta}'}\left(S_{odd}(\mathbf{z})\right) - S_{even}(\mathbf{z}) - \left(S_{odd}\left(f_{\boldsymbol{\theta}'}(\mathbf{z})\right) - S_{even}\left(f_{\boldsymbol{\theta}'}(\mathbf{z})\right)\right) \right\|_2^2, \tag{17}$$

where $S_{odd}(\cdot)$ and $S_{even}(\cdot)$ represent the axial sampling operators which takes an image stack and returns its odd slices or even slices, respectively, stacked in the same order as the original stack, and γ is the weight of the GAR term. The GAR term is proven to be able to amend the non-zero gap between expectations $E_{\mathbf{z}}\{S_{odd}(\mathbf{z})\}$ and $E_{\mathbf{z}}\{S_{even}(\mathbf{z})\}$, thus relieving the over-smoothing and axial drifting of the outputs[2]. For simplification, we hereinafter denote $\hat{\mathbf{z}} = S_{odd}(\mathbf{z})$ and $\tilde{\mathbf{z}} = S_{even}(\mathbf{z})$. Let $\hat{\mathbf{n}}$ and $\tilde{\mathbf{n}}$ be the independent noise of $\hat{\mathbf{z}}$ and $\tilde{\mathbf{z}}$ with a Gaussian distribution of $\mathcal{N}(0, \sigma^2)$, $\hat{\mathbf{x}}$, $\tilde{\mathbf{x}}$, and $\mathbf{x}$ be the odd slices, even slices, and all slices of the corresponding clear image stack with $\boldsymbol{\varepsilon} = \hat{\mathbf{x}} - \tilde{\mathbf{x}} = E_{\mathbf{z}}\{\hat{\mathbf{x}} - \tilde{\mathbf{z}}\}$. Then it holds that

$$\begin{aligned}
&\mathbb{E}_{\mathbf{z}}\left\{\left\| f_{\boldsymbol{\theta}'}(\hat{\mathbf{z}}) - \tilde{\mathbf{z}} \right\|_2^2\right\} \\
&= \mathbb{E}_{\mathbf{z}}\{\left\| f_{\boldsymbol{\theta}'}(\hat{\mathbf{z}}) - \hat{\mathbf{x}} + \hat{\mathbf{x}} - \tilde{\mathbf{z}} \right\|_2^2\} \\
&= \mathbb{E}_{\mathbf{z}}\left\{\left\| f_{\boldsymbol{\theta}'}(\hat{\mathbf{z}}) - \hat{\mathbf{x}} \right\|_2^2\} + \mathbb{E}_{\mathbf{z}}\{2\left(f_{\boldsymbol{\theta}'}(\hat{\mathbf{z}}) - \hat{\mathbf{x}}\right)^{\mathrm{T}}(\hat{\mathbf{x}} - \tilde{\mathbf{z}})\} + \mathbb{E}_{\mathbf{z}}\{\|\hat{\mathbf{x}} - \tilde{\mathbf{z}}\|_2^2\right\} \\
&= \mathbb{E}_{\mathbf{z}}\{\left\| f_{\boldsymbol{\theta}'}(\hat{\mathbf{z}}) - \hat{\mathbf{x}} \right\|_2^2\} + \mathbb{E}_{\mathbf{z}}\left\{2\left(f_{\boldsymbol{\theta}'}(\hat{\mathbf{z}}) - \hat{\mathbf{x}}\right)^{\mathrm{T}}(\hat{\mathbf{x}} - \tilde{\mathbf{z}})\right\} + \mathbb{E}_{\mathbf{z}}\{\boldsymbol{\varepsilon}^{\mathrm{T}}\boldsymbol{\varepsilon}\} + \sigma^2 \\
&= \mathbb{E}_{\mathbf{z}}\left\{\left\| f_{\boldsymbol{\theta}'}(\hat{\mathbf{z}}) - \hat{\mathbf{x}} \right\|_2^2\right\} + 2\mathbb{E}_{\mathbf{z}}\{(f_{\boldsymbol{\theta}'}(\hat{\mathbf{z}}) - \hat{\mathbf{x}})^{\mathrm{T}}\}\mathbb{E}_{\mathbf{z}}\{\hat{\mathbf{x}} - \tilde{\mathbf{z}}\} + \mathbb{E}_{\mathbf{z}}\{\boldsymbol{\varepsilon}^{\mathrm{T}}\boldsymbol{\varepsilon}\} + \sigma^2 \\
&= \mathbb{E}_{\mathbf{z}}\left\{\left\| f_{\boldsymbol{\theta}'}(\hat{\mathbf{z}}) - \hat{\mathbf{x}} \right\|_2^2\right\} + 2\boldsymbol{\varepsilon}^{\mathrm{T}}\mathbb{E}_{\mathbf{z}}\{f_{\boldsymbol{\theta}'}(\hat{\mathbf{z}}) - \hat{\mathbf{x}}\} + \mathbb{E}_{\mathbf{z}}\{\boldsymbol{\varepsilon}^{\mathrm{T}}\boldsymbol{\varepsilon}\} + \sigma^2 \\
&\to \mathbb{E}_{\mathbf{z}}\{\| f_{\boldsymbol{\theta}'}(\hat{\mathbf{z}}) - \hat{\mathbf{x}} \|_2^2\} + \sigma^2 (\boldsymbol{\varepsilon} \to \mathbf{0}).
\end{aligned} \tag{18}$$

Eq. (18) shows that the expectation of the denoising loss calculated between two interleaved noisy stacks equals to its counterpart calculated between paired noisy/clear image stacks on condition of a sufficiently small $\boldsymbol{\varepsilon}$. This holds true in most volumetric imaging experiments in that the axial sampling satisfies the Nyquist sampling theorem, i.e., the stepping distance in z-axis is below one half of the axial resolution, that is, the adjacent z-slices approximately contain the same biological context in most areas.

Similar to the 2D case, we denote by $\widehat{H}$ the matrix form of axially down-sampled PSF. Taking other abbreviated symbols, the deconvolution loss of 3D ZS-DeconvNet described in Eq. (2) can be rewrote as:

$$\mathcal{L}_{dec}(\mathbf{z}) = \left\|\hat{H} f_{\boldsymbol{\theta}}(\hat{\mathbf{z}}) - \tilde{\mathbf{z}}\right\|_2^2$$
$$+\gamma\left\|\hat{H} f_{\boldsymbol{\theta}}(\hat{\mathbf{z}}) - \tilde{\mathbf{z}} - S_{odd}\left(f_{\boldsymbol{\theta}'}(\mathbf{z})\right) + S_{even}\left(f_{\boldsymbol{\theta}'}(\mathbf{z})\right)\right\|_2^2 + \lambda\mathcal{R}_{Hessian}\left(f_{\boldsymbol{\theta}}(\hat{\mathbf{z}})\right). \quad (19)$$

If we denote $\hat{\mathbf{s}}$ the volumetric fluorescent sample related to $\hat{\mathbf{z}}$, the expectation of the first term of Eq. (19), i.e., the degradation term, which is primarily responsible for denoising and deconvolution, holds that

$$\begin{aligned}
&\mathbb{E}_{\mathbf{z}}\left\{\left\|\hat{H} f_{\boldsymbol{\theta}}(\hat{\mathbf{z}}) - \tilde{\mathbf{z}}\right\|_2^2\right\} \\
&= \mathbb{E}_{\mathbf{z}}\{\left\|\hat{H} f_{\boldsymbol{\theta}}(\hat{\mathbf{z}}) - \hat{\mathbf{x}} + \hat{\mathbf{x}} - \tilde{\mathbf{z}}\right\|_2^2\} \\
&= \mathbb{E}_{\mathbf{z}}\left\{\left\|\hat{H} f_{\boldsymbol{\theta}}(\hat{\mathbf{z}}) - \hat{\mathbf{x}}\right\|_2^2\right\} + \mathbb{E}_{\mathbf{z}}\{2\left(\hat{H} f_{\boldsymbol{\theta}}(\hat{\mathbf{z}}) - \hat{\mathbf{x}}\right)^{\mathrm{T}}(\hat{\mathbf{x}} - \tilde{\mathbf{z}})\} + \mathbb{E}_{\mathbf{z}}\left\{\|\hat{\mathbf{x}} - \tilde{\mathbf{z}}\|_2^2\right\} \\
&= \mathbb{E}_{\mathbf{z}}\{\left\|\hat{H} f_{\boldsymbol{\theta}}(\hat{\mathbf{z}}) - \hat{\mathbf{x}}\right\|_2^2\} + \mathbb{E}_{\mathbf{z}}\left\{2\left(\hat{H} f_{\boldsymbol{\theta}}(\hat{\mathbf{z}}) - \hat{\mathbf{x}}\right)^{\mathrm{T}}(\hat{\mathbf{x}} - \tilde{\mathbf{z}})\right\} + \mathbb{E}_{\mathbf{z}}\{\boldsymbol{\varepsilon}^{\mathrm{T}}\boldsymbol{\varepsilon}\} + \sigma^2 \\
&= \mathbb{E}_{\mathbf{z}}\left\{\left\|\hat{H} f_{\boldsymbol{\theta}}(\hat{\mathbf{z}}) - \hat{\mathbf{x}}\right\|_2^2\right\} + 2\mathbb{E}_{\mathbf{z}}\{(\hat{H} f_{\boldsymbol{\theta}}(\hat{\mathbf{z}}) - \hat{\mathbf{x}})^{\mathrm{T}}\}\mathbb{E}_{\mathbf{z}}\{\hat{\mathbf{x}} - \tilde{\mathbf{z}}\} + \mathbb{E}_{\mathbf{z}}\{\boldsymbol{\varepsilon}^{\mathrm{T}}\boldsymbol{\varepsilon}\} + \sigma^2 \\
&= \mathbb{E}_{\mathbf{z}}\left\{\left\|\hat{H} f_{\boldsymbol{\theta}}(\hat{\mathbf{z}}) - \hat{H}\hat{\mathbf{s}}\right\|_2^2\right\} + 2\boldsymbol{\varepsilon}^{\mathrm{T}}\mathbb{E}_{\mathbf{z}}\left\{\hat{H} f_{\boldsymbol{\theta}}(\hat{\mathbf{z}}) - \hat{\mathbf{x}}\right\} + \mathbb{E}_{\mathbf{z}}\{\boldsymbol{\varepsilon}^{\mathrm{T}}\boldsymbol{\varepsilon}\} + \sigma^2 \\
&= \mathbb{E}_{\mathbf{z}}\left\{\left\|\hat{H}(f_{\boldsymbol{\theta}}(\hat{\mathbf{z}}) - \hat{\mathbf{s}})\right\|_2^2\right\} + 2\boldsymbol{\varepsilon}^{\mathrm{T}}\mathbb{E}_{\mathbf{z}}\left\{\hat{H} f_{\boldsymbol{\theta}}(\hat{\mathbf{z}}) - \hat{\mathbf{x}}\right\} + \mathbb{E}_{\mathbf{z}}\{\boldsymbol{\varepsilon}^{\mathrm{T}}\boldsymbol{\varepsilon}\} + \sigma^2 \\
&\to \mathbb{E}_{\mathbf{z}}\left\{\left\|\hat{H}(f_{\boldsymbol{\theta}}(\hat{\mathbf{z}}) - \hat{\mathbf{s}})\right\|_2^2\right\} + \sigma^2(\boldsymbol{\varepsilon} \to \mathbf{0}). \quad (20)
\end{aligned}$$

Eq. (20) shows that when the gap ε diminishes, the expectation of our unsupervised deconvolution term is equivalent with the supervised one up to a constant σ^2. Ideally, minimization of Eq. (20) will lead to an optimal solution $f_{\boldsymbol{\theta}^*}$ subject to $f_{\boldsymbol{\theta}^*}(\hat{\mathbf{z}}) = \hat{\mathbf{s}}$.

The gap amending regularizations applied in Eqs. (17) and (19) are aimed to further mitigate non-zero gap induced deviation in training in that they hold a zero expectation with an optimal network model $f_{\boldsymbol{\theta}^*}$:

$$\begin{aligned}
&\mathbb{E}_{\mathbf{z}}\left\{f_{\boldsymbol{\theta}'^*}(\hat{\mathbf{z}}) - \tilde{\mathbf{z}} - \left(S_{odd}\left(f_{\boldsymbol{\theta}'^*}(\mathbf{z})\right) - S_{even}\left(f_{\boldsymbol{\theta}'^*}(\mathbf{z})\right)\right)\right\} \\
&= \mathbb{E}_{\mathbf{z}}\{\hat{\mathbf{x}} - \tilde{\mathbf{z}} - (\hat{\mathbf{x}} - \tilde{\mathbf{x}})\} \\
&= \tilde{\mathbf{x}} - \mathbb{E}_{\mathbf{z}}\{\tilde{\mathbf{z}}\} = \mathbf{0}, \quad (21)
\end{aligned}$$

$$\begin{aligned}
&\mathbb{E}_{\mathbf{z}}\left\{\hat{H} f_{\boldsymbol{\theta}^*}(\hat{\mathbf{z}}) - \tilde{\mathbf{z}} - \left(S_{odd}\left(f_{\boldsymbol{\theta}'^*}(\mathbf{z})\right) - S_{even}\left(f_{\boldsymbol{\theta}'^*}(\mathbf{z})\right)\right)\right\} \\
&= \mathbb{E}_{\mathbf{z}}\left\{\hat{H}\hat{\mathbf{s}} - \tilde{\mathbf{z}} - \left(S_{odd}\left(f_{\boldsymbol{\theta}'^*}(\mathbf{z})\right) - S_{even}\left(f_{\boldsymbol{\theta}'^*}(\mathbf{z})\right)\right)\right\} \\
&= \mathbb{E}_{\mathbf{z}}\{\hat{\mathbf{x}} - \tilde{\mathbf{z}} - (\hat{\mathbf{x}} - \tilde{\mathbf{x}})\} \\
&= \tilde{\mathbf{x}} - \mathbb{E}_{\mathbf{z}}\{\tilde{\mathbf{z}}\} = \mathbf{0}, \quad (22)
\end{aligned}$$

where $f_{\boldsymbol{\theta}'^*}$ denotes the optimal parameters of the denoising stage which suffices $S_{odd}\left(f_{\boldsymbol{\theta}'^*}(\mathbf{z})\right) = S_{odd}(\mathbf{x}) = \hat{\mathbf{x}}$ and $S_{even}\left(f_{\boldsymbol{\theta}'^*}(\mathbf{z})\right) = S_{even}(\mathbf{x}) = \tilde{\mathbf{x}}$.

c. 2D ZS-DeconvNet-SIM

Structured illumination microscopy (SIM) is usually recognized as a balanced super-resolution option in terms of resolution, speed, and duration, which makes it particularly suitable for live-sample imaging[5]. Typically, 2D SIM such as total internal reflective fluorescence (TIRF) SIM and grazing incidence (GI) SIM provides a sub-100 nm resolution by acquiring nine raw SIM images modulated with varying illumination patterns for the same scene and then reconstruction them into the super-resolved image via an analytical algorithm. However, conventional SIM reconstruction methods are prone to generating artifacts especially with input images of low signal-to-noise ratio (SNR)[2, 6]. To statistically model the noise-induced artifacts in post-reconstruction SIM images, we first revisit the conventional SIM reconstruction algorithm[3] with photon noise taken into consideration.

In the forward model of SIM imaging, the fluorescent specimen is illuminated by a spatially variant illumination pattern, and the observed image is the convolution between the emission fluorescence and the system point spread function (PSF) with additive photon noises:

$$\mathbf{I}_{d,m}(\mathbf{r}) = \left[\mathbf{S}(\mathbf{r})\left(1 + c_{d,m}\cos\left(2\pi\mathbf{p}_d \cdot \mathbf{r} + \varphi_{d,m}\right)\right)\right] \otimes P(\mathbf{r}) + \mathbf{N}_{d,m}(\mathbf{r}), \quad (23)$$

where $\mathbf{S}(\mathbf{r})$ and $P(\mathbf{r})$ denote the fluorescent sample and the system's PSF; $\mathbf{I}_{d,m}(\mathbf{r})$, $\mathbf{N}_{d,m}(\mathbf{r})$, $\mathbf{p}_d$, $c_{d,m}$, and $\varphi_{d,m}$ represent the acquired raw SIM image, additive photon noise, wave vector, modulation depth, and illumination pattern phases, respectively, corresponding to pattern orientations $d = 1,2,3$ and phases $m = -1,0,1$; $\cdot$ and $\otimes$ denote Hadamard product and convolution. If we denote by $O(\mathbf{k})$ the optical transfer function (OTF) of the system, the Fourier transform of Eq. (23) will take the form

$$\mathbf{I}_{d,m}(\mathbf{k}) = \left[\mathbf{S}(\mathbf{k}) + \frac{c_{d,m}}{2} e^{i\varphi_{d,m}} \mathbf{S}(\mathbf{k} - \mathbf{p}_d) + \frac{c_{d,m}}{2} e^{-i\varphi_{d,m}} \mathbf{S}(\mathbf{k} + \mathbf{p}_d)\right] O(\mathbf{k}) + \mathbf{N}_{d,m}(\mathbf{k}). \quad (24)$$

For each pattern orientation d, raw SIM images of 3 different phases labelled by $m = -1,0,1$ are acquired, resulting in a ternary homogeneous linear equation represented in matrix form as

$$\begin{bmatrix} \mathbf{I}_{d,0}(\mathbf{k}) \\ \mathbf{I}_{d,1}(\mathbf{k}) \\ \mathbf{I}_{d,-1}(\mathbf{k}) \end{bmatrix} = \begin{bmatrix} 1 & \frac{c_{d,0}}{2} e^{i\varphi_{d,0}} & \frac{c_{d,0}}{2} e^{-i\varphi_{d,0}} \\ 1 & \frac{c_{d,1}}{2} e^{i\varphi_{d,1}} & \frac{c_{d,1}}{2} e^{-i\varphi_{d,1}} \\ 1 & \frac{c_{d,-1}}{2} e^{i\varphi_{d,-1}} & \frac{c_{d,-1}}{2} e^{-i\varphi_{d,-1}} \end{bmatrix} \begin{bmatrix} \mathbf{S}(\mathbf{k})O(\mathbf{k}) \\ \mathbf{S}(\mathbf{k} - \mathbf{p}_\mathrm{d})O(\mathbf{k}) \\ \mathbf{S}(\mathbf{k} + \mathbf{p}_\mathrm{d})O(\mathbf{k}) \end{bmatrix} + \begin{bmatrix} \mathbf{N}_{d,0}(\mathbf{k}) \\ \mathbf{N}_{d,1}(\mathbf{k}) \\ \mathbf{N}_{d,-1}(\mathbf{k}) \end{bmatrix}. \quad (25)$$

If we define $\mathbf{D}_{d,m}(\mathbf{k}) = \mathbf{S}(\mathbf{k} - m\mathbf{p}_\mathrm{d})O(\mathbf{k})$ and

$$M_d = \begin{bmatrix} 1 & \frac{c_{d,0}}{2} e^{i\varphi_{d,0}} & \frac{c_{d,0}}{2} e^{-i\varphi_{d,0}} \\ 1 & \frac{c_{d,1}}{2} e^{i\varphi_{d,1}} & \frac{c_{d,1}}{2} e^{-i\varphi_{d,1}} \\ 1 & \frac{c_{d,-1}}{2} e^{i\varphi_{d,-1}} & \frac{c_{d,-1}}{2} e^{-i\varphi_{d,-1}} \end{bmatrix}, \tag{26}$$

then $\mathbf{D}_{d,m}(\mathbf{k})$ can be separated by a 3×3 matrix manipulation:

$$\begin{bmatrix} \mathbf{D}_{d,0}(\mathbf{k}) \\ \mathbf{D}_{d,1}(\mathbf{k}) \\ \mathbf{D}_{d,-1}(\mathbf{k}) \end{bmatrix} = \begin{bmatrix} \mathbf{S}(\mathbf{k})O(\mathbf{k}) \\ \mathbf{S}(\mathbf{k}-\mathbf{p}_d)O(\mathbf{k}) \\ \mathbf{S}(\mathbf{k}+\mathbf{p}_d)O(\mathbf{k}) \end{bmatrix} = M_d^{-1} \begin{bmatrix} \mathbf{I}_{d,0}(\mathbf{k}) \\ \mathbf{I}_{d,1}(\mathbf{k}) \\ \mathbf{I}_{d,-1}(\mathbf{k}) \end{bmatrix} - M_d^{-1} \begin{bmatrix} \mathbf{N}_{d,0}(\mathbf{k}) \\ \mathbf{N}_{d,1}(\mathbf{k}) \\ \mathbf{N}_{d,-1}(\mathbf{k}) \end{bmatrix}. \tag{27}$$

The separated information components $D_{d,m}(\mathbf{k})$ with different pattern orientations $d = 1,2,3$ and phases $m = -1,0,1$ are then re-combined through a generalized Wiener filter:

$$\begin{aligned} \hat{\mathbf{Y}}(\mathbf{k}) &= \frac{\sum_{d,m} O^*(\mathbf{k}+m\mathbf{p}_d)\mathbf{D}_{d,m}(\mathbf{k}+m\mathbf{p}_d)}{\sum_{d,m}|O(\mathbf{k}+m\mathbf{p}_d)|^2+\omega^2}A(\mathbf{k}) \\ &= \frac{\sum_{d,m} O^*(\mathbf{k}+m\mathbf{p}_d)\left[\mathbf{I}'_{d,m}(\mathbf{k}+m\mathbf{p}_d) - \mathbf{N}'_{d,m}(\mathbf{k}+m\mathbf{p}_d)\right]}{\sum_{d,m}|O(\mathbf{k}+m\mathbf{p}_d)|^2+\omega^2}A(\mathbf{k}) \\ &= \frac{\sum_{d,m} O^*(\mathbf{k}+m\mathbf{p}_d)\mathbf{I}'_{d,m}(\mathbf{k}+m\mathbf{p}_d)}{\sum_{d,m}|O(\mathbf{k}+m\mathbf{p}_d)|^2+\omega^2}A(\mathbf{k}) - \\ &\quad \frac{\sum_{d,m} O^*(\mathbf{k}+m\mathbf{p}_d)\mathbf{N}'_{d,m}(\mathbf{k}+m\mathbf{p}_d)}{\sum_{d,m}|O(\mathbf{k}+m\mathbf{p}_d)|^2+\omega^2}A(\mathbf{k}), \end{aligned} \tag{28}$$

where $A(\mathbf{k})$ is a linear apodization function, which decreases linearly from unity at the origin to zero at the edge of the OTF; $\mathbf{I}'_{d,m}(\mathbf{k}+m\mathbf{p}_d)$ and $\mathbf{N}'_{d,m}(\mathbf{k}+m\mathbf{p}_d)$ are defined as

$$\begin{bmatrix} \mathbf{I}'_{d,0}(\mathbf{k}) \\ \mathbf{I}'_{d,1}(\mathbf{k}) \\ \mathbf{I}'_{d,-1}(\mathbf{k}) \end{bmatrix} = M_d^{-1} \begin{bmatrix} \mathbf{I}_{d,0}(\mathbf{k}) \\ \mathbf{I}_{d,1}(\mathbf{k}) \\ \mathbf{I}_{d,-1}(\mathbf{k}) \end{bmatrix}, \tag{29}$$

$$\begin{bmatrix} \mathbf{N}'_{d,0}(\mathbf{k}) \\ \mathbf{N}'_{d,1}(\mathbf{k}) \\ \mathbf{N}'_{d,-1}(\mathbf{k}) \end{bmatrix} = M_d^{-1} \begin{bmatrix} \mathbf{N}_{d,0}(\mathbf{k}) \\ \mathbf{N}_{d,1}(\mathbf{k}) \\ \mathbf{N}_{d,-1}(\mathbf{k}) \end{bmatrix}. \tag{30}$$

In Eq. (28), the first term is the noise-free SR-SIM reconstruction filtered by a

wiener parameter ω^2 (Supplementary Fig. 33a-c), and the second term is the noise-induced reconstruction artifacts, in which the noises were shifted and filtered by a Wiener filter in the Fourier domain (Supplementary Fig. 33d). If we denote the reconstruction noise by $\widehat{\mathbf{N}}(\mathbf{r})$, it can be formulated as

$$\widehat{\mathbf{N}}(\mathbf{r}) = \mathcal{F}^{-1}\left\{\frac{\sum_{d,m} O^*(\mathbf{k}+m\mathbf{p}_d)\mathbf{N}'_{d,m}(\mathbf{k}+m\mathbf{p}_d)}{\sum_{d,m}|O(\mathbf{k}+m\mathbf{p}_d)|^2+\omega^2}A(\mathbf{k})\right\} \tag{31}$$

If we define

$$\widetilde{H}(\mathbf{k}) = \left(\sum_{d,m}|O(\mathbf{k}+m\mathbf{p}_d)|^2+\omega^2\right)^{-1}, \tag{32}$$

the expression in Eq. (31) can be converted into the spatial domain as follows

$$\widehat{\mathbf{N}}(\mathbf{r}) = \widetilde{H}(\mathbf{r}) \otimes A(\mathbf{r}) \otimes \left[\sum_{d,m} e^{-i2\pi m\mathbf{p}_d\cdot\mathbf{r}} \cdot P(\mathbf{r}) \otimes e^{-i2\pi m\mathbf{p}_d\cdot\mathbf{r}} \cdot \sum_{m'} M^{-1}_{d,m,m'}\mathbf{N}_{d,m'}(\mathbf{r})\right], \tag{33}$$

where $\widetilde{H}(\mathbf{r})$ and $A(\mathbf{r})$ denote the inverse Fourier transform of $\widetilde{H}(\mathbf{k})$ and $A(\mathbf{k})$, and $M^{-1}_{d,m,m'}$ is the numerical element located at (m, m') of the phase separation matrix M_d^{-1}. Eq. (33) shows that the reconstruction noise $\widehat{\mathbf{N}}(\mathbf{r})$ is actually a linear combination of the original photon noise $\mathbf{N}_{d,m'}(\mathbf{r})$, in that both Hadamard product and convolution are linear operator with no offsets. As is discussed in Supplementary Note 1a, with sufficient fluorescence intensity, i.e., average photon counts larger than 3, the additive photon noise $\mathbf{N}_{d,m'}(\mathbf{r})$ can be modelled with a zero-mean Gaussian distribution, which consequently indicates the expectation of the reconstruction artifacts is also zero:

$$\mathbb{E}\{\widehat{\mathbf{N}}(\mathbf{r})\} = \mathbf{0}. \tag{34}$$

This zero-mean characteristics of reconstruction artifacts make it possible to perform denoising and deconvolution for SIM images in a 'noise2noise' manner[7], that is, if we use the noisy reconstruction $\widehat{\mathbf{Y}}(\mathbf{k})$ as the target in loss function of L_2-norm, the network output will converge to the expectation of it. According to Eqs. (28) and (34), the expectation of the noisy SIM reconstruction $\widehat{\mathbf{Y}}(\mathbf{k})$ is

$$\mathbb{E}\{\widehat{\mathbf{Y}}(\mathbf{k})\} = \frac{\sum_{d,m} O^*(\mathbf{k}+m\mathbf{p}_d)\mathbf{I}'_{d,m}(\mathbf{k}+m\mathbf{p}_d)}{\sum_{d,m}|O(\mathbf{k}+m\mathbf{p}_d)|^2+\omega^2}A(\mathbf{k}), \tag{35}$$

which is the same as its clear counterpart. However, it should be noticed that the Eq.

(35) is not the exactly ideal SIM reconstruction of the sample information $\mathbf{S}(\mathbf{k})$ but a filtered one by the residual wiener parameter ω^2 in Fourier domain. In practice, ω^2 is usually selected as a small value, e.g., 0.02 for 2D-SIM and 0.003 for 3D-SIM in our experiments, causing ignorable influence on the final reconstructed SR-SIM images compared with the theoretically ideal reconstruction, i.e., $\omega^2 = 0$ (Supplementary Fig. 33a-c).

In practical implementation of ZS-DeconvNet-SIM, we first added additional noises for each raw SIM images of different orientations and phases, i.e., 3-orientation × 3-phase, via Eqs. (3) and (4) to generate two sets of recorrupted raw SIM images, and then the generated images were reconstructed into two noisy SR-SIM images, denoted as $\widehat{\mathbf{Y}}$ and $\widetilde{\mathbf{Y}}$, which were used as the input and GT in the training procedure.

For the dual-stage architecture of ZS-DeconvNet-SIM (Supplementary Fig. 20a), we defined its overall loss function is of the same form with Eq. (11), and the denoising loss is calculated with the two recorrupted SIM images:

$$\mathcal{L}_{den}(\widehat{\mathbf{Y}}, \widetilde{\mathbf{Y}}) = \left\|f_{\boldsymbol{\theta}'}(\widehat{\mathbf{Y}}) - \widetilde{\mathbf{Y}}\right\|_2^2, \quad (36)$$

where $f_{\boldsymbol{\theta}'}$ is the denoising stage of ZS-DeconvNet-SIM with corresponding trainable parameters $\boldsymbol{\theta}'$. If we denote the reconstruction artifacts of $\widehat{\mathbf{Y}}$ and $\widetilde{\mathbf{Y}}$ by $\widehat{\mathbf{N}}$ and $\widetilde{\mathbf{N}}$, which are zero mean and independent from each other, then the expectation of Eq. (36) holds that

$$\begin{aligned}
&\mathbb{E}_{\widehat{\mathbf{Y}},\widetilde{\mathbf{Y}}}\left\{\left\|f_{\boldsymbol{\theta}'}(\widehat{\mathbf{Y}}) - \widetilde{\mathbf{Y}}\right\|_2^2\right\} \\
&= \mathbb{E}_{\widehat{\mathbf{N}},\widetilde{\mathbf{N}}}\left\{\left\|f_{\boldsymbol{\theta}'}(\mathbf{X} + \widehat{\mathbf{N}}) - (\mathbf{X} + \widetilde{\mathbf{N}})\right\|_2^2\right\} \\
&= \mathbb{E}_{\widehat{\mathbf{N}},\widetilde{\mathbf{N}}}\left\{\left\|f_{\boldsymbol{\theta}'}(\mathbf{X} + \widehat{\mathbf{N}}) - \mathbf{X}\right\|_2^2 - 2\widetilde{\mathbf{N}}^{\mathrm{T}}\left(f_{\boldsymbol{\theta}'}(\mathbf{X} + \widetilde{\mathbf{N}}) - \mathbf{X}\right) + \left\|\widetilde{\mathbf{N}}\right\|_2^2\right\} \\
&= \mathbb{E}_{\widehat{\mathbf{N}},\widetilde{\mathbf{N}}}\{\left\|f_{\boldsymbol{\theta}'}(\mathbf{X} + \widehat{\mathbf{N}}) - \mathbf{X}\right\|_2^2\} - \mathbb{E}_{\widehat{\mathbf{N}},\widetilde{\mathbf{N}}}\{2\widetilde{\mathbf{N}}^{\mathrm{T}}(f_{\boldsymbol{\theta}'}(\mathbf{X} + \widetilde{\mathbf{N}}) - \mathbf{X})\} + \mathrm{const} \\
&= \mathbb{E}_{\widehat{\mathbf{N}},\widetilde{\mathbf{N}}}\{\left\|f_{\boldsymbol{\theta}'}(\mathbf{X} + \widehat{\mathbf{N}}) - \mathbf{X}\right\|_2^2\} + \mathrm{const}, \quad (37)
\end{aligned}$$

where $\mathbf{X}$ is the noise-free SR-SIM reconstruction described in Eq. (35). Eq. (37) shows that the unsupervised loss function defined in Eq. (36) will lead to the same training results with that defined on noisy/clear SIM image pairs.

Similar to ZS-DeconvNet for acquired raw image processing, we next defined the deconvolution loss for ZS-DeconvNet-SIM based on recorrupted SIM image pairs and the super-resolution PSF matrix H_{SIM} as

$$\mathcal{L}_{dec}(\widehat{\mathbf{Y}}, \widetilde{\mathbf{Y}}) = \left\| H_{SIM} f_{\boldsymbol{\theta}}(\widehat{\mathbf{Y}}) - \widetilde{\mathbf{Y}} \right\|_2^2 + \lambda \mathcal{R}_{Hessian}\left(f_{\boldsymbol{\theta}}(\widehat{\mathbf{Y}})\right), \tag{38}$$

where $f_{\boldsymbol{\theta}}$ is the entire dual-stage network with all trainable parameters $\boldsymbol{\theta}$. Let $\boldsymbol{S}$ be the fluorescent sample, then the expectation of the first term in Eq. (38) suffices that

$$\begin{aligned}
&\mathbb{E}_{\widehat{\mathbf{Y}},\widetilde{\mathbf{Y}}}\left\{\left\| H_{SIM} f_{\boldsymbol{\theta}}(\widehat{\mathbf{Y}}) - \widetilde{\mathbf{Y}} \right\|_2^2\right\} \\
&= \mathbb{E}_{\widehat{\mathbf{N}},\widetilde{\mathbf{N}}}\left\{\left\| H_{SIM} f_{\boldsymbol{\theta}}(\mathbf{X} + \widehat{\mathbf{N}}) - \mathbf{X} \right\|_2^2 - 2\widetilde{\mathbf{N}}^{\mathrm{T}}\left(H_{SIM} f_{\boldsymbol{\theta}}(\mathbf{X} + \widehat{\mathbf{N}}) - \mathbf{X}\right) + \left\|\widetilde{\mathbf{N}}\right\|_2^2\right\} \\
&= \mathbb{E}_{\widehat{\mathbf{N}},\widetilde{\mathbf{N}}}\{\left\| H_{SIM} f_{\boldsymbol{\theta}}(\mathbf{X} + \widehat{\mathbf{N}}) - \mathbf{X} \right\|_2^2\} - \mathbb{E}_{\widehat{\mathbf{N}},\widetilde{\mathbf{N}}}\{2\widetilde{\mathbf{N}}^{\mathrm{T}}(H_{SIM} f_{\boldsymbol{\theta}}(\mathbf{X} + \widehat{\mathbf{N}}) - \mathbf{X})\} + \mathrm{const} \\
&= \mathbb{E}_{\widehat{\mathbf{N}},\widetilde{\mathbf{N}}}\{\left\| H_{SIM} f_{\boldsymbol{\theta}}(\mathbf{X} + \widehat{\mathbf{N}}) - H_{SIM}\mathbf{S} \right\|_2^2\} + \mathrm{const} \\
&= \mathbb{E}_{\widehat{\mathbf{N}},\widetilde{\mathbf{N}}}\{\left\| H_{SIM} (f_{\boldsymbol{\theta}}(\mathbf{X} + \widehat{\mathbf{N}}) - \mathbf{S}) \right\|_2^2\} + \mathrm{const}.
\end{aligned} \tag{39}$$

Eq. (39) shows that the minimization of the proposed deconvolution loss will optimally lead to the solution that satisfies $f_{\boldsymbol{\theta}^*}(\mathbf{X} + \widehat{\mathbf{N}}) = \boldsymbol{S}$, which demonstrates that the ZS-DeconvNet-SIM models are trained to super-resolve the clear sample directly from the noisy SIM reconstructions. It is noted that the illumination pattern parameters which are used to reconstruct $\widehat{\mathbf{Y}}$ and $\widetilde{\mathbf{Y}}$ should be identical so that the expectation of $\widehat{\mathbf{Y}}$ and $\widetilde{\mathbf{Y}}$ are also same, i.e., $\mathbf{X}$, which is important to the stable convergency of ZS-DeconvNet-SIM models during training.

d. 3D ZS-DeconvNet-SIM

The applications of 3D ZS-DeconvNet-SIM for volumetric SIM modalities such as lattice light-sheet structured illumination microscopy (LLS-SIM) are similar to those of 3D ZS-DeconvNet described in Supplementary Note 1b with the primary difference being that 3D ZS-DeconvNet-SIM adopts spatially interleaved post-reconstructed SIM images rather than noisy raw images as inputs and GT in both training and inference phases (Supplementary Fig. 20b). The objective function of 3D ZS-DeconvNet-SIM is devised as the combination of the denoising loss and the deconvolution loss, which is formulated as follows

$$\mathcal{L}(\mathbf{Z}) = \mu\mathcal{L}_{den}(\mathbf{Z}) + (1-\mu)\mathcal{L}_{dec}(\mathbf{Z}), \tag{40}$$

$$\begin{aligned}\mathcal{L}_{den}(\mathbf{Z}) = &\left\| f_{\boldsymbol{\theta}'}(\widehat{\mathbf{Z}}) - \widetilde{\mathbf{Z}} \right\|_2^2 \\ +\gamma &\left\| f_{\boldsymbol{\theta}'}(\widehat{\mathbf{Z}}) - \widetilde{\mathbf{Z}} - \left(S_{odd}(f_{\boldsymbol{\theta}'}(\mathbf{Z})) - S_{even}(f_{\boldsymbol{\theta}'}(\mathbf{Z}))\right) \right\|_2^2,\end{aligned} \tag{41}$$

$$\mathcal{L}_{dec}(\mathbf{Z}) = \left\| H_{SIM} f_{\boldsymbol{\theta}}(\hat{\mathbf{Z}}) - \tilde{\mathbf{Z}} \right\|_2^2$$
$$+\gamma \left\| H_{SIM} f_{\boldsymbol{\theta}}(\hat{\mathbf{Z}}) - \tilde{\mathbf{Z}} - S_{odd}\left(f_{\boldsymbol{\theta}'}(\mathbf{Z})\right) + S_{even}\left(f_{\boldsymbol{\theta}'}(\mathbf{Z})\right) \right\|_2^2$$
$$+\lambda \mathcal{R}_{Hessian}\left(f_{\boldsymbol{\theta}}(\hat{\mathbf{Z}})\right), \tag{42}$$

where $\mathbf{Z}$, $\hat{\mathbf{Z}}$, and $\tilde{\mathbf{Z}}$ are the entire stack, odd slices, and even slices of the noisy SIM image stack generated via the analytical SIM reconstruction algorithm, H_{SIM} is the volumetric PSF of corresponding SIM systems, and other symbols are defined as same as Eqs. (16), (17), and (19). Owing to the linearity of SIM reconstruction, Eq. (34) is compatible for LLS-SIM as well. Based on the zero-mean property of noise-induced reconstruction artifacts and independence of the noise between adjacent axial layers, the following holds with similar derivation procedures to Eqs. (18) and (20):

$$\begin{aligned}
&\mathbb{E}_{\mathbf{Z}}\left\{\left\| f_{\boldsymbol{\theta}'}(\hat{\mathbf{Z}}) - \tilde{\mathbf{Z}} \right\|_2^2\right\} \\
&= \mathbb{E}_{\mathbf{Z}}\left\{\left\| f_{\boldsymbol{\theta}'}(\hat{\mathbf{Z}}) - \hat{\mathbf{X}} + \hat{\mathbf{X}} - \tilde{\mathbf{Z}} \right\|_2^2\right\} \\
&= \mathbb{E}_{\mathbf{Z}}\left\{\left\| f_{\boldsymbol{\theta}'}(\hat{\mathbf{Z}}) - \hat{\mathbf{Z}} \right\|_2^2\right\} + \mathbb{E}_{\mathbf{Z}}\left\{2\left(f_{\boldsymbol{\theta}'}(\hat{\mathbf{Z}}) - \hat{\mathbf{X}}\right)^{\mathrm{T}}\left(\hat{\mathbf{X}} - \tilde{\mathbf{Z}}\right)\right\} + \mathbb{E}_{\mathbf{Z}}\left\{\left\|\hat{\mathbf{X}} - \tilde{\mathbf{Z}}\right\|_2^2\right\} \\
&= \mathbb{E}_{\mathbf{Z}}\left\{\left\| f_{\boldsymbol{\theta}'}(\hat{\mathbf{Z}}) - \hat{\mathbf{X}} \right\|_2^2\right\} + 2\boldsymbol{\varepsilon}^{\mathrm{T}}\mathbb{E}_{\mathbf{Z}}\left\{\mathrm{f}_{\boldsymbol{\theta}}(\hat{\mathbf{Z}}) - \hat{\mathbf{X}}\right\} + \mathbb{E}_{\mathbf{Z}}\{\boldsymbol{\varepsilon}^{\mathrm{T}}\boldsymbol{\varepsilon}\} + \mathrm{const} \\
&\rightarrow \mathbb{E}_{\mathbf{Z}}\left\{\left\| f_{\boldsymbol{\theta}'}(\hat{\mathbf{Z}}) - \hat{\mathbf{X}} \right\|_2^2\right\} + \mathrm{const}(\boldsymbol{\varepsilon} \rightarrow \mathbf{0}),
\end{aligned} \tag{43}$$

$$\begin{aligned}
&\mathbb{E}_{\mathbf{Z}}\left\{\left\| H_{SIM} f_{\boldsymbol{\theta}}(\hat{\mathbf{Z}}) - \tilde{\mathbf{Z}} \right\|_2^2\right\} \\
&= \mathbb{E}_{\mathbf{Z}}\left\{\left\| H_{SIM} f_{\boldsymbol{\theta}}(\hat{\mathbf{Z}}) - \hat{\mathbf{X}} + \hat{\mathbf{X}} - \tilde{\mathbf{Z}} \right\|_2^2\right\} \\
&= \mathbb{E}_{\mathbf{Z}}\left\{\left\| H_{SIM} f_{\boldsymbol{\theta}}(\hat{\mathbf{Z}}) - \hat{\mathbf{X}} \right\|_2^2\right\} + 2\mathbb{E}_{\mathbf{Z}}\left\{\left(H_{SIM} f_{\boldsymbol{\theta}}(\hat{\mathbf{Z}}) - \hat{\mathbf{X}}\right)^{\mathrm{T}}\right\}\mathbb{E}_{\mathbf{Z}}\left\{\hat{\mathbf{X}} - \tilde{\mathbf{Z}}\right\} + \mathbb{E}_{\mathbf{Z}}\{\boldsymbol{\varepsilon}^{\mathrm{T}}\boldsymbol{\varepsilon}\} + \mathrm{const} \\
&= \mathbb{E}_{\mathbf{Z}}\left\{\left\| H_{SIM}\left(f_{\boldsymbol{\theta}}(\hat{\mathbf{Z}}) - \hat{\mathbf{S}}\right) \right\|_2^2\right\} + 2\boldsymbol{\varepsilon}^{\mathrm{T}}\mathbb{E}_{\mathbf{Z}}\left\{H_{SIM} f_{\boldsymbol{\theta}}(\hat{\mathbf{Z}}) - \hat{\mathbf{X}}\right\} + \mathbb{E}_{\mathbf{Z}}\{\boldsymbol{\varepsilon}^{\mathrm{T}}\boldsymbol{\varepsilon}\} + \mathrm{const} \\
&\rightarrow \mathbb{E}_{\mathbf{Z}}\left\{\left\| H_{SIM}\left(f_{\boldsymbol{\theta}}(\hat{\mathbf{Z}}) - \hat{\mathbf{S}}\right) \right\|_2^2\right\} + \mathrm{const}(\boldsymbol{\varepsilon} \rightarrow \mathbf{0}),
\end{aligned} \tag{44}$$

where $\hat{\mathbf{X}}$ and $\hat{\boldsymbol{S}}$ denote the odd slices of the ideal SR-SIM image stack and the fluorescent sample; $\boldsymbol{\varepsilon}$ is the gap between odd and even slices which satisfies $\boldsymbol{\varepsilon} = \hat{\mathbf{X}} - \tilde{\mathbf{X}} = \mathbb{E}_{\mathbf{Z}}\{\hat{\mathbf{X}} - \tilde{\mathbf{Z}}\}$. Similar to 3D ZS-DeconvNet, the gap-amending regularization is applied in both denoising loss and deconvolution loss to eliminate the none-zero expectation of the deviation between $\hat{\mathbf{Z}}$ and $\tilde{\mathbf{Z}}$, and the continuity regularization is used in deconvolution loss to regularization the convergence of network training.

2. Simulation of dynamic samples and images

a. Simulation of ground truth image of tubular structure

We generate the ground truth (GT) image of tubular structures following our previously described procedures[6], which briefly consists of three steps:

(i) Randomly generate positional parameters of tubules and sketch them on a blank image by using the MATLAB function of randi() and insertshape();

(ii) Assign a random elastic deformation field of 4×4 elements and resize it to the same size as the generated image of tubular structure with bicubic interpolation;

(iii) Apply the resized elastic deformation field on the sample image to bend the tubular structures by using the MATLAB function of interp2().

To mimic the active dynamics of microtubules in live cells and generate corresponding time-lapsing image sequence, we simulate a displacement for each simulated tubules between adjacent two frames. Specifically, an initial sample image is generated by the above steps, and then assign a displacement of $D_{i,t}$ along a randomly specified orientation for each simulated tubule i at each time point t through the MATLAB function imwarp(). The displacement $D_{i,t}$ is defined as

$$D_{i,t} = \frac{1 + 2u_{i,t}}{3} v, \tag{45}$$

where $u_{i,t}$ is randomly sampled from the uniform distribution $\mathrm{U}[0,1]$, and v is the pre-defined velocity parameter representing the moving speed of current image sequence. The pixel size of the generated sample images is 31.3 nm, which is set to be one half the pixel size of our Multi-SIM system.

b. Simulation of noisy diffraction-limited wide-field images

To simulate the diffraction-limited wide-field (WF) images of punctate and tubular structures, the GT image is first convolved with a simulated wide-field PSF, which is generated by Fiji plugin of PSF Generator following Born and Wolf optical model with the same imaging configurations of our experiment, where the emission wave length is 525 nm, the effective detection NA equals to 1.3. The PSF is normalized by dividing the summation of all its pixel values before subsequent calculation. After convolution, the simulated WF image I is down-sampled by two-fold via bicubic interpolation in order to match the pixel size of experimentally acquired images and then rescaled to a specified signal level signal by performing:

$$\mathbf{I}_{WF} = \text{signal} \cdot \frac{\mathbf{I}}{\text{prctile}(\mathbf{I}, 99.9)}, \tag{46}$$

where $\text{prctile}(\mathbf{I}, 99.9)$ returns the intensity value ranking 0.1% in $\mathbf{I}$. Finally, the rescaled WF image is contaminated with Poisson noise and Gaussian noise as follows:

$$\mathbf{I}'_{WF} = \text{Poiss}(\mathbf{I}_{WF}) + \text{Gauss}(b, \sigma_G^2), \tag{47}$$

where $\text{Poiss}(\cdot)$ denotes Poisson corruption implemented by the MATLAB function poissrnd(), and $\text{Gauss}(b, \sigma_G^2)$ denotes Gaussian noise with a mean value of $b = 100$ and a standard deviation of $\sigma_G = 3.5$, which are measured from single background frame of our sCMOS camera. The noisy WF image $\mathbf{I}'_{WF}$ and its clear counterpart $\mathbf{I}_{WF}$ are used to evaluate the related computational SR methods in Supplementary Figs. 1, 3-9, 12.

3. Fiji integration for ZS-DeconvNet

a. General consideration

Although ZS-DeconvNet and other deep-learning (DL) based image restoration approaches have dramatically pushed the limits of fluorescence live-cell imaging, it might be currently not convenient enough to build a virtual environment for Tensorflow or Pytorch and execute Python code for researchers in life science community, especially on operating systems like Windows or MacOS. To make our ZS-DeconvNet more accessible to researchers with various backgrounds, we developed a Java-based plugin for ZS-DeconvNet methods which can be installed in Fiji[8], the commonly used image processing and analysing platform in the biomedical imaging and life science community. In contrast to other Fiji plugins developed for implementation of DL-based image processing methods, which mostly load models pre-trained from Python codes and perform inference only, we integrated functionalities of both training and inference based on the pioneering open-source Java framework CSBDeep developed for image denoising methods CARE[9], noise2void[10] and BioImage Model Zoo[11]. Ideally, our released plugin enables one-click training and inference of ZS-DeconvNet even for users or researchers without much DL model training experience.

Moreover, we build up a tutorial homepage for ZS-DeconvNet and corresponding Fiji plugin (https://tristazeng.github.io/ZS-DeconvNet-page/Tutorial/), which includes the introduction and detailed implementation instruction for both Python and Fiji plugin. In following subsections, we mainly introduce the general design and workflow of training and inference deployments, other detailed instruction can be found in our tutorial homepage and Github repository (https://github.com/TristaZeng/ZS-DeconvNet/blob/main/Fiji_Plugin/ReadMe.md).

b. Training with ZS-DeconvNet Fiji plugin

The ZS-DeconvNet Fiji plugin was developed based on TensorFlow-Java 1.15.0, which is compatible with CUDA version of 10.1 and cuDNN version of 7.5.1. The instructions for environment setup and plugin installation can be found in the 5th part *How to use our Fiji plugin* in the tutorial homepage of ZS-DeconvNet. After installation, the plugins can be found in the Fiji menu under *Plugins* > *ZS-DeconvNet.*

For ZS-DeconvNet model training, we generally provide two commands: *train augmented data* and *train on opened images*, which differ in the ways of data loading and augmentation. The former command loads input data and corresponding GT images

which are augmented elsewhere, e.g., in MATLAB or Python, from two data folders file by file, and the latter command directly takes the image stack opened in the current Fiji window as the training data and automatically perform data augmentation including image recorruption (for 2D cases), random cropping, rotation and flipping into a pre-specified patch number. The overall workflow of ZS-DeconvNet training with Fiji plugin is schematized in Supplementary Fig. 26, which includes following steps:

(i) Open the image or stack to be used for training in Fiji and start the ZS-DeconvNet plugin by clicking *Plugins > ZS-DeconvNet > train on opened imgs*; or directly start the plugin by the alternative command *Plugins > ZS-DeconvNet > train on augmented data* and select the folders containing input images, GT images, and validation images.

(ii) Select the network type, i.e., 2D ZS-DeconvNet or 3D ZS-DeconvNet, the PSF file used for calculating deconvolution loss and choose training hyperparameters, which includes *total augmentation number, total epochs, iteration number per epoch*, *batch size*, and *initial learning rate*. For 2D ZS-DeconvNet training by the command of *train on opened images*, three extra recorruption-related parameters of α, β_1, and β_2 are tuneable, where α and β_1 are set as [1, 2] and [0.5, 1.5] by default, and β_2 should be set as the square of the standard deviation of the camera background, which could be pre-calibrated from blank frames or calculated from empty regions of the training data.

(iii) Click *OK* to start training. During the training procedure, the training progress and current learning rate will be displayed in a message box, and the model will be validated after each training epoch with the validation input and output shown in another image window for reference. After training finished, the trained model could be saved manually in the selected directory with the format of BioImage Model Zoo bundle.

c. Inference with ZS-DeconvNet Fiji plugin

Given a pre-trained ZS-DeconvNet model and an image or stack to be processed, the Fiji plugin is able to generate the corresponding denoised (optional) and super-resolved deconvolution image. The schematic workflow of SR image inference via ZS-DeconvNet plugin is shown in Supplementary Fig. 27, which includes following steps:

(i) Open the image or stack in Fiji and start ZS-DeconvNet plugin by Clicking *Plugins > ZS-DeconvNet > predict ZS-DeconvNet 2D / predict ZS-DeconvNet 3D.*

(ii) Select the network model file, i.e., .zip file in the format of BioImage Model

Zoo bundle. Of note, the model file could be trained and saved either by Python codes or ZS-DeconvNet Fiji plugin.

(iii) Check inference options and choose hyperparameters used in the inference. The options and parameters here are primarily selected to properly normalize the input data (*NormalizeInput*, *PercentileBottom*, and *PercentileTop*), perform tiling prediction to save memory of CPUs or GPUs (*Number of tiles*, *Overlap between tiles*, and *Batch size*), and decide whether to show process dialog and denoising results or not (*Show process dialog* and *Show denoising result*). More detailed description on the functionality of these options and parameters could be found in our tutorial homepage.

(iv) After image processing with status bar shown in the message box (if select *Show process dialog*), the denoised (if select *Show denoising result*) and deconvolved output will pop out in separate Fiji windows automatically. Then the processed images could be viewed, manipulated, and saved via Fiji.

4. Parameters in the ZS-DeconvNet software

As have been demonstrated in recent literatures[2, 12], incorporation of certain physical properties of the imaging system can rationalize the training and inference process of deep neural networks and provide substantial improvements in output fidelity and resolution. In the development of ZS-DeconvNet, we fully exploited the physically pre-determined prior knowledges including the optical imaging forward model, PSF, properties of camera background, and spatial continuity prior for biological images, which are theoretically independent of the biological specimen types or fluorescent signal levels. Each of them is associated with one or more hyper-parameters, i.e., the PSF file, the weighting scalars defined in the loss function, and the noise related parameters. In addition, there are several training parameters such as the initial learning rate, patch size, and total epochs to be assigned before each training trial, which influence the convergence of the network models. All of these hyper-parameters can be classified into three categories: parameters defined in the loss function, recorruption parameters and training parameters. In this note, we intend to provide a comprehensive description of the parameters for users to guide an effective usage of the ZS-DeconvNet software. For more succinct descriptions and suggested values of the parameters, please refer to Supplementary Table 4.

a. Parameters defined in the loss function

There are four parameters defined in the loss function, including:

PSF: The point spread function of the optical system, which is used for calculating the deconvolution loss in the ZS-DeconvNet models. The best option of the PSF is the measured beads because the experimentally acquired beads describe the actual imaging process best. But if the imaging system is well calibrated, i.e., with the least optical aberrations and its PSF is very close to the theoretical one, the simulated PSF can be applied as well. The simulated PSF can be generated by (i) using the Matlab script we uploaded on GitHub for PSF generation or (ii) using the PSF Generator Fiji plugin licensed by EPFL (https://bigwww.epfl.ch/algorithms/psfgenerator/). Of note, the PSF is normalized before the calculation by dividing the summation of its intensity in the software to ensure the output deconvolved image is conservative in terms of intensity (Supplementary Fig. 7e-g).

μ: The scalar weight to balance the denoising loss and deconvolution loss. We have tested the deconvolution performance of ZS-DeconvNet on both 2D and 3D dataset with a large range of μ values. Both representative deconvolved images and the statistical comparison show that the ZS-DeconvNet keeps a stable performance with

$\mu/(1-\mu)$ ranging from 0.01 to 10 (Supplementary Fig. 29). Therefore, we suggest a μ value of 0.5 for all circumstances, which is also used in all our experiments.

λ: The scalar weight to balance the Hessian regularization in the loss function. Different from some existing analytical algorithms, e.g., Hessian-SIM[13], where the Hessian regularization is utilized to suppress the noises and needs to be carefully chosen in handling images of different SNRs, the Hessian regularization used in the training process of ZS-DeconvNet is mainly to mitigate the slight pixelized artifact (Supplementary Fig. 1b). Therefore, the hessian weighting scalar does not need to be tuned for image of each SNR (Supplementary Fig. 5). In all experiments of this paper, we set λ to a small value of 0.02 for 2D models and 0.1 for 3D models.

γ: The scalar weight to balance the gap amending regularization (GAR) in the loss function. The GAR[2] is applied in the 3D ZS-DeconvNet models to compensate the non-zero expectation gap of the self-supervised denoising and deconvolution loss, which is not related to any prior-knowledges of the specimen but a technical trick to regulate the spatially interleaved self-supervised (SiS) training procedure. As is the same with the original paper, we adopted a γ value of 1 for all experiments in this paper.

b. Recorruption parameters

The image recorruption strategy is applied to generate the training dataset for 2D ZS-DeconvNet, where there are three parameters, including:

β_1: The Poissonian factor that affects the variance of the signal-dependent shot noise in the image recorruption process for 2D ZS-DeconvNet. The theoretically optimal value of β_1 is 1 for imaging conditions (Supplementary Note 1a), which has been validated with synthetic images of beads and tubular structures of different signal levels (Supplementary Figs. 3, 4). Nevertheless, we found that for experimental data, a random value of β_1 within a small range, e.g., [0.5, 1.5], for each training patch pairs in the recorruption process achieves a stronger robustness and is applicable for various biological specimens and imaging conditions.

β_2: The Gaussian factor that represents the variance of the additive Gaussian noises, i.e., the readout noise of the camera, which can be estimated from the sample-free region of the images in training dataset or pre-calibrated from the camera following standard protocols[4]. In the python implementation and Fiji plugin of ZS-DeconvNet, the β_2 can be either automatically estimated from the training dataset or manually defined as a more accurate value calibrated elsewhere (Supplementary Fig. 26).

α: The noise magnification factor, which controls the overall magnitude of the

added noises. The value of α does not affect the independence of the noise in the paired recorrupted images as shown in Eq. 10, thereby any values are theoretically applicable. However, in practice, to avoid over-corruption for either the input or target images (Supplementary Fig. 3, 4), we adopted a modest range of α, i.e., [1, 2], for all 2D ZS-DeconvNet models, which is applicable for both simulated and experimental dataset of various specimens and imaging conditions in this paper.

c. Training parameters

There are four key parameters for the training procedure of ZS-DeconvNet models, which are also common hyper-parameters for any deep neural network models. We will give some empirical instructions and suggestions in tuning these parameters when training a new ZS-DeconvNet model. The specific training parameters used in this paper are also summarized in Supplementary Table 1.

Initial learning rate: The learning rate determines the step size at each iteration during the network training, which decays by a factor of 0.5 every 50 epochs from the initial value in the implementations of ZS-DeconvNet. A higher initial learning rate typically leads to faster convergence of the model, while destabilizes the training process. Therefore, we empirically set the initial learning rate as 0.5×10^{-4} and 1×10^{-4} for 2D and 3D models, respectively.

Patch size: The patch size determines the image shape after data augmentation, which may affect the total training time and final performance of the trained network models. We have tested the training time and performance of 2D/3D ZS-DeconvNet models trained with dataset of different patch sizes. As is shown in Supplementary Fig. 34, the training duration goes longer as the training patch size gets larger but without obvious increasement in validation PSNR. Therefore, we typically choose a relatively small patch size of 128×128 pixels for 2D models and $64 \times 64 \times 13$ voxels for 3D models to speed up the training process, which are also the default settings in our python implementations and the Fiji plugin.

Batch size: The batch size is defined as the number of samples used for each training iteration, which mainly affects the convergence speed and generalization of the network models. Generally, a batch size that is either too large or too small may raise difficulties in the training procedure, e.g., out of memory error or unstable convergence. In our experiments of this paper, we adopted a modest batch size of 4 for 2D models and 3 for 3D models to balance the convergence speed, memory usage, and the model generalization, which are applied in all our experiments of this paper and robust enough for most applications.

Total epoch: The total epoch determines the total training iterations (total epoch × iterations per epoch). In the training procedure of ZS-DeconvNet, the latest model is evaluated using partial training images, e.g., 2%, after each training epoch. Therefore, the iterations per epoch actually affect the frequency of the model validation during training. Typically, the total epoch × iterations per epoch are set as 250 × 200 for 2D models and 100 × 100 for 3D models, which are sufficient to ensure the network convergence in most experiments.

Supplementary Figures

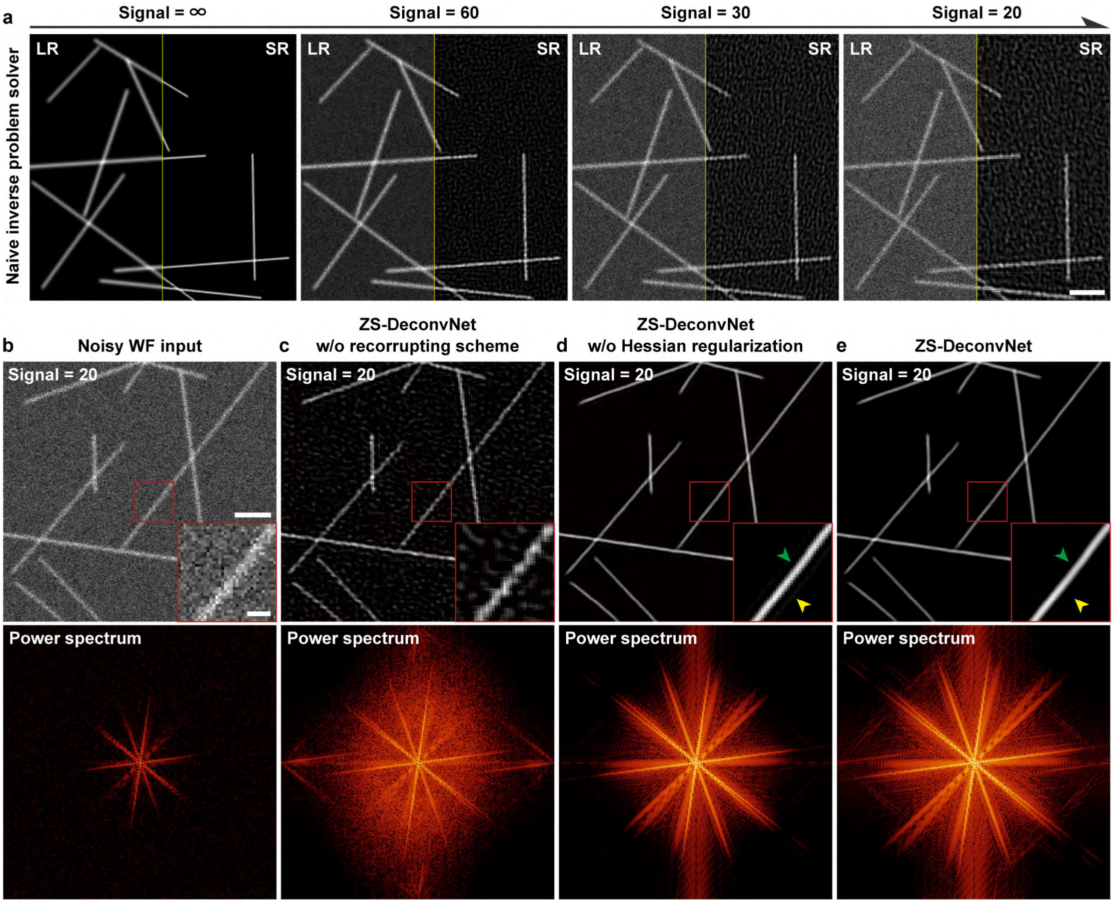

Supplementary Fig. 1 | Ablation study of ZS-DeconvNet. a, SR performance for simulated microtubule images with the naïve inverse problem solver under decreasing signal levels of infinite, 60, 30, and 20 (Supplementary Note 2). The displayed images of low resolution (LR, left half) and super-resolution (SR, right half) show that the naïve inverse problem solver produces escalating noise-induced artifacts as the signal level decreases. **b-e**, Noisy wide-field image (b) and output images via ZS-DeconvNet (e) and ZS-DeconvNet without the recorrupting scheme (c) and the Hessian regularization (d) under the signal level of 20. The corresponding Fourier power spectrums are shown below, indicating ZS-DeconvNet equipped with both the recorrupting scheme and the Hessian regularization recovers finest HR information. Scale bar, 2 μm (a-e), 0.5 μm (zoom-in regions in b-e).

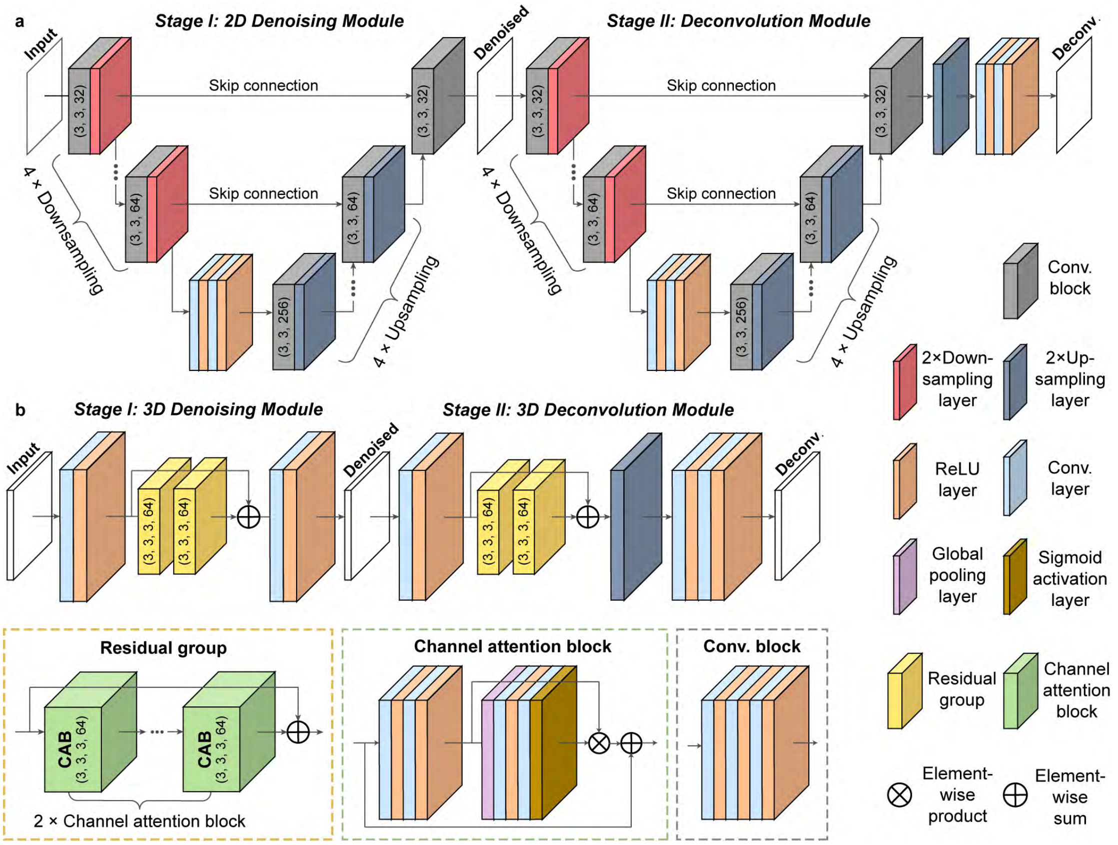

Supplementary Fig. 2 | Network architectures of ZS-DeconvNet and 3D ZS-DeconvNet. a, The dual-stage architecture of ZS-DeconvNet used for processing 2D images, which is composed of two sequentially connected U-net models and an up-sampling module including an up-sampling layer and a convolution block. **b**, The dual-stage architecture of 3D ZS-DeconvNet for volumetric data processing. Each stage is composed of a modified 3D residual channel attention network with two residual groups consisting two channel attention blocks. An optional up-sampling module is used to up-sample the feature maps and generate the final monochrome grayscale SR stack.

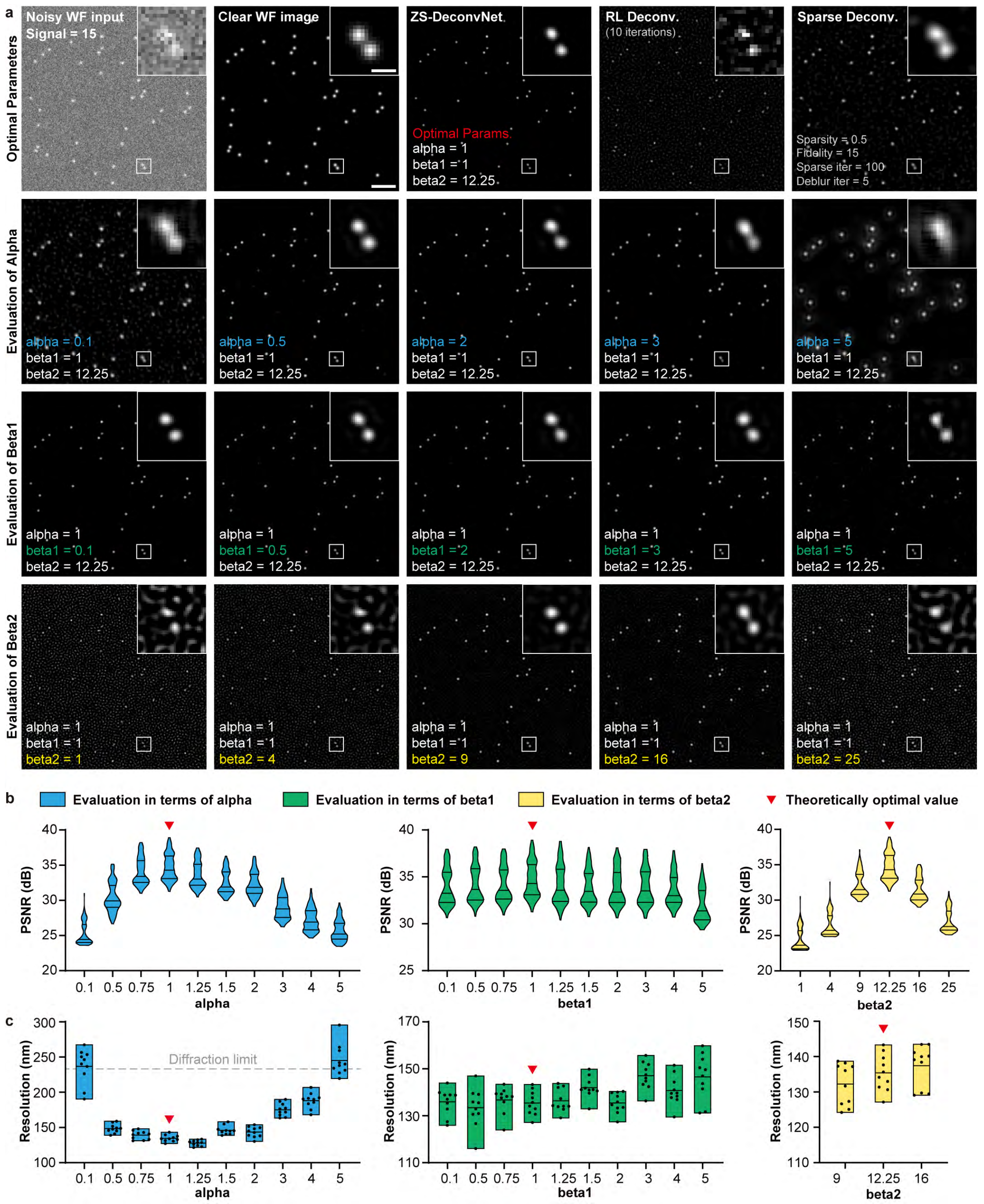

Supplementary Fig. 3 | Optimal hyperparameter validation for ZS-DeconvNet on simulated images of punctate structures. a, Images of punctate structures generated by RL deconvolution, sparse deconvolution and ZS-DeconvNet with different hyperparameter choices in terms of alpha (the second row), beta1 (the third row), and beta2 (the fourth row). The noisy wide-field (WF) image was simulated following the steps described in Supplementary Note 2 with a signal level of 15. The diffraction-limited clear image is provided for reference. **b, c**, Statistical evaluations of PSNR (b, n=100) and resolution (c, n=10) for ZS-DeconvNet trained with different hyperparameter choices. The theoretically optimal values for each parameter are labelled with red triangles and the theoretical diffraction limit is labelled with gray dashed lines in c. The resolution was measured with the full width at the half maximum (FWHM) of isolated beads. Both qualitive and quantitative assessments indicate that the experimentally optimal hyperparameters are well consistent with the theoretical analysis in Supplementary Note 1. Source data are provided as a Source Data file. Scale bar, 2 μm (a), 0.4 μm (zoom-in regions of a).

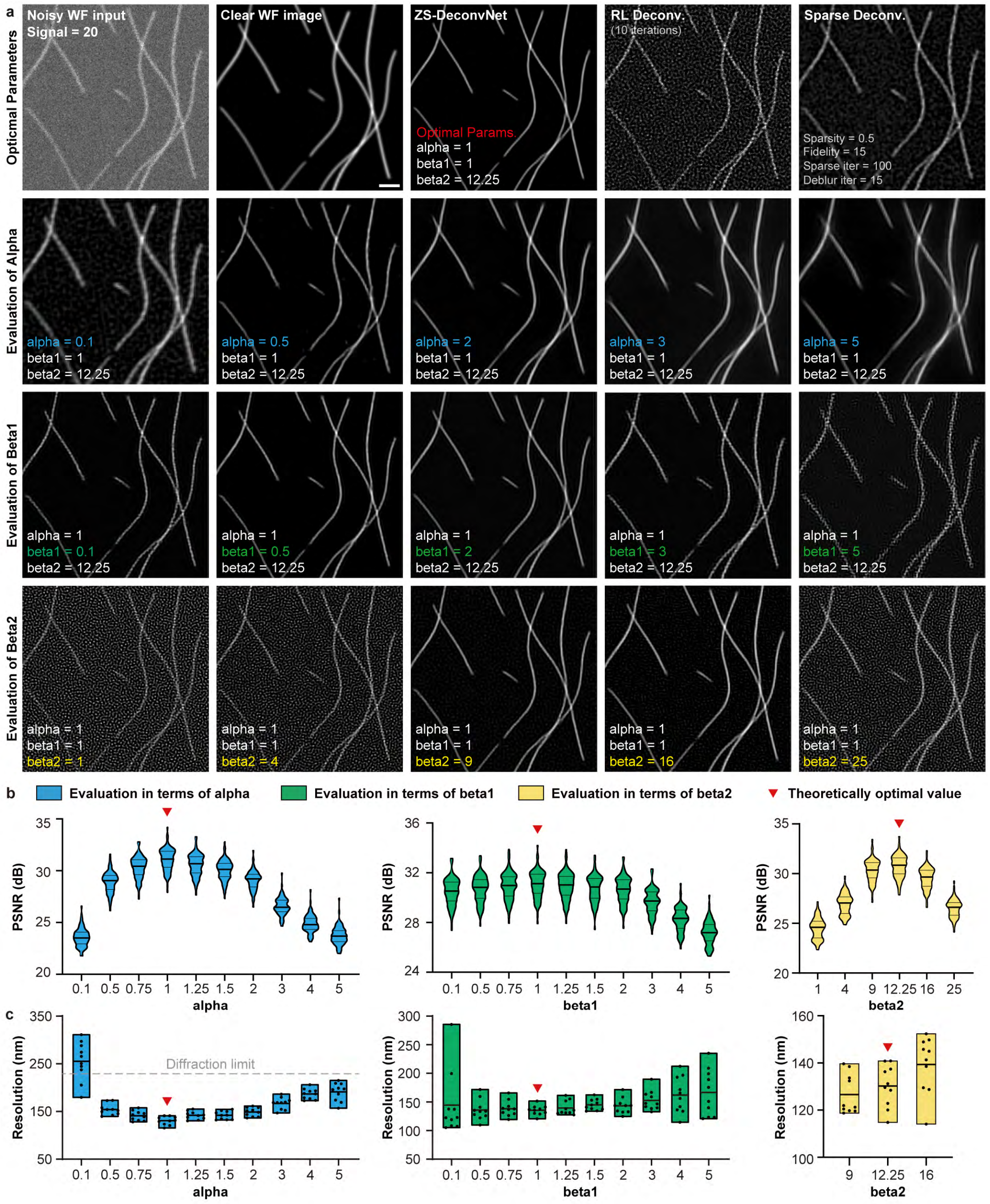

Supplementary Fig. 4 | Optimal hyperparameter validation for ZS-DeconvNet on simulated images of tubular structures. a, Images of tubular structures generated by RL deconvolution, sparse deconvolution and ZS-DeconvNet with different hyperparameter choices in terms of alpha (the second row), beta1 (the third row), and beta2 (the fourth row). The noisy wide-field (WF) image was simulated following the steps described in Supplementary Note 2 with a signal level of 20. The diffraction-limited clear image is provided for reference. **b, c**, Statistical evaluations of PSNR (b, n=100) and resolution (c, n=10) for ZS-DeconvNet trained with different hyperparameter choices. The theoretically optimal values for each parameter are labelled with red triangles and the theoretical diffraction limit is labelled with gray dashed lines in c. The resolution was evaluated with the full width at the half maximum (FWHM) of isolated microtubules. Both qualitive and quantitative assessments indicate that the experimentally optimal hyperparameters are well consistent with the theoretical analysis in Supplementary Note 1. Source data are provided as a Source Data file. Scale bar, 1.5 μm.

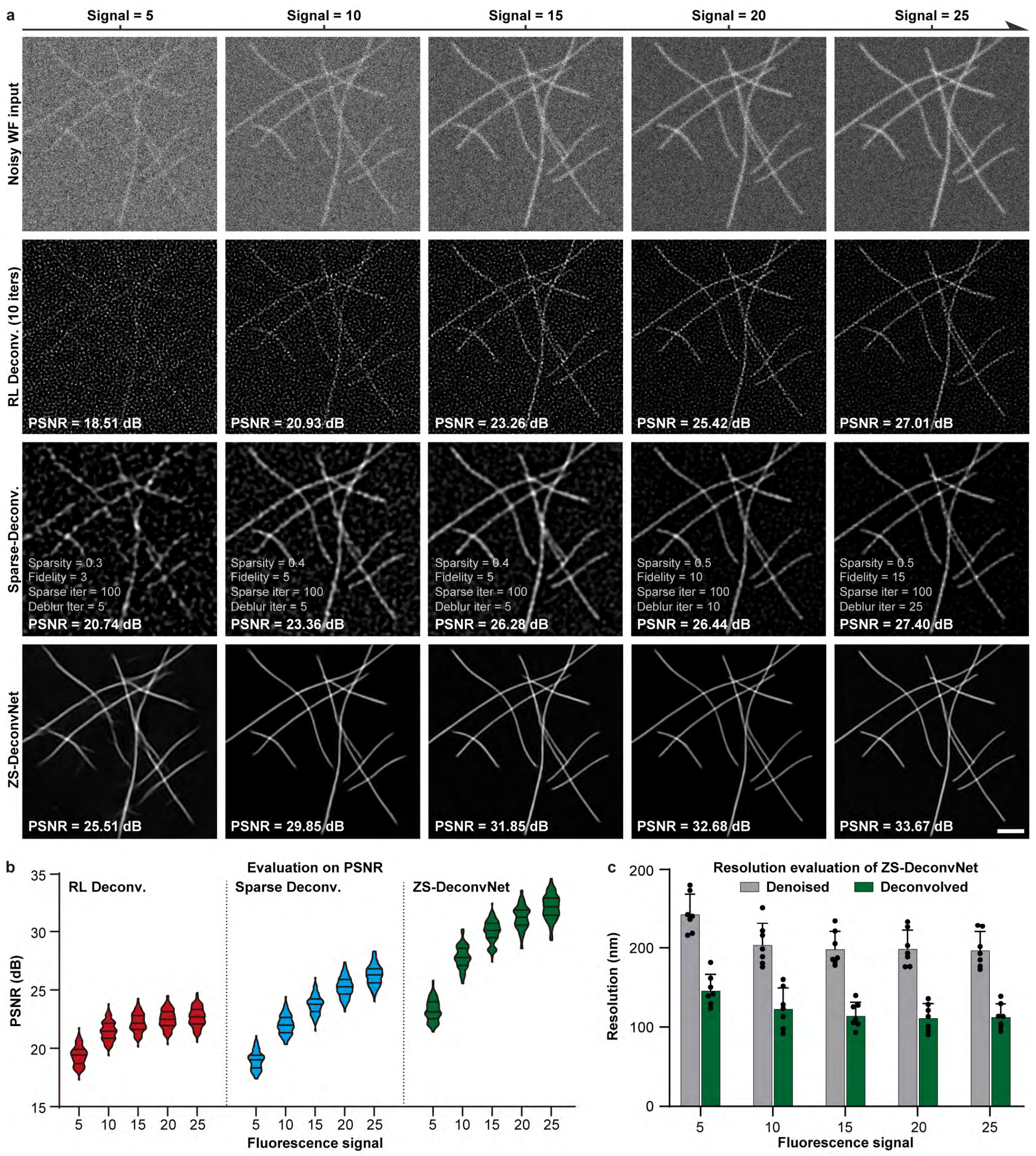

Supplementary Fig. 5 | Evaluation of ZS-DeconvNet at different fluorescence signal level. a, Deconvolved images generated via RL deconvolution (the second row), sparse deconvolution (the third row), and ZS-DeconvNet (the fourth row) from noisy wide-filed inputs at fluorescence signal level ranging from 5 to 25 (Supplementary Note 2). Scale bar, 2 μm. **b**, PSNR comparisons of RL deconvolution (red), sparse deconvolution (blue), and ZS-DeconvNet (green) at different fluorescence signal levels (n=100). These results show that ZS-DeconvNet performs state-of-the-art deterministic approaches under various imaging conditions. In particular, the optimal parameters of sparse deconvolution should be manually tuned for each signal level to balance noise suppression and structural sharpness (the third row in a), while the ZS-DeconvNet used the same hyperparameters for images of all signal levels. **c**, Resolution evaluation for images output by the denoising stage and deconvolution stage of ZS-DeconvNet at different fluorescence signal levels, which were measured with the FWHM of sectioned profiles (n=7). These results indicate that in the dual-stage network architecture of ZS-DeconvNet, the first stage of ZS-DeconvNet serves as a denoiser to recover noise-contaminated information and improve the resolution to the level of diffraction limits, then the second stage further enhances the resolution by more than 1.5-fold over the diffraction limits. Source data are provided as a Source Data file.

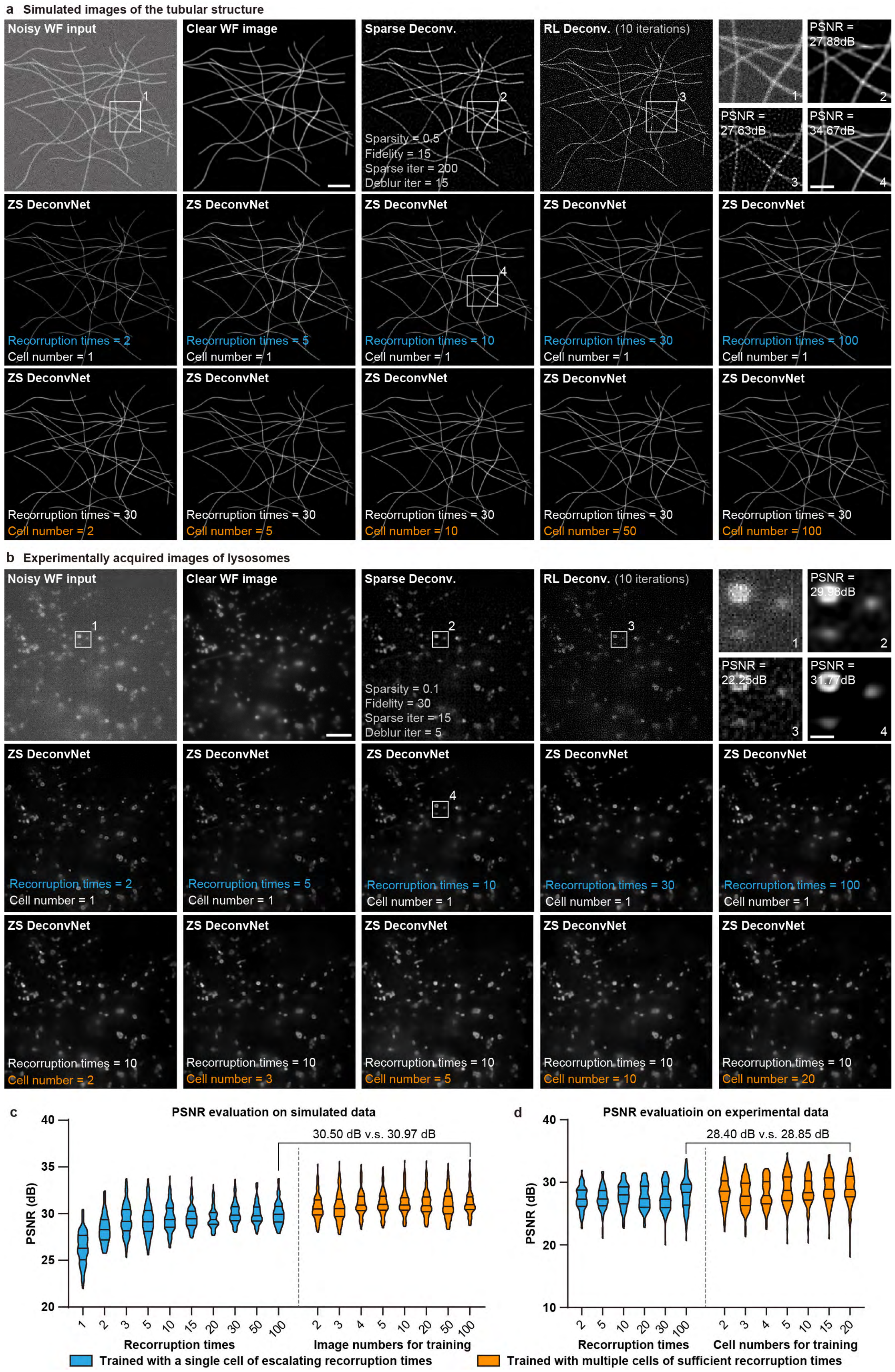

Supplementary Fig. 6 | Evaluation and characterization of ZS-DeconvNet trained with different augmentation strategies and scales of dataset. a, Deconvolved images of simulated tubular structures enhanced by RL deconvolution, sparse deconvolution, and ZS-DeconvNet trained on a single image with

different recorruption times (ranging from 2 to 100) and different amounts of images (ranging from 2 to 100) with a fixed recorruption time of 30. The diffraction limited clear image is shown for comparison. **b**, Deconvolved images of lysosomes enhanced by RL deconvolution, sparse deconvolution, and ZS-DeconvNet trained on a single image with different recorruption times (ranging from 2 to 100) and different amounts of images (ranging from 1 to 20) with a fixed recorruption time of 10. The diffraction limited clear image is shown for comparison. **c**, **d**, Statistical evaluation of ZS-DeconvNet on simulated data (c, n=100) and experimental data (d, n=100) in terms of PSNR trained with different recorruption times (blue) and escalating scales of dataset (orange). Of note, the ZS-DeconvNet models trained with a single image recorrupted with a sufficient time show comparable performance to the models trained with abundant training dataset for both simulated and experimental data, i.e., decreasing less than 0.5 dB in PNSR, indicating the capability and robustness of ZS-DeconvNet even trained with a single input image. Source data are provided as a Source Data file. Scale bar, 4 μm (a, b), 1.5 μm (zoom-in region in a), 0.4 μm (zoom-in region in b).

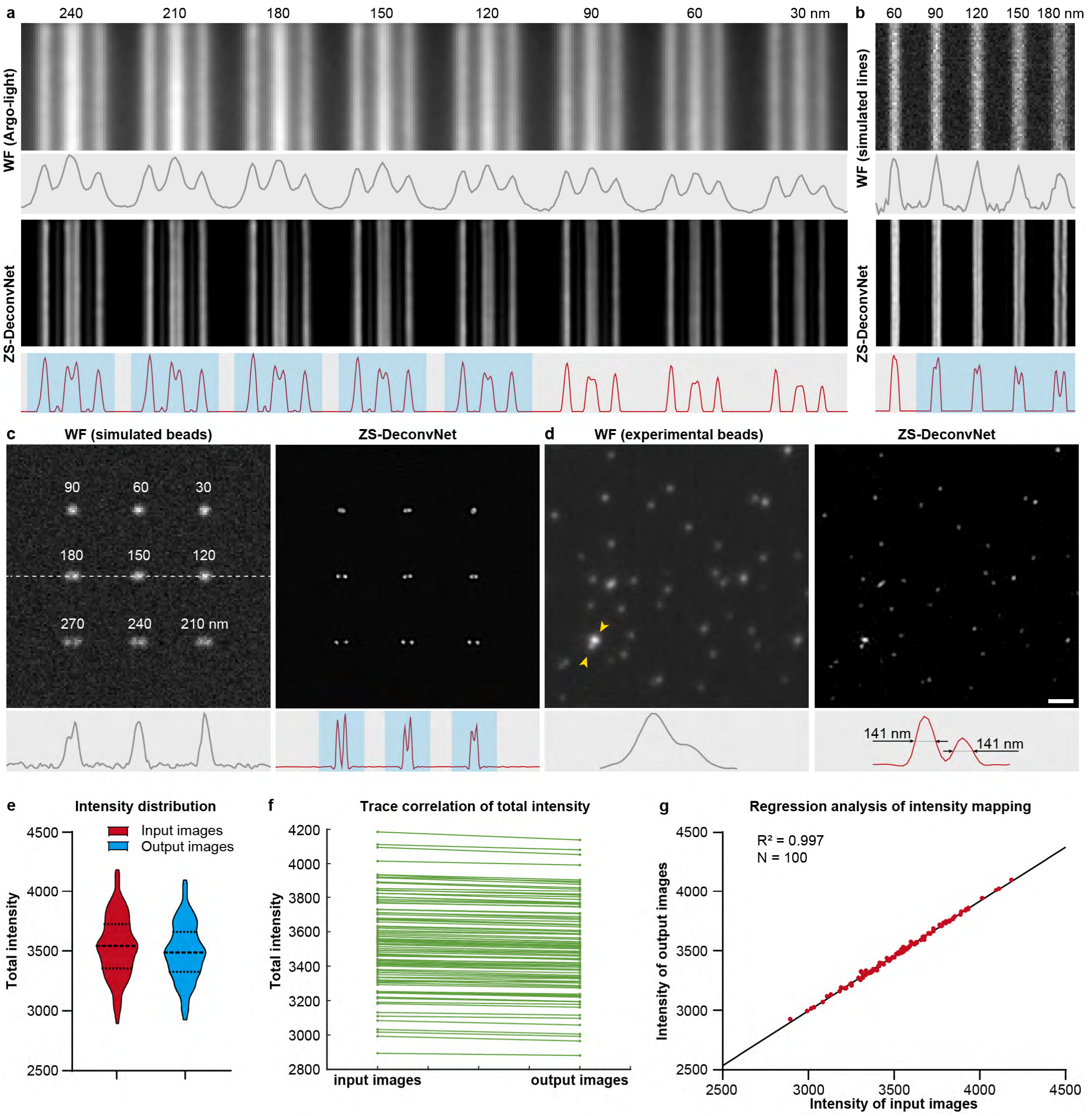

Supplementary Fig. 7 | Evaluation on resolution enhancement and quantifiability of ZS-DeconvNet. **a**, Wide-filed (upper row) and ZS-DeconvNet enhanced images (lower row) of Argo-SIM slides consisting of 8 pairs of dual lines, whose spacing gradually increases from 30 nm to 240 nm with a step of 30 nm. **b**, Wide-filed (upper row) and ZS-DeconvNet enhanced images (lower row) of synthetic parallel microtubules placed 60, 90, 120, 150, and 180 nm apart. The intensity profiles along the horizontal axis are shown below the images. These results show that ZS-DeconvNet is able to distinguish two parallel slides with interline spacing down to 90~120 nm (highlighted with blue regions), which is one obscure line in the original wide-field image. **c**,**d**, Wide-filed (left panel) and ZS-DeconvNet enhanced images (right panel) of synthetic (c) and experimental (d) beads. The intensity profiles along lines indicated by the white dashed line (c) or yellow arrow heads (d) are plotted below the images. Both of the image and intensity profiles demonstrate that ZS-DeconvNet models can separate two synthetic beads with a displacement of down to 120 nm or resolve two neighboring fluorescent beads distributed closer than the diffraction limits. Scale bar, 1 µm. **e**,**f**, Total intensity distribution and trace correlation of input wide-field images and processed images by ZS-DeconvNet (n=100). **g**, Regression analysis between the total intensity of input and output images by ZS-DeconvNet (n=100). These statistical analyses show a high consistency between the total signal levels of the input and output images by ZS-DeconvNet, which indicates the intensity conservation capability of the network model and the quantifiability of the output images. Source data are provided as a Source Data file.

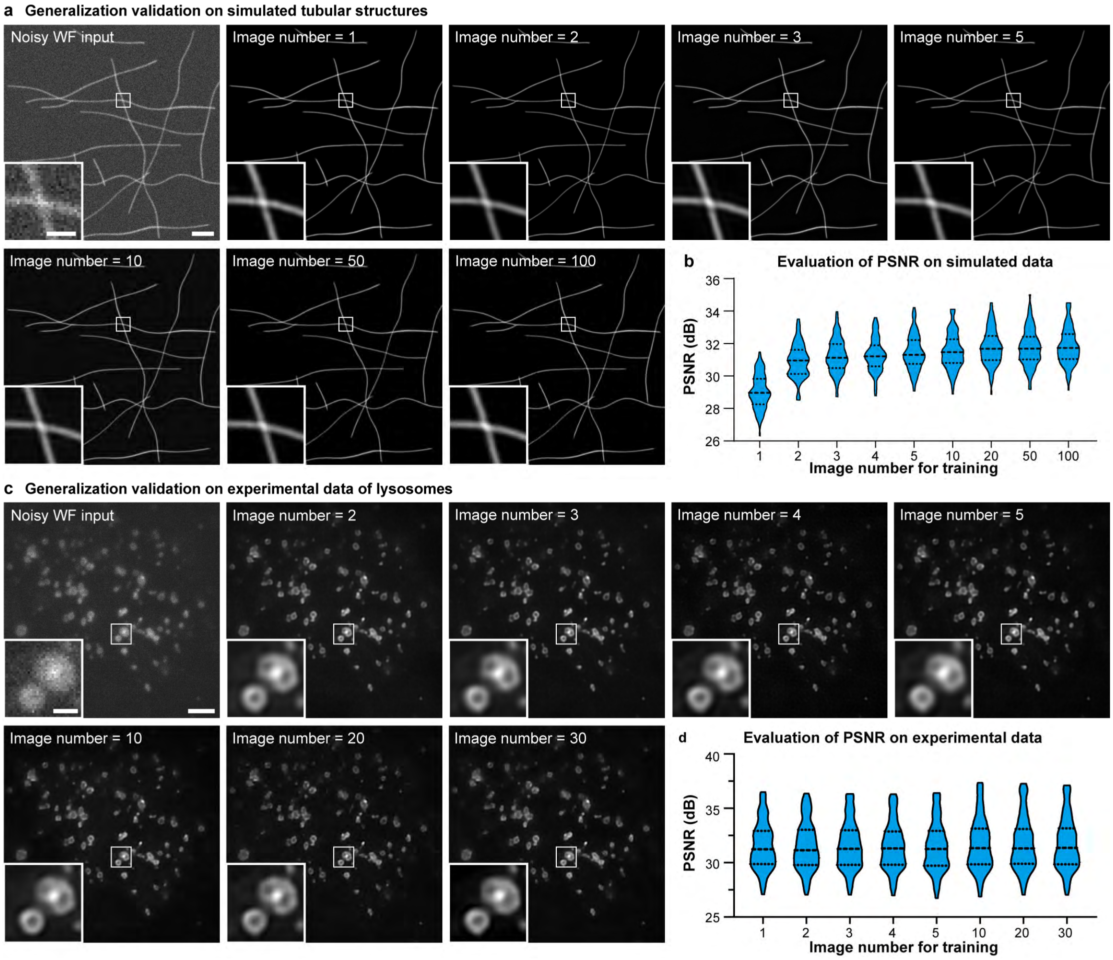

Supplementary Fig. 8 | Generalization validation of ZS-DeconvNet models trained with dataset of different scales. a,**b**, Representative images (a) of tubular structure processed by ZS-DeconvNet models train with simulated dataset of escalating scales ranging from 1 to 100 images and corresponding PSNR evaluation (b) of these models (n=100). **c**,**d**, Representative images (c) of lysosomes processed by ZS-DeconvNet models train with experimental dataset of escalating scales ranging from 1 to 30 images and corresponding PSNR evaluation (d) of these models (n=100). The testing images used in a-d were not included in the training dataset. These results indicate that larger dataset scales generally lead to stronger generalization capability of ZS-DeconvNet models, and a training dataset consisting of 5~20 images provides a balanced option between model generalization capability and dataset scales. Source data are provided as a Source Data file. Scale bar, 3 μm (a, c), 0.7 μm (zoom-in regions of a and c).

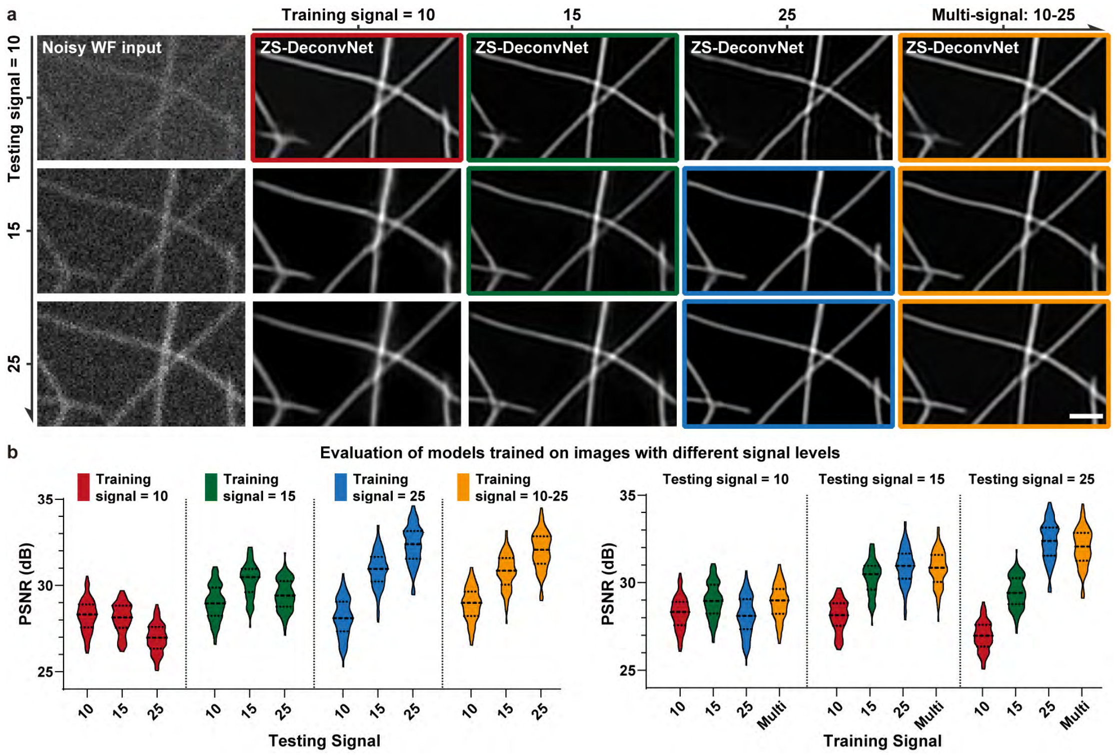

Supplementary Fig. 9 | Generalization validation of ZS-DeconvNet models on SNR shifts between training and testing data. Three individual ZS-DeconvNet models were trained on synthetic data of tubular structures with different signal levels of 10, 15, and 25, respectively. Another model was trained with images of mixed signal levels ranging from 10 to 25. Representative input images with different signal levels (first column in **a**), enhanced images by the trained ZS-DeconvNet models (second to fifth columns in **a**), and statistical evaluation in terms of PSNR of these models (**b**) are shown here (n=100). These results suggest that for testing data of a specific SNR, the model trained with data of similar or a little higher signal levels generally provide a superior performance in terms of PSNR and perceptual quality. Meanwhile, the model trained with data of all signal levels gave the best generalization capability and presented a good performance on testing data of all SNRs. Source data are provided as a Source Data file. Scale bar, 1 μm.

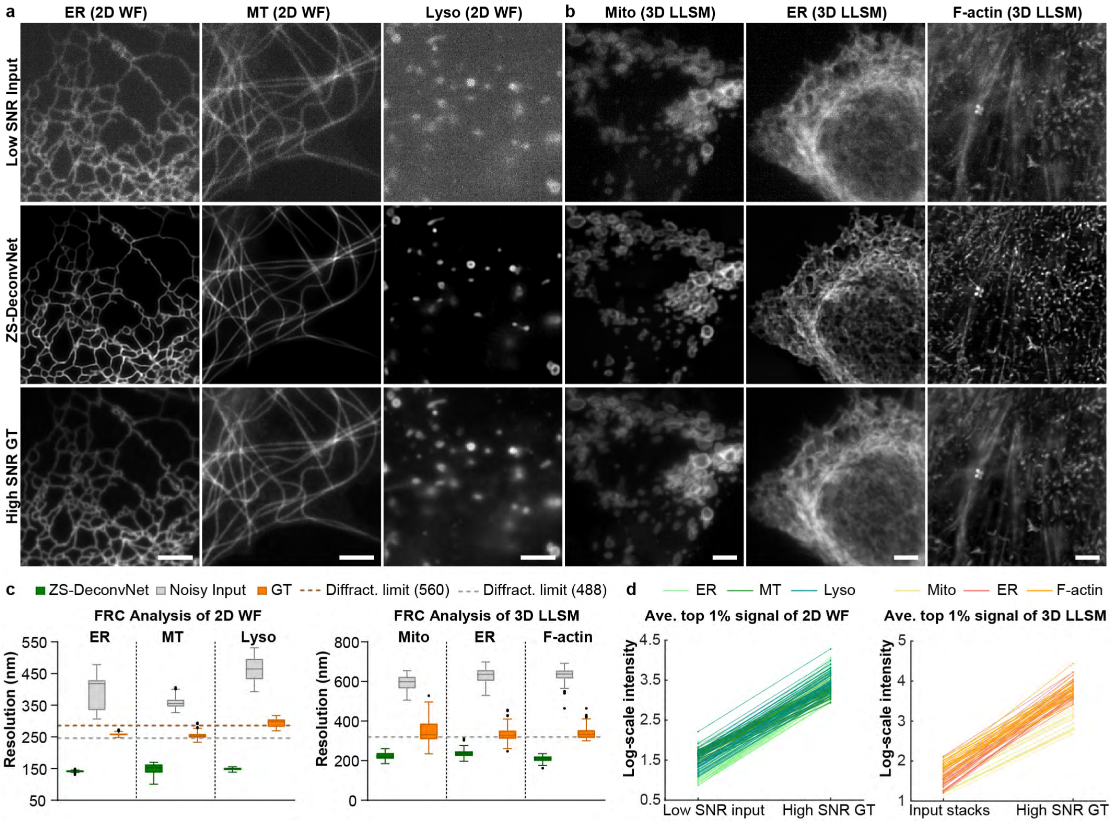

Supplementary Fig. 10 | Resolution and fluorescence intensity quantification for dataset and ZS-DeconvNet enhanced images. a, **b**, Representative 2D (a) and 3D (b, MIP) noisy input images, high SNR GT images, and ZS-DeconvNet enhanced images of ER, mitochondria, lysosomes, and microtubules. Scale bar, 3 μm. **c**, Lateral resolution comparison by FRC analysis of noisy input images, high SNR GT images and ZS-DeconvNet enhanced images (n=120). The diffraction limits for excitation wavelength of 488 nm (for ER and MT, gray dashed lines) and 560 nm (for lyso, orange dashed line) are labelled for reference. **d**, Comparison of fluorescence intensity between low SNR input images for ZS-DeconvNet and high SNR GT data of which the signal level is typically used in routine SR imaging (n=160 for 2D WF, n=76 for 3D LLSM). The signal intensity is quantified by averaging sCMOS counts of top 1% pixels for each image or stack. Each line represents a low- and high-SNR pair of 2D images or 3D stacks, showing that the fluorescence intensity of high SNR GT data is more than 10-fold higher than that of noisy input data of ZS-DeconvNet. Source data are provided as a Source Data file.

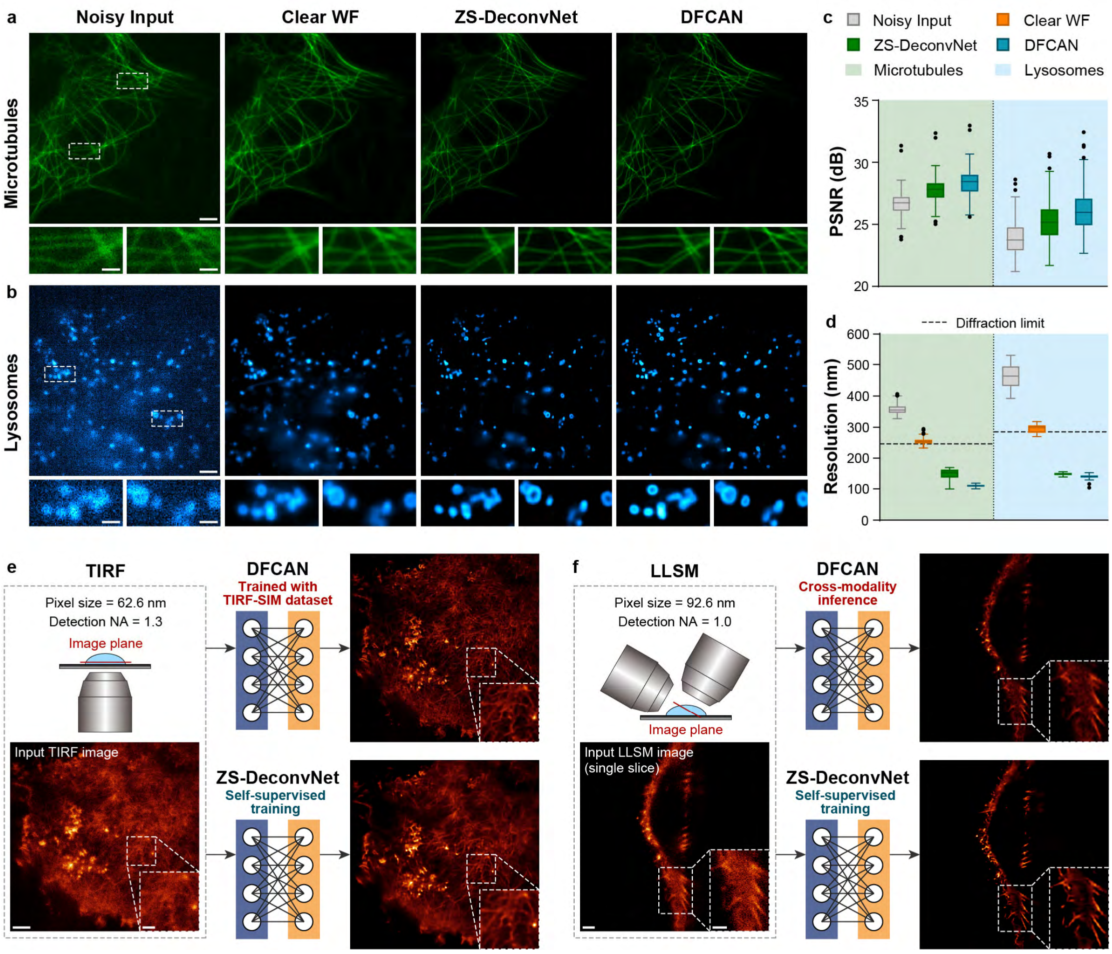

Supplementary Fig. 11 | Comparison of ZS-DeconvNet with DFCAN in terms of fidelity, resolution, and generalization. a, **b**, Representative microtubule (a) and lysosome (b) images of low (first column) and high SNR (second column) or reconstructed by ZS-DeconvNet (third column) or DFCAN (fourth column). **c**, **d**, Statistical comparison of ZS-DeconvNet and DFCAN in terms of PSNR (c, n=120) and FRC resolution (d, n=120). Source data are provided as a Source Data file. **e**, Representative SR TIRF images reconstructed by DFCAN (trained with TIRF/TIRF-SIM image pairs) and ZS-DeconvNet (trained with only noisy TIRF images). **f**, Representative SR LLSM single slice image reconstructed by DFCAN (the same model used in e, because there is no isotropic GT-SIM data to train a new model) and ZS-DeconvNet (trained with noisy LLSM images themselves). The comparisons shown in e and f indicate that the unsupervised ZS-DeconvNet can be well-generalized to a new data type without additional training dataset, while the conventional supervised SR model such as DFCAN suffers from the performance degradation due to the domain shift problem. Scale bar, 3 μm (a, b, e, f), 1 μm (zoom-in regions of a, b, e), 2 μm (zoom-in regions of f).

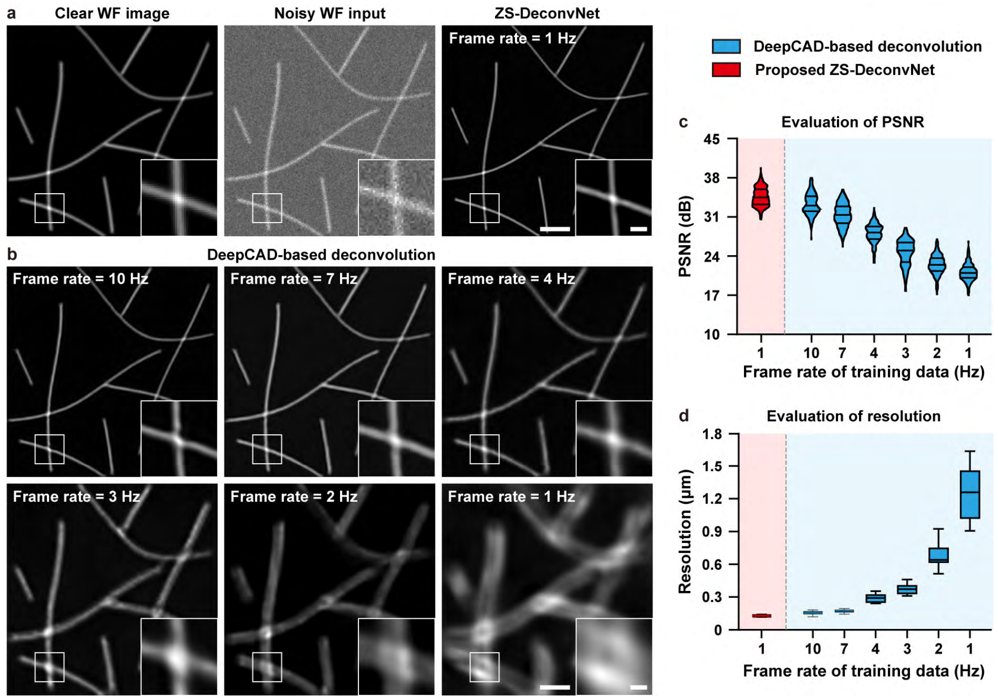

Supplementary Fig. 12 | Comparison of ZS-DeconvNet and DeepCAD-based deconvolution networks on simulated time-lapse images of tubular structures. a, Representative wide-field frame with/without noises and the corresponding ZS-DeconvNet enhanced image from a simulated 1000-frame video of tubular structures at a 1Hz frame rate. The average moving speed of simulated microtubules is set to ~1 μm/s (Supplementary Note 2), which is approximately consistent with the growth velocity of microtubules in live COS-7 cells[14]. The ZS-DeconvNet model was trained with all frames of the video. **b**, Deconvolved outputs of the same image shown in a, which were produced with DeepCAD-based deconvolution networks (Methods) trained on simulated time-lapse data of different imaging frame rate ranging from 1 Hz to 10 Hz. **c**, **d**, Statistical comparison of ZS-DeconvNet used in a (red) and DeepCAD-based deconvolution networks used in b (blue) in terms of (c) PSNR (n=190) and (d) resolution (n=15). The resolution was evaluated with the FWHM of microtubules and the theoretical diffraction limit is labelled with gray dashed lines in d. These results together suggest that ZS-DeconvNet can be well-trained with time-lapse data lacking of temporal continuity, yielding superior inference fidelity and resolution compared with recently proposed self-supervised learning schemes for microscopy data, i.e., DeepCAD. Source data are provided as a Source Data file. Scale bar, 2 μm (a, b), 0.5 μm (zoom-in regions of a and b).

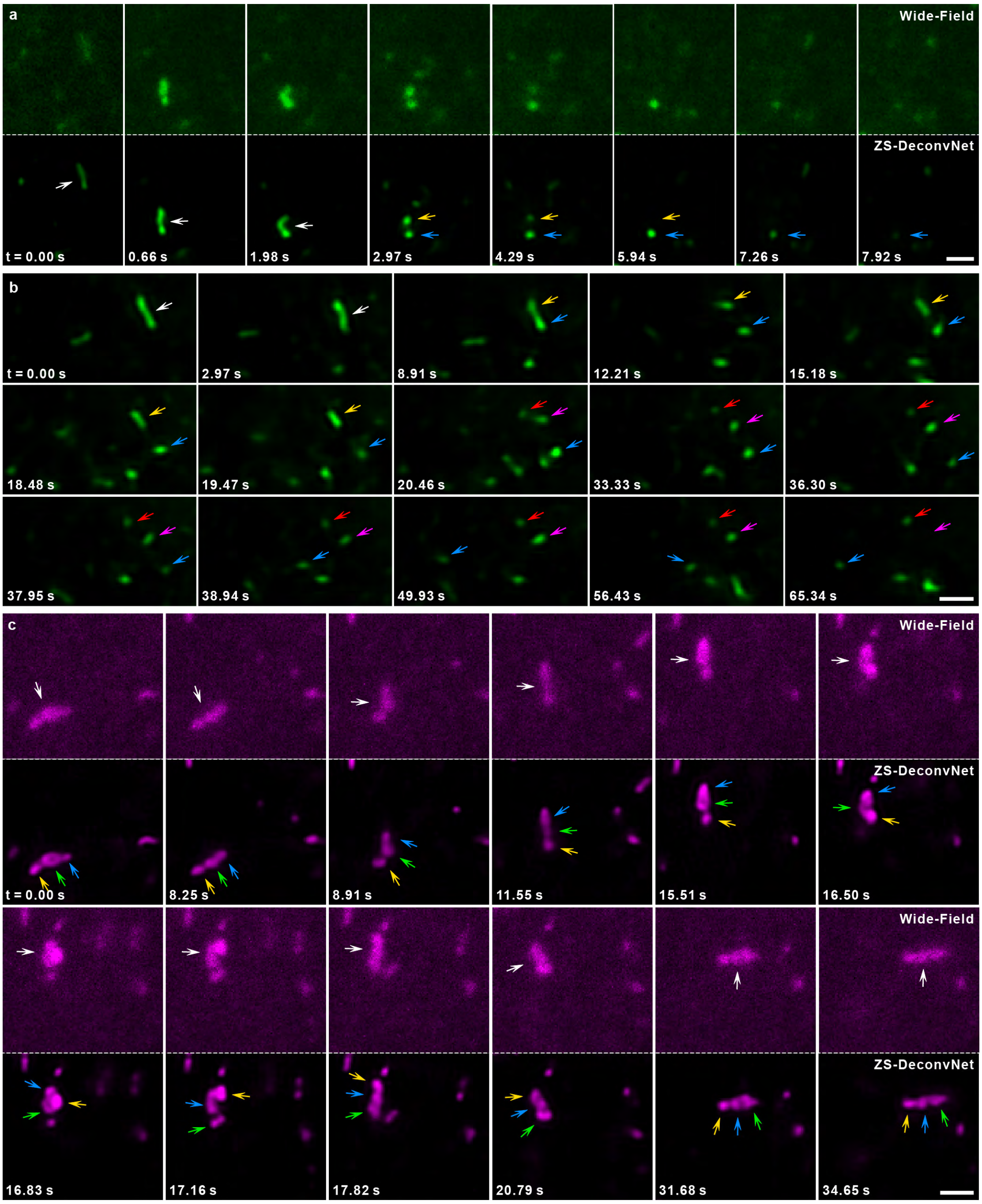

Supplementary Fig. 13 | Interesting showcases of the dynamics of recycling endosomes (REs) and lysosomes or late-endosomes (Lyso/LEs) in live SUM159 cells revealed by ZS-DeconvNet. a, Time-lapse images of a fission event of RE, and both of the divided REs undergo exocytosis sequentially (indicated by yellow and blue arrows, respectively). The corresponding noisy wide-field images are provided in the upper row for comparison. **b**, Time-lapse images of another fission event of RE, in which a tubular RE divides into three parts and one of them (indicated by blue arrows) ran away independently. **c**, Time-lapse images of three tethered Lyso/LEs traveling together, and rearranging their sequence in the period of 15.51 to 17.82 secondes. The corresponding noisy wide-field images are shown in the upper rows, where the tethering details and the rearrangement of the three Lyso/LEs can hardly be recognized. Scale bar, 1 μm (a, b), 1.5 μm (c).

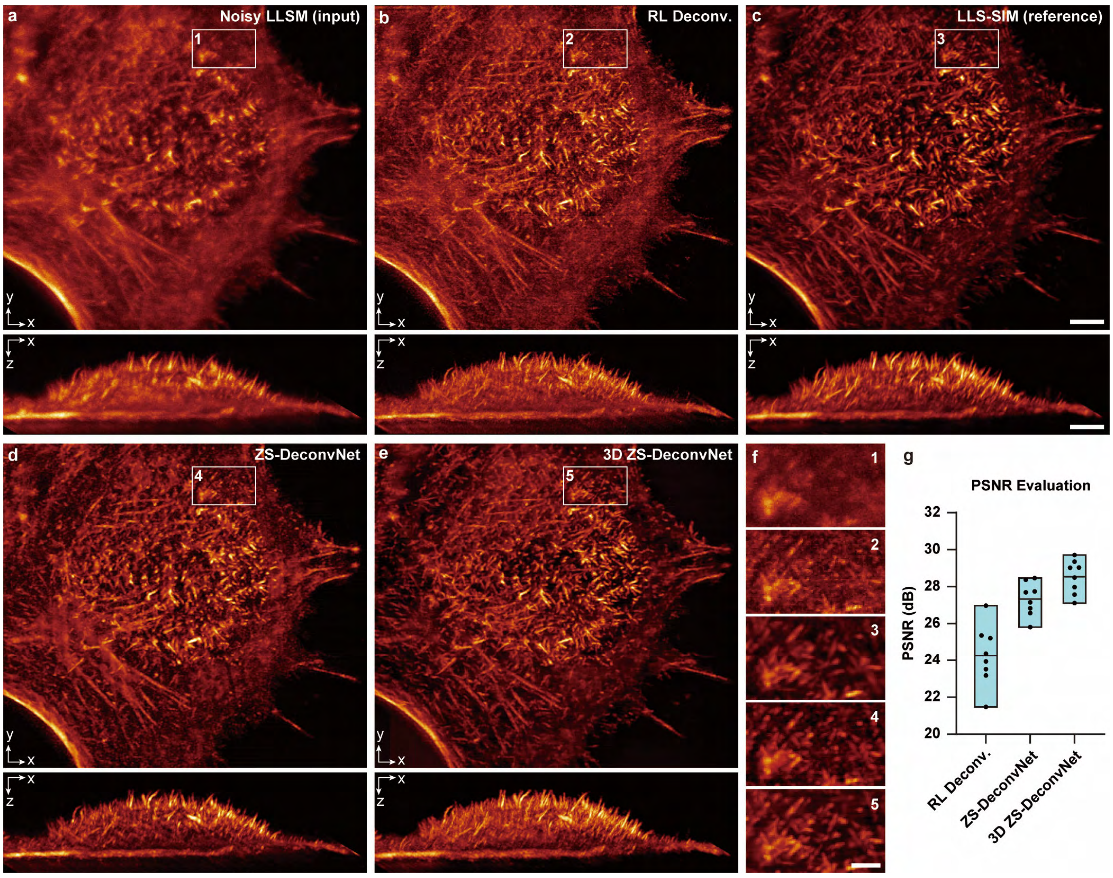

Supplementary Fig. 14 | Comparison of SR capability for 3D LLSM images with RL deconvolution, ZS-DeconvNet, and 3D ZS-DeconvNet. a-e, Representative maximum intensity projections (MIP) of F-actin in a COS-7 cell imaged by (a) LLSM (low excitation), (c) LLS-SIM (high excitation) and reconstructed with (b) RL deconvolution, (d) recorruption-based ZS-DeconvNet, and (e) spatially interleaved self-supervised 3D ZS-DeconvNet from the noisy LLSM image stack. Both *xy*-MIPs (upper) and *xz*-MIPs (lower) are provided. **f**, Magnified regions labelled in a-c with white boxes. **g**, Statistical comparison in terms of PSNR for RL deconvolution, ZS-DeconvNet, and 3D-DeconvNet (n=8). Source data are provided as a Source Data file. Scale bar, 8 μm (a-e), 2 μm (f).

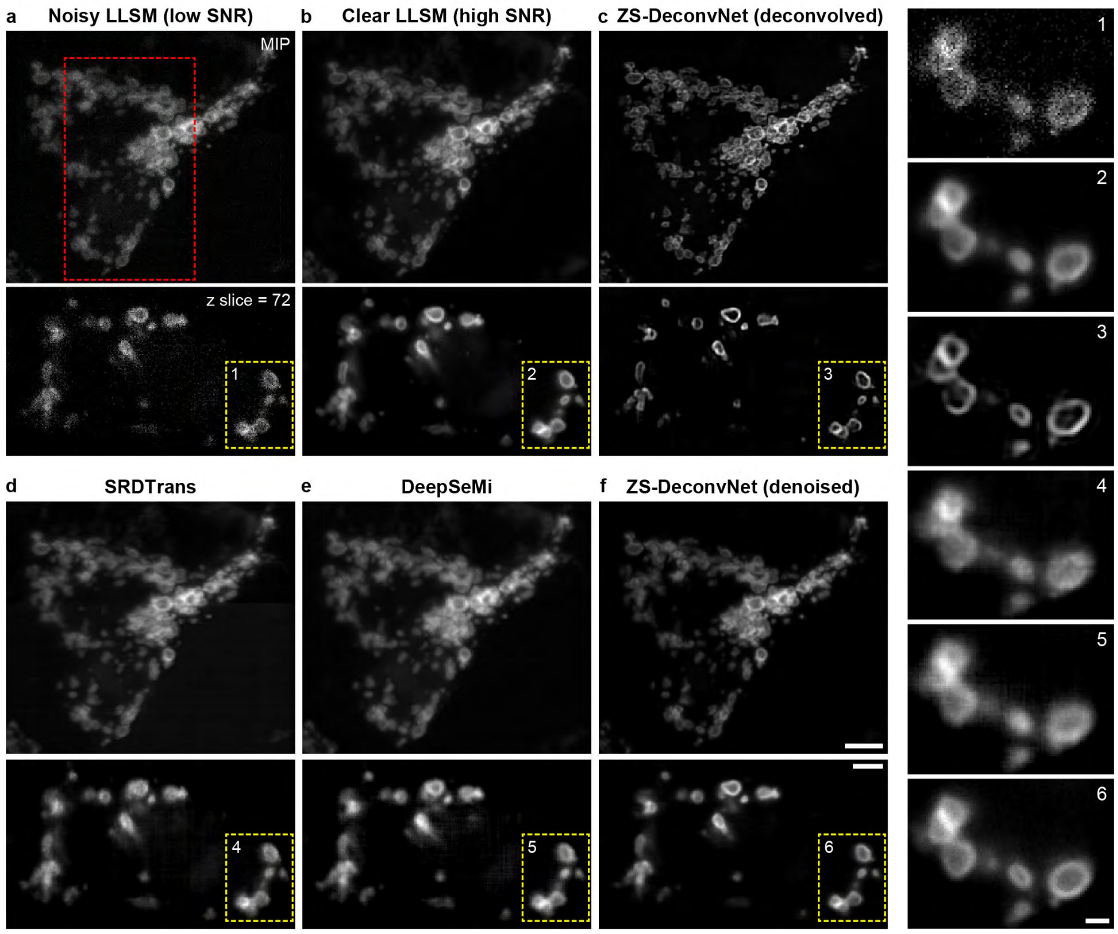

Supplementary Fig. 15 | Comparisons of ZS-DeconvNet with self-supervised denoising methods. Images of mitochondrial outer membrane captured or processed by LLSM (a for low SNR, b for high SNR), ZS-DeconvNet (c for deconvolved results, f for denoised results), SRDTrans[15] (d), and DeepSeMi[16] (e). Single-slice images of the region labelled by the red box and magnified regions labelled by yellow boxes are shown for better comparison. Scale bars, 5 μm (MIP), 3 μm (single slice), and 1 μm (magnified region).

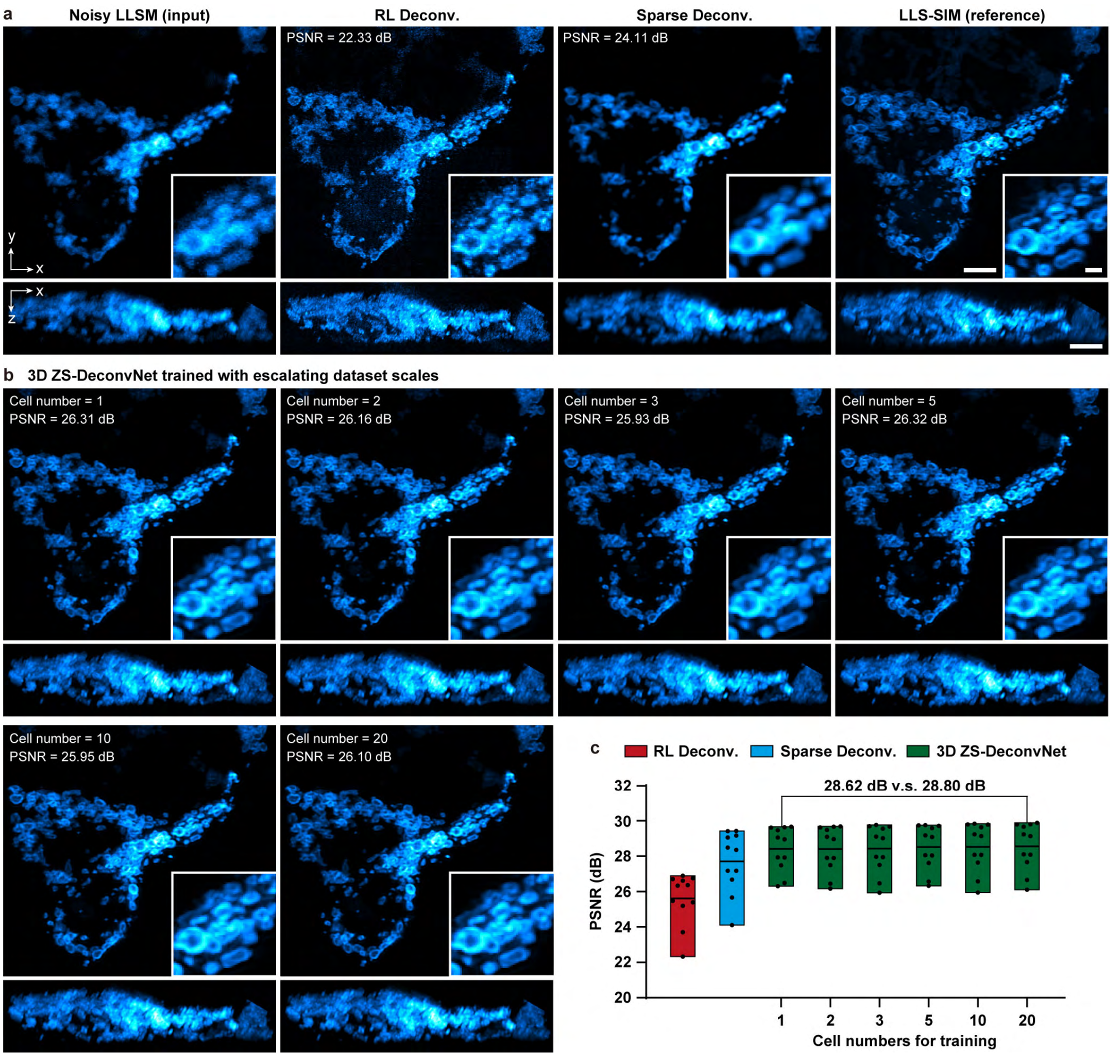

Supplementary Fig. 16 | Evaluation of 3D ZS-DeconvNet trained with different scales of datasets. **a**, Representative maximum intensity projections (MIP) of mitochondrial outer membrane in a 293T cell imaged by LLSM (low excitation), LLS-SIM (high excitation) and reconstructed with RL deconvolution, sparse deconvolution from the noisy LLSM image stack. **b**, Super-resolved MIP images generated by 3D ZS-DeconvNet models trained with escalating dataset scales of a single cell to 20 cells. **c**, Statistical comparisons in terms of PSNR for RL deconvolution (red), sparse deconvolution (blue), and 3D ZS-DeconvNet trained with different dataset scales (green) (n=11). Source data are provided as a Source Data file. Both *xy*-MIPs (upper) and *xz*-MIPs (lower) are shown and the PSNR values are labelled in the top left corner of each image. These results suggest the successful zero-shot implementation of 3D ZS-DeconvNet where the model was trained with data augmented from a single image stack, while yielding comparable performance to models trained with more data and outperforming conventional deconvolution algorithms. Scale bar, 5 μm (*xy*- and *xz*-MIPs of a and b), 1 μm (zoom-in regions of a and b).

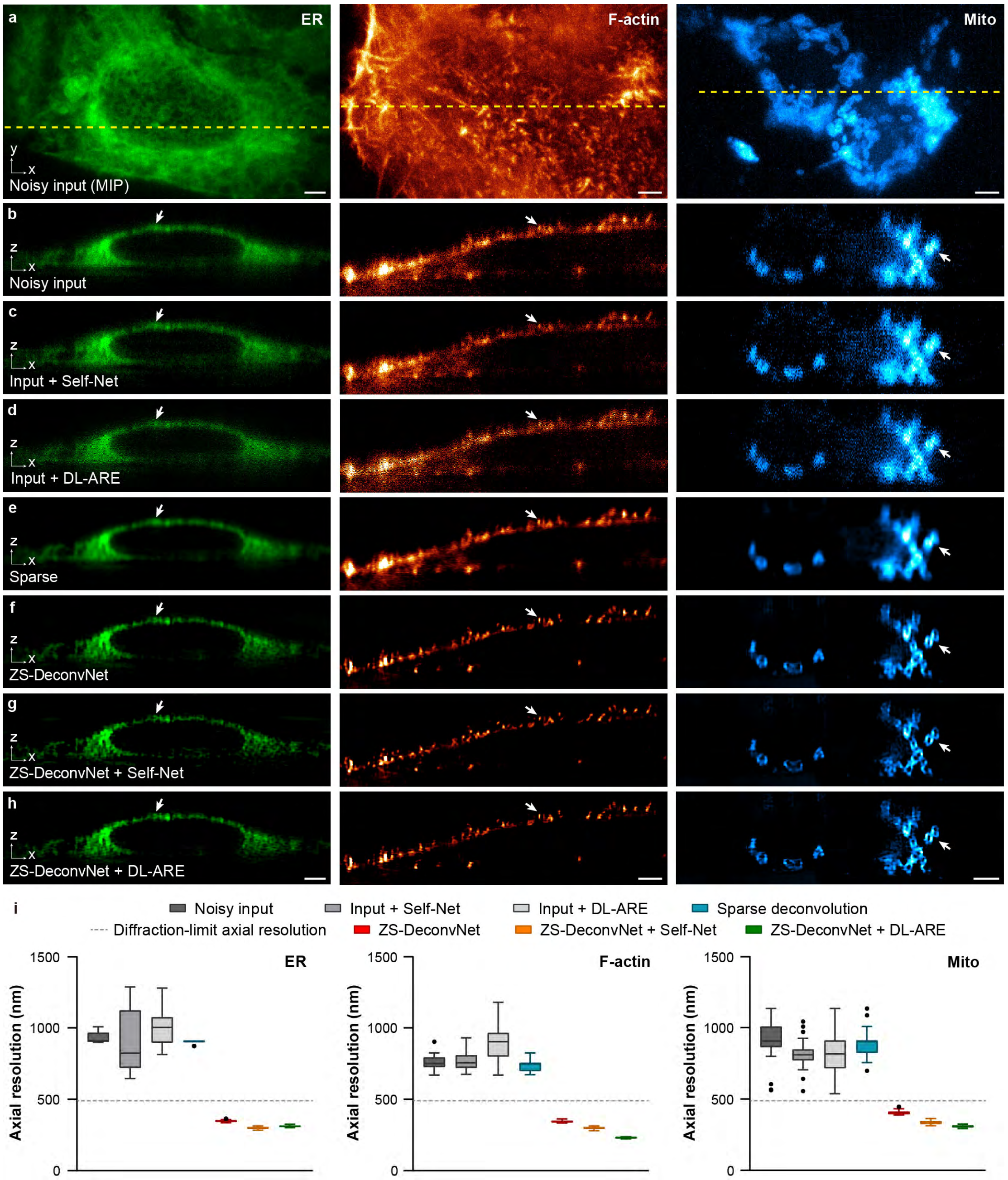

Supplementary Fig. 17 | Comparison of ZS-DeconvNet and other methods in axial resolution improvement. a, Representative noisy LLSM images (max intensity projection, MIP) of ER (left), F-actin (middle), and mitochondria (right). **b-h**, XZ-slices, of which the Y-positions are indicated by yellow dashed lines in a, from corresponding image stacks without processing (b) and processed by Self-Net[17] (c), DL-ARE[18] (d), sparse deconvolution (e), ZS-DeconvNet (f), ZS-DeconvNet + Self-Net (g), and ZS-DeconvNet + DL-ARE (h). **i**, Decorrelation axial resolution analysis for image stacks of ER (left), F-actin (middle), and Mito (right) processed by different methods (n=50 stacks for each biological structure). Center line, medians; limits, 75% and 25%; whiskers, the larger value between the largest data point and the 75th percentiles plus 1.5× the interquartile range (IQR), and the smaller value between the smallest data point and the 25th percentiles minus 1.5× the IQR; outliers, data points larger than the upper whisker or smaller than the lower whisker. Source data are provided as a Source Data file. Scale bar, 3 μm (a-h).

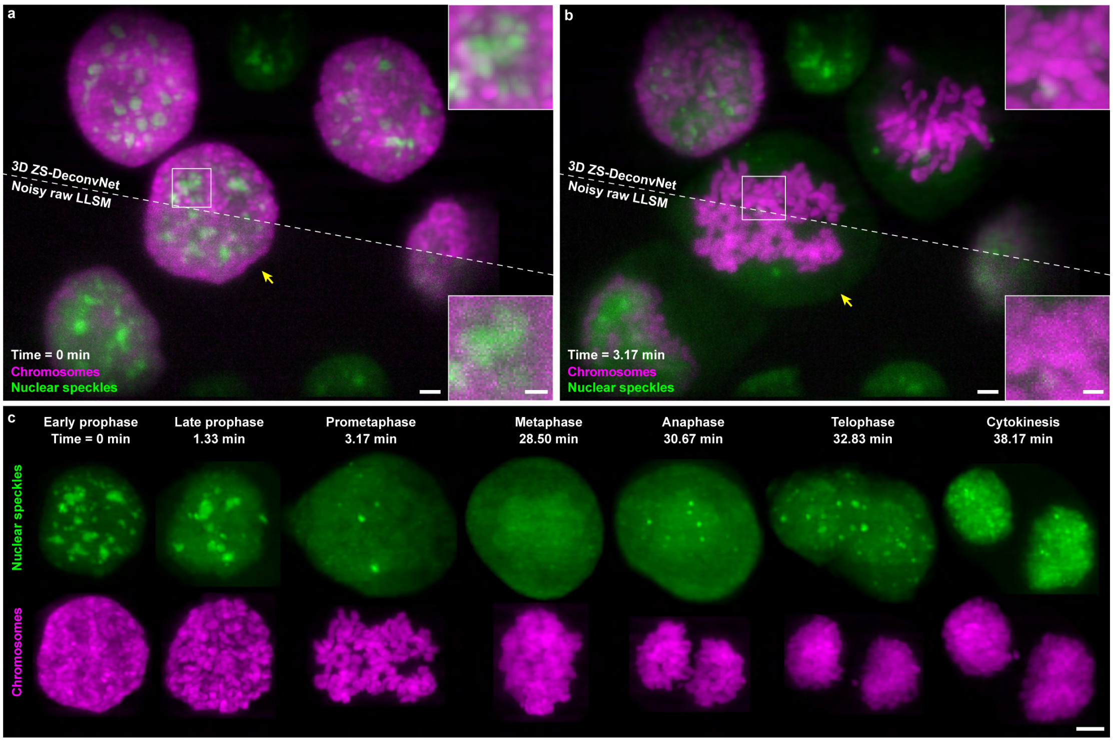

Supplementary Fig. 18 | Visualizing the behaviors of HeLa cells with mEmerald-SC35 labelled nuclear speckles and mCherry-H2B labelled chromosomes during cell mitosis via 3D ZS-DeconvNet enhanced LLSM. a, **b**, Two representative frames imaged by LLSM with low light-dose (bottom left) and enhanced by 3D ZS-DeconvNet (top right) in early prophase and prometaphase, respectively, during mitosis of several HeLa cells. **c**, Time-lapse 3D ZS-DeconvNet enhanced images of a mitotic HeLa cell indicated with yellow arrow in a and b, labelled by mEmerald-SC35 and mCherry-H2B, showing the disassemble and reassemble process of nuclear speckles during mitosis (Supplementary Video 5). Scale bar, 2.5 μm (a and b), 1 μm (zoom-in regions of a and b), 4 μm (c).

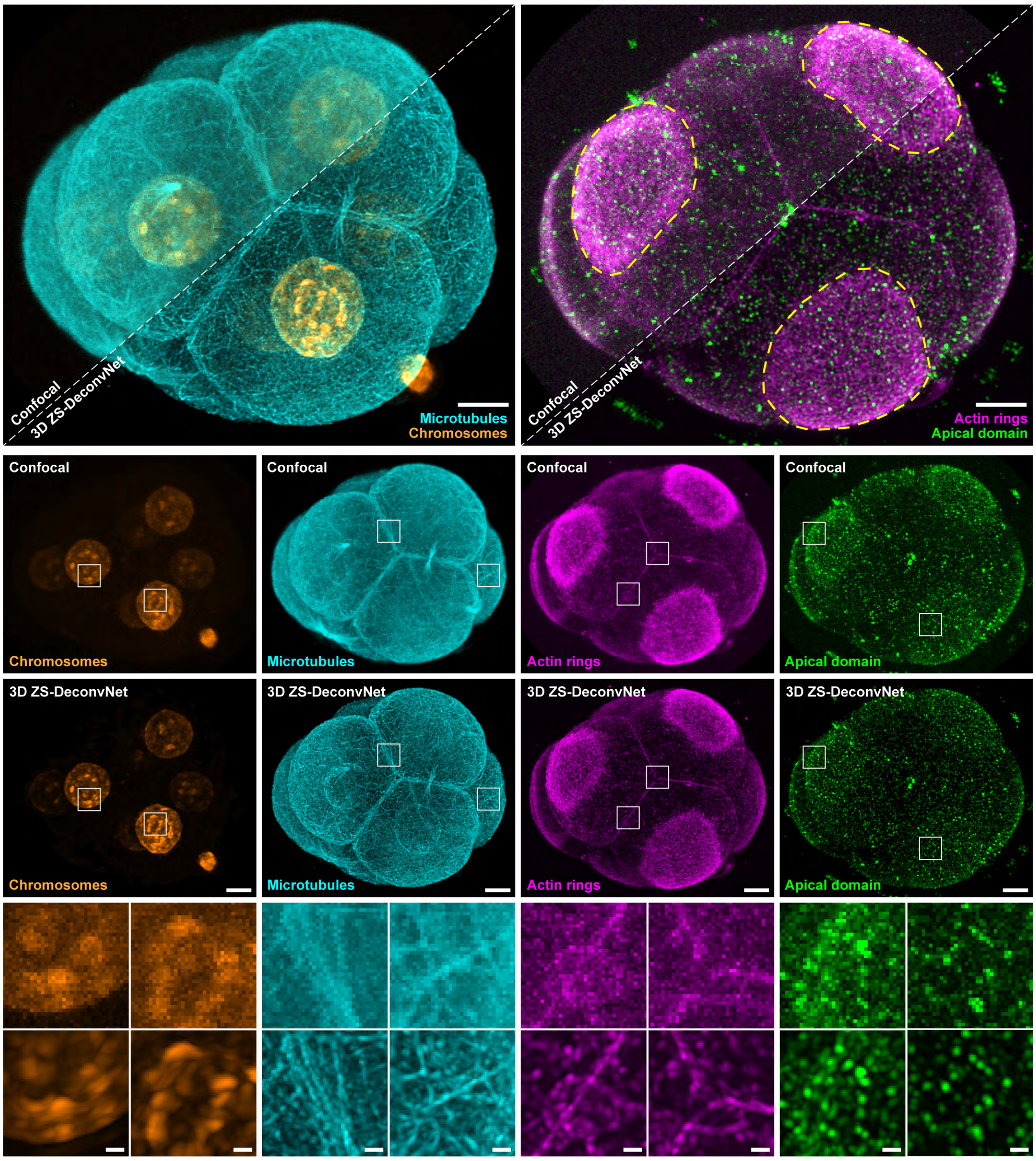

Supplementary Fig. 19 | Four-color super-resolution visualization of another early mouse embryo via 3D ZS-DeconvNet. 3D-rendering confocal images of early mouse embryo immunostained for microtubule bridges (cyan), chromosomes (orange), actin rings (magenta), and apical domain (green) before and after 3D ZS-DeconvNet enhancement. The 3D ZS-DeconvNet models trained with the input noisy data itself provide a dramatic improvement in both SNR, contrast, and resolution compared to the original confocal image stack. Scale bar, 8 µm (images of the entire embryo), 1 µm (zoom-in regions for each color channel).

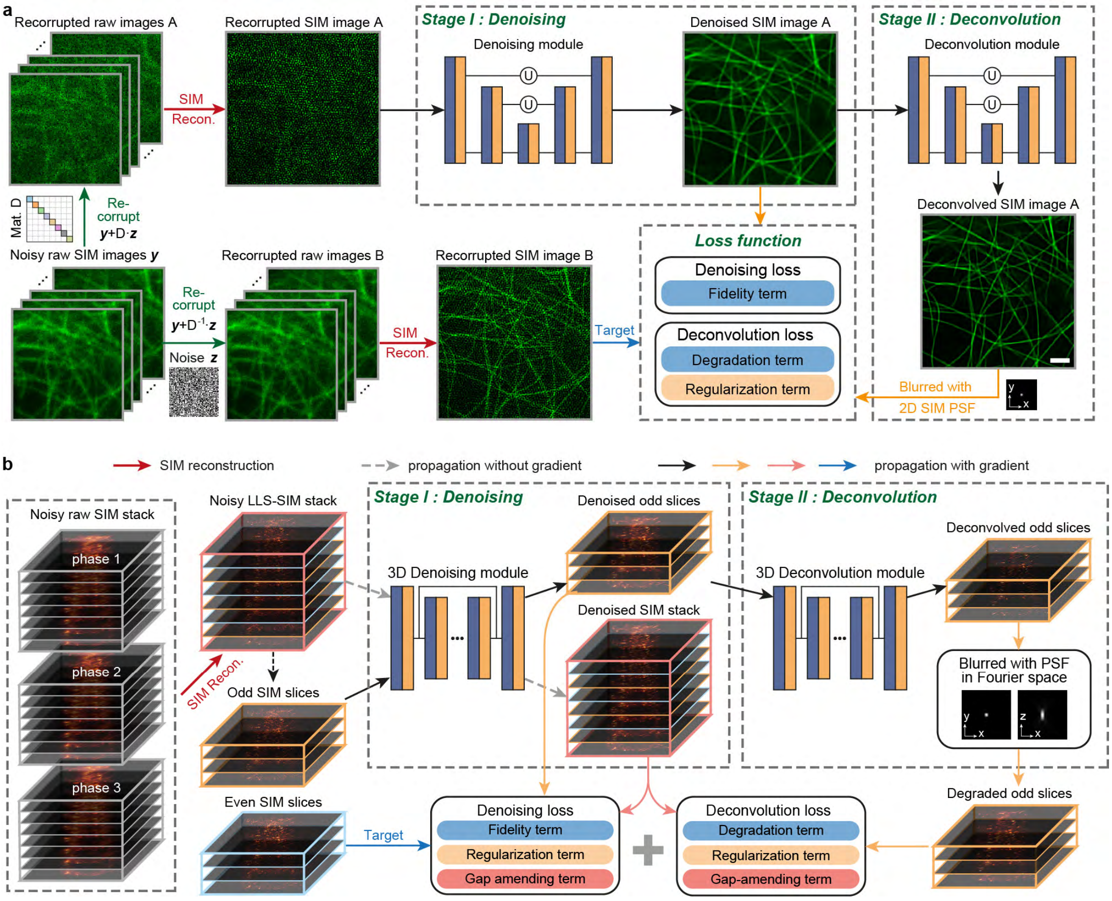

Supplementary Fig. 20 | Schematic training procedures of ZS-DeconvNet-SIM. **a**, Schematic of recorruption-based training procedure of ZS-DeconvNet for 2D SIM data, i.e., TIRF-SIM and GI-SIM. Scale bar, 1 μm. **b**, Schematic of spatially interleaved unsupervised training procedure of 3D ZS-DeconvNet for LLS-SIM data.

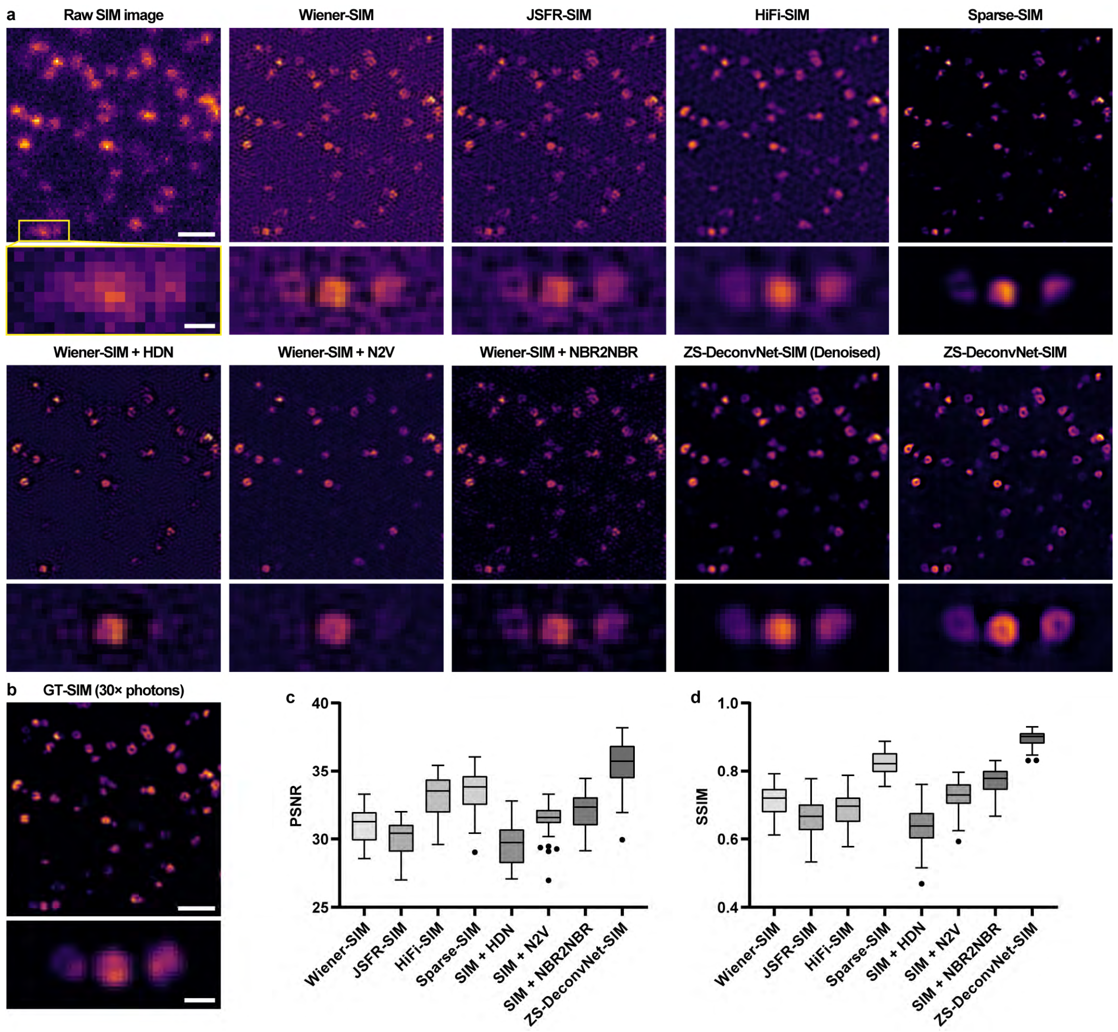

Supplementary Fig. 21 | Qualitative and Quantitative comparisons between ZS-DeconvNet-SIM and other state-of-the-art SIM reconstruction and denoising methods. a, Representative raw SIM image and SR-SIM images generated via various SIM reconstruction and denoising algorithms, including Wiener-SIM[3], JSFR-SIM[19], HiFi-SIM[20], Sparse-SIM[21], Hierarchical DivNoising (HDN)[22], Noise2Void (N2V)[23], Neighbor2Neighbor (NBR2NBR)[24], and ZS-DeconvNet-SIM. **b**, GT-SIM image of the same region in (a) for reference. **c,d**, Statistical comparison of different methods in terms of PSNR and SSIM (n=40). Center line, medians; limits, 75% and 25%; whiskers, the larger value between the largest data point and the 75th percentiles plus 1.5× the interquartile range (IQR), and the smaller value between the smallest data point and the 25th percentiles minus 1.5× the IQR; outliers, data points larger than the upper whisker or smaller than the lower whisker. Source data are provided as a Source Data file. Scale bar, 1 μm (a and b), 0.2 μm (zoom-in region of a and b).

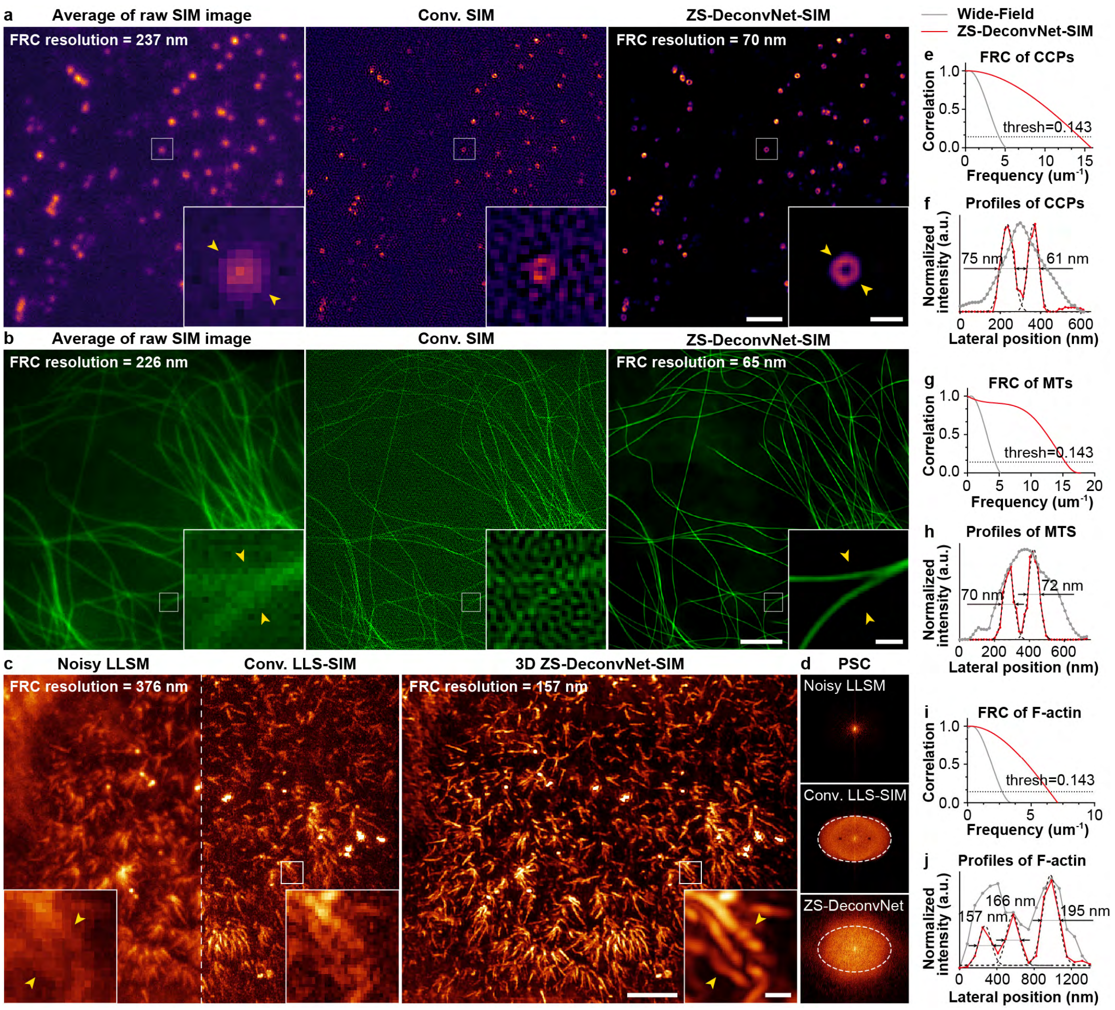

Supplementary Fig. 22 | Resolution comparison between wide-field images, conventional SIM images, and ZS-DeconvNet enhanced SIM images across multiple SIM modalities. a, b, Representative SR images of (a) clathrin coated pits (CCPs) and (b) microtubules (MTs) acquired with the (a) TIRF-SIM and (b) GI-SIM mode, respectively, of our Multi-SIM system and reconstructed with the conventional SIM algorithm and ZS-DeconvNet. **c, d**, Representative (c) SR images and (d) corresponding power spectrum coverages (PSC) of F-actin acquired with the LLS-SIM system and reconstructed via the conventional LLS-SIM algorithm and 3D ZS-DeconvNet. The envelopes of the LLS-SIM image are labelled with white dashed ellipse in d, suggesting an isotropic super-resolution capability of 3D-DeconvNet-SIM. **e-j**, Fourier ring correlation (FRC) curves (e, g, i) and intensity profile plots along the lines indicated by the two yellow arrowheads in a–c (f, h, j) for wide-field images (gray) and ZS-DeconvNet-SIM images (red) of CCPs (e, f), MTs (g, h), and F-actin (i, j). In these cases, the hollow structures of CCPs and adjacent filaments of microtubules and F-actin were clearly resolved by ZS-DeconvNet-SIM with a spatial resolution of ~70 nm for 2D-SIM and ~160 nm for LLS-SIM. By contrast, they were indistinguishable with either LLSM for its resolution limitation or conventional LLS-SIM for artifact contamination. Source data are provided as a Source Data file. Scale bar, 3 μm (a), 0.5 μm (zoom-in region of a), 3 μm (b), 0.3 μm (zoom-in region of b), 5 μm (c), 1 μm (zoom-in region of c).

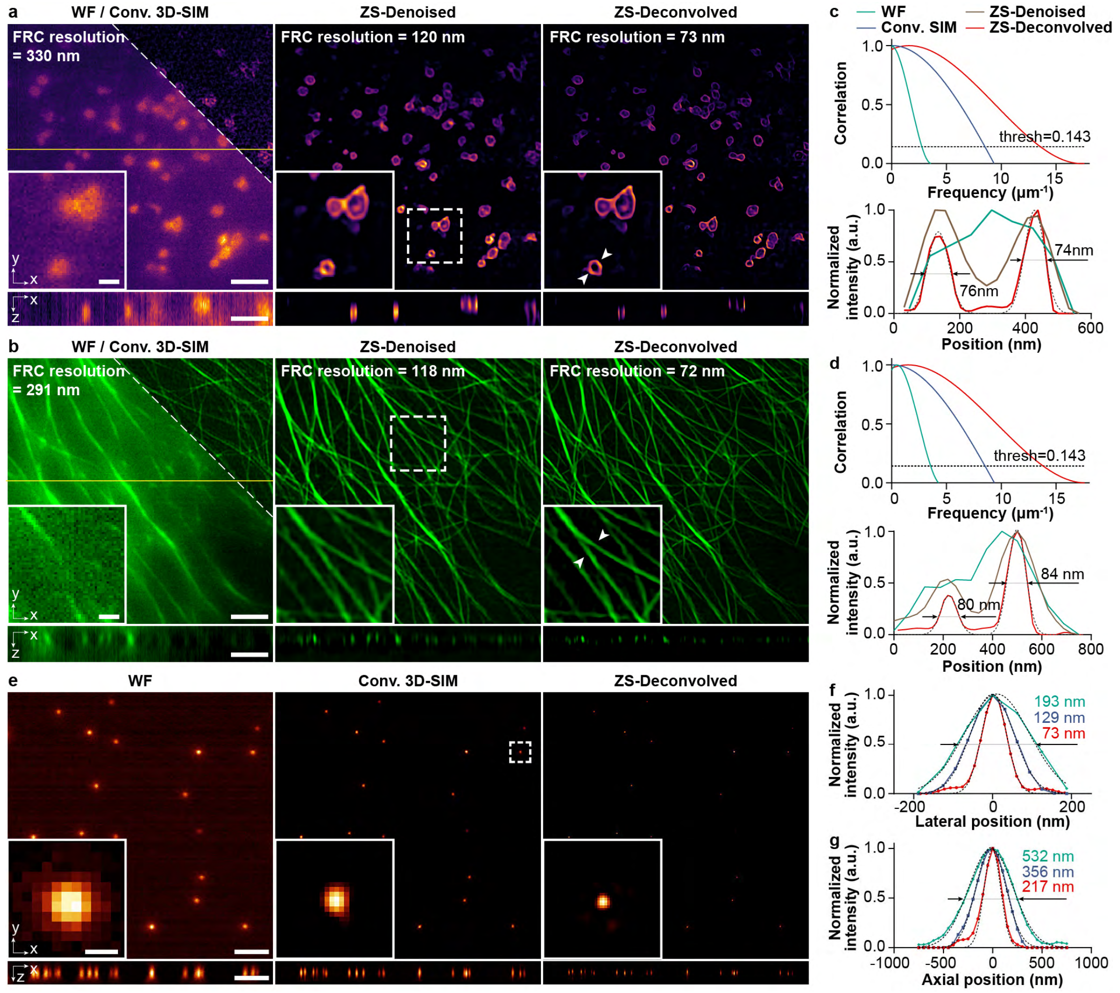

Supplementary Fig. 23 | Zero-shot denoising and resolution enhancement for 3D-SIM images. a, b, Representative max intensity projection (MIP) of wide-field (bottom left corner of the first column), conventional 3D-SIM (top right corner of the first column), zero-shot denoised (the second column) and deconvolved (the third column) images in terms of lysosomes (a) and microtubules (b) acquired with the 3D-SIM mode of our Multi-SIM system. FRC resolutions are labelled on each MIP image and the sectioned x-z views along the yellow lines are shown beneath the lateral MIPs. **c, d**, Fourier ring correlation (FRC) curves (upper panel) and intensity profile plots (lower panel) along the lines indicated by the two white arrowheads in a and b for wide-field (green), conventional 3D-SIM (blue), zero-shot denoised (brown) and deconvolved (red) images of lysosomes (c) and microtubules (d). **e**, Representative x-y (upper panel) and x-z (lower panel) MIPs of wide-field, conventional 3D-SIM, and ZS-DeconvNet-SIM images of experimentally acquired fluorescent beads. **f**, **g**, Averaged intensity profiles (n=12) in lateral (f) and axial (g) position of the fluorescent bead images. Source data are provided as a Source Data file. Scale bar, 2 μm (a, b), 0.5 μm (zoom-in region of a and b), 1 μm (e), 0.2 μm (zoom-in region of e). Gamma value, 0.8 for Conv. 3D-SIM, ZS-Denoised, and ZS-Deconvolved images of microtubules in b.

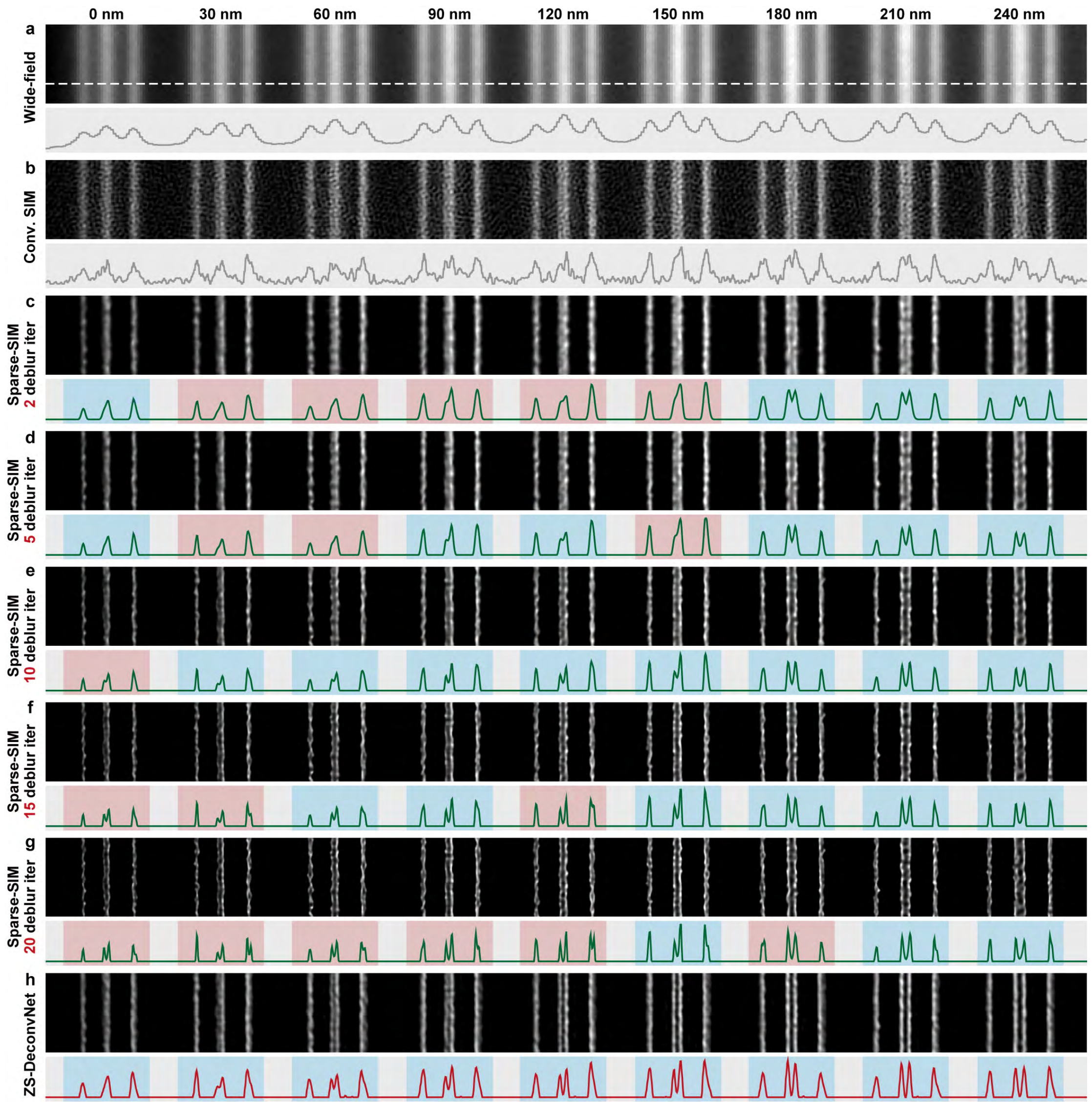

Supplementary Fig. 24 | Comparison between ZS-DeconvNet-SIM and sparse-SIM on Argo-SIM slides. A, **b**, Wide-filed (a) and conventional SIM (Conv. SIM) images (b) of Argo-SIM slides which consist of 9 pairs of dual lines, whose spacing gradually increases from 0 nm to 240 nm with a step of 30 nm. **c-g**, Sparse-SIM images reconstructed from the Conv. SIM image shown in b with different deblur iterations ranging from 2 to 20. **h**, SR image of the same content generated via ZS-DeconvNet-SIM model trained with noisy data only. The intensity profiles along the white dashed line labelled in a are shown in the lower panel for each method. We highlighted correct and false reconstructions including failing to resolve two parallel lines or wrongly distinguishing a single line apart, with bottom colors of blue and red on the line profiles of sparse-SIM and ZS-DeconvNet-SIM images, respectively. Of note, with a relatively large deblur iteration, i.e., larger than 10 (e-g), sparse-SIM generates several false-positive errors where a single line was wrongly resolved into two separated lines, while the proposed ZS-DeconvNet-SIM does not require any user-defined parameters and thereby yields SR images with higher fidelity.

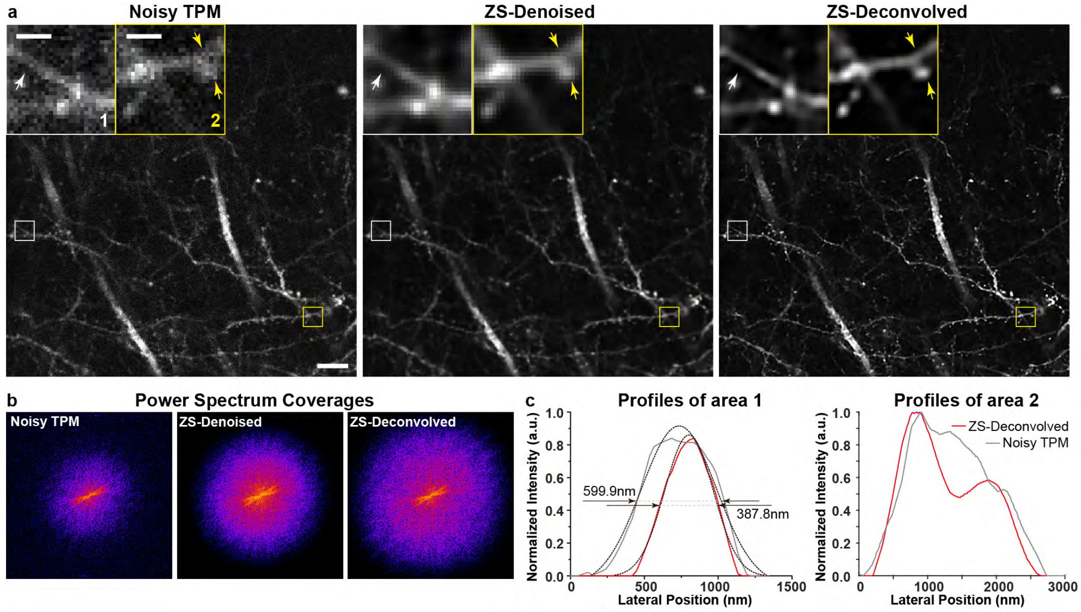

Supplementary Fig. 25 | Characterization of ZS-DeconvNet on two-photon microscopy (TPM). a, Representative noisy TPM image (left), denoised (middle) and deconvolved image (right) by ZS-DeconvNet. The ZS-DeconvNet model was trained with TPM image stack itself. **b**, Power spectrum coverages of the images shown in a. **c**, Intensity profiles along the lines indicated by the arrowheads in the two magnified regions of noisy TPM image (gray) and ZS-Deconvolved image (red) in a. Source data are provided as a Source Data file. Scale bar, 10 μm (a), 2 μm (zoom-in regions of a).

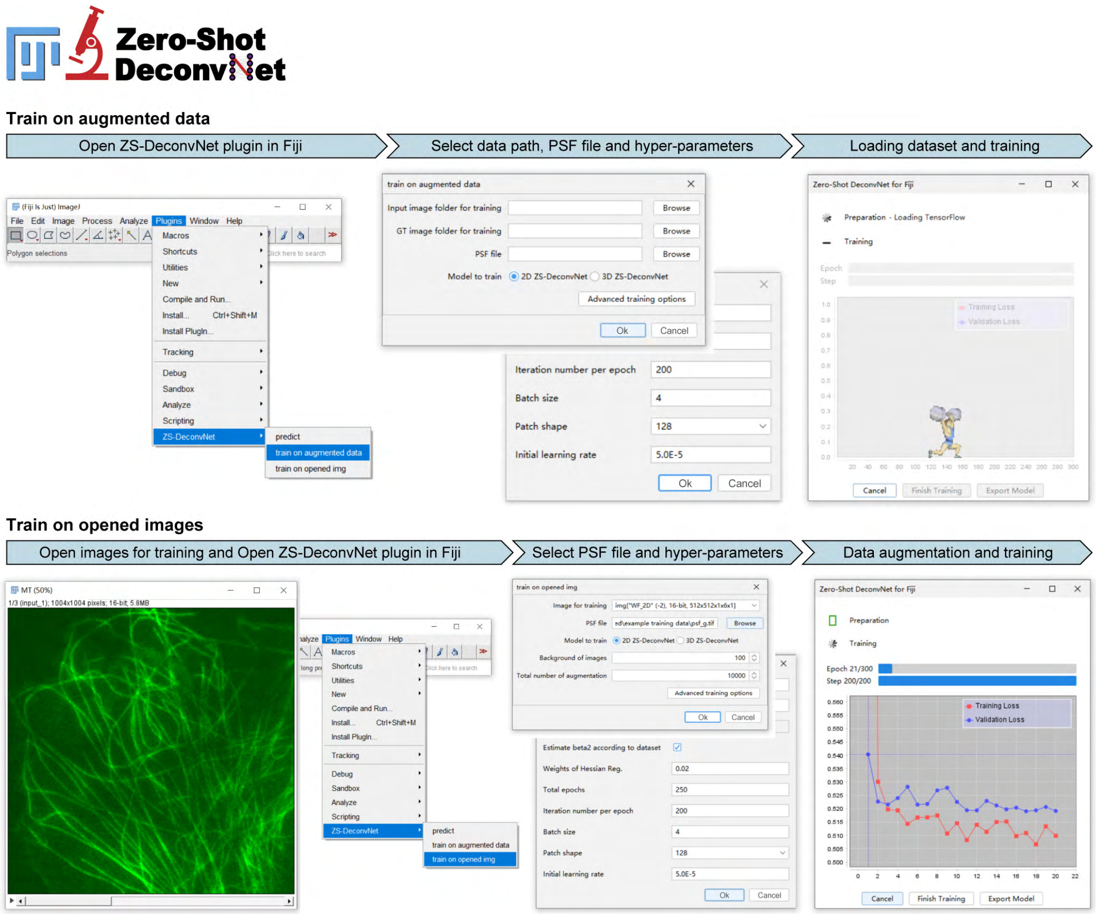

Supplementary Fig. 26 | Training workflow of the ZS-DeconvNet Fiji plugin. Two commands are provided for training a new ZS-DeconvNet model: *train on augmented data* (upper panel) and *train on opened images* (lower panel). The former command loads paired images which are augmented elsewhere, while the latter command directly takes the image (stack) opened in Fiji as original training data and automatically performs data augmentation before training. Generally, the training procedure via ZS-DeconvNet Fiji plugin includes four steps: (1) Start the plugin from Fiji's menu *Plugins > ZS-DeconvNet*; (2) Select the model type to be trained, PSF file, and training dataset (folder path or opened images); (3) Choose other hyperpamameters for training (see Supplementary Note 3 and the tutorial homepage of ZS-DeconvNet for detailed instructions about parameter setting); (4) Click *Ok* and begin training a new model. The model will be validated after each training epoch and the training/validation loss is plotted in real time. During or after the training procedure, the trained model weights can be exported via the command *Export Model* and then used for subsequent inference. Note that this figure is intended to show the training workflow of the ZS-DeconvNet Fiji plugin but not to give suggested parameters. Refer to Supplementary Notes 3, 4, or our tutorial homepage for more information about detailed implementation and parameter suggestions.

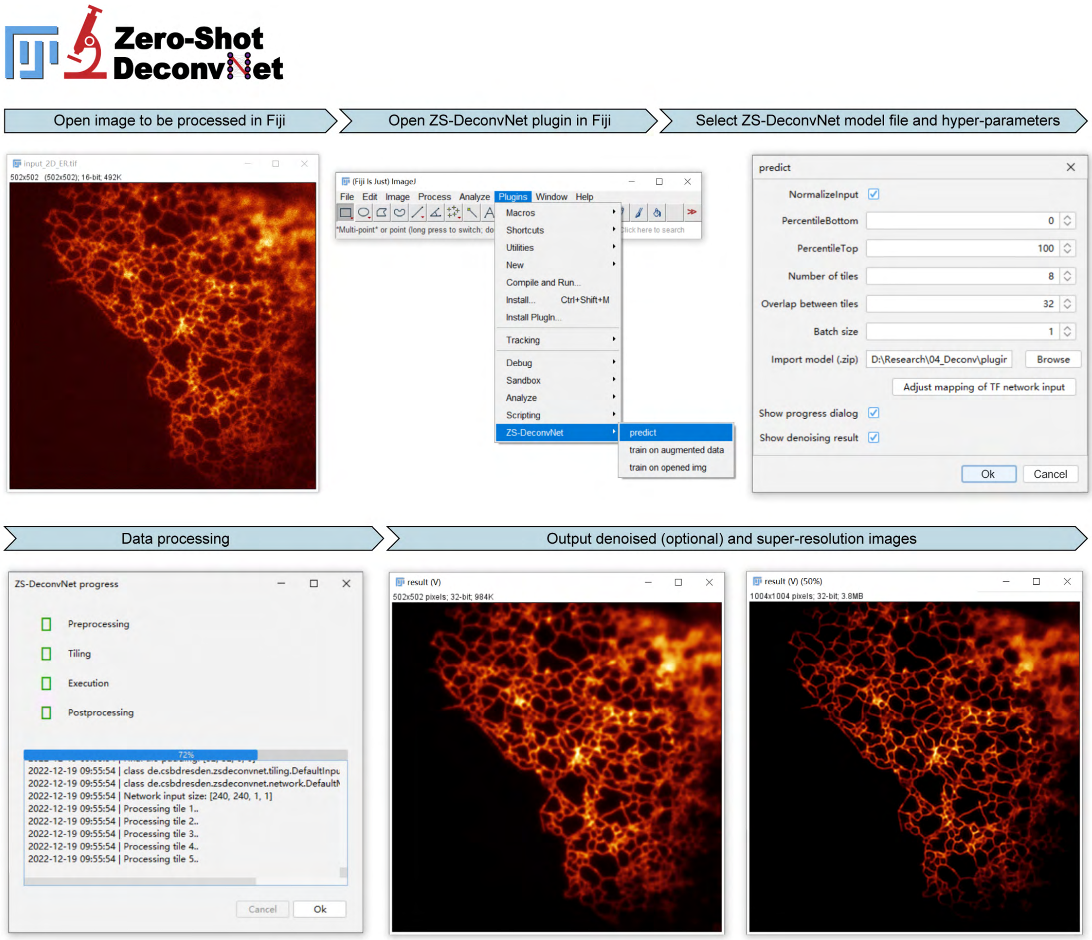

Supplementary Fig. 27 | Inference workflow of the ZS-DeconvNet Fiji plugin. The implementation of ZS-DeconvNet Fiji plugin typically includes the following five steps: (1) Open an image to be processed in Fiji; (2) Start our plugin from Fiji's menu *Plugins* > *ZS-DeconvNet* > *predict*; (3) Select an adequate ZS-DeconvNet model and set hyperparameters (see Supplementary Note 3 and 4 for detailed instructions about parameter setting); (4) Image processing with status shown in the message box; (5) The denoised image (optional) and the SR image are shown in separate Fiji windows. Note that this figure is intended to show the inference workflow of the ZS-DeconvNet Fiji plugin but not to give suggested parameters. Refer to Supplementary Notes 3, 4, or our tutorial homepage for more information about detailed implementation and parameter suggestions.

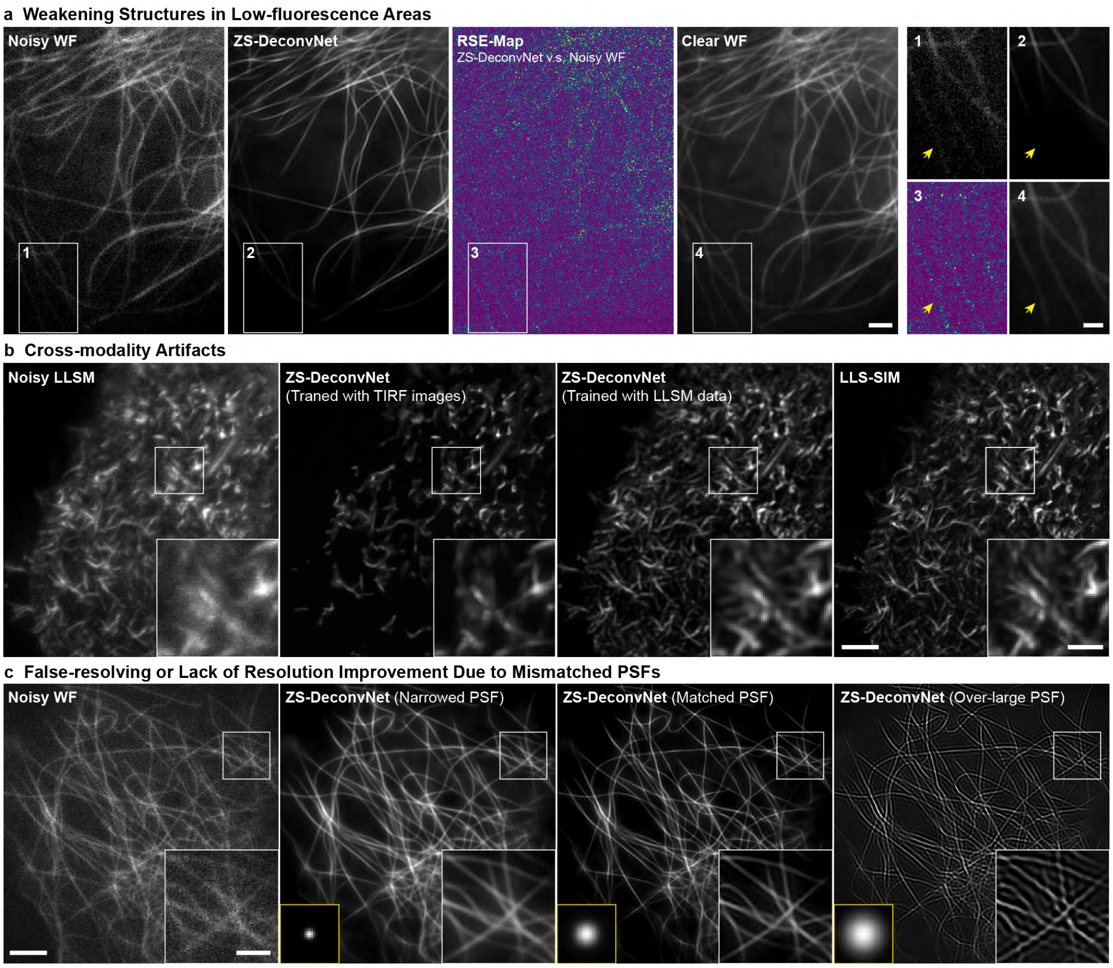

Supplementary Fig. 28 | Potential hallucinations generated by ZS-DeconvNet. a, Representative noisy WF image (first column), ZS-DeconvNet enhanced image (second column), resolution-scaled error (RSE) map (third column) calculated between the noisy WF image and ZS-DeconvNet image, and clear WF image for reference (fourth column). The yellow arrowheads in the magnified images point out an area where ZS-DeconvNet over-weakened the microtubule structures. From our experience, this kind of hallucinations usually happen in regions where the fluorescence intensity in the raw input image is too low, and could be identified to some extent by quality-check tools such as SQUIRREL analysis[25]. **b**, F-actin images (MIP) acquired by LLSM (first column), LLS-SIM (fourth column) and reconstructed by ZS-DeconvNet trained with unmatched TIRF images (second column) and noisy data itself (third column). These results indicate that there might be noticeable performance degradation when applied a trained ZS-DeconvNet model to data of different imaging modalities, e.g., with different pixel sizes, theoretical resolution, background, etc. **c**, Noisy WF images of microtubules (first column) and super-resolved images generated with ZS-DeconvNet models trained with over-narrowed PSF (second column), matched PSF (third column), and over-large PSF (fourth column). These images demonstrate that ZS-DeconvNet models should be trained using matched PSFs with the input images, otherwise the improper training with mismatched PSFs might result in lack of resolution improvement or ringing artifacts. Scale bar, 2 μm (a), 1 μm (zoom-in regions of a), 4 μm (b, c), 1.5 μm (zoom-in regions of b and c).

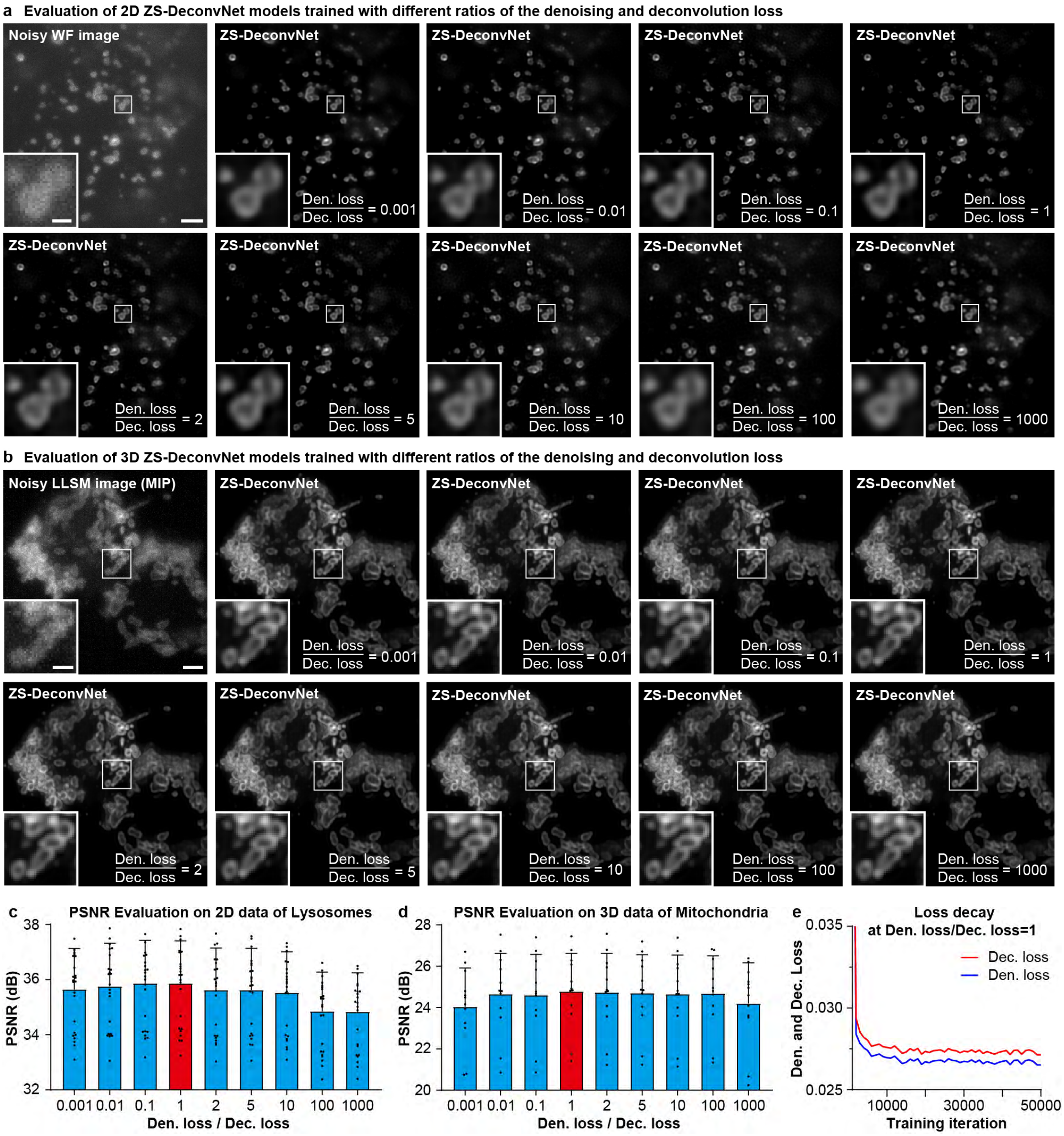

Supplementary Fig. 29 | Evaluation of ZS-DeconvNet models trained with different μ. a, Representative processed images of lysosomes with 2D ZS-DeconvNet models trained with different μ where the ratio of denoised loss and deconvolved loss ranges from 0.001 to 1000. **b**, Representative processed images (max intensity projection) of mitochondria with 3D ZS-DeconvNet models trained with different μ where the ratio of the denoised loss and deconvolved loss ranges from 0.001 to 1000. **c**,**d**, Evaluation in terms of PSNR of 2D ZS-DeconvNet (c, n=24) and 3D ZS-DeconvNet (d, n=12) models trained with different μ. **e**, Decay curves of the denoising and deconvolution loss during the training process of a 2D ZS-DeconvNet model with $\mu = 0.5$. Source data are provided as a Source Data file. Scale bar, 2.5 µm (a, b), 0.5 µm (zoom-in regions of a), 1 µm (zoom-in regions of b).

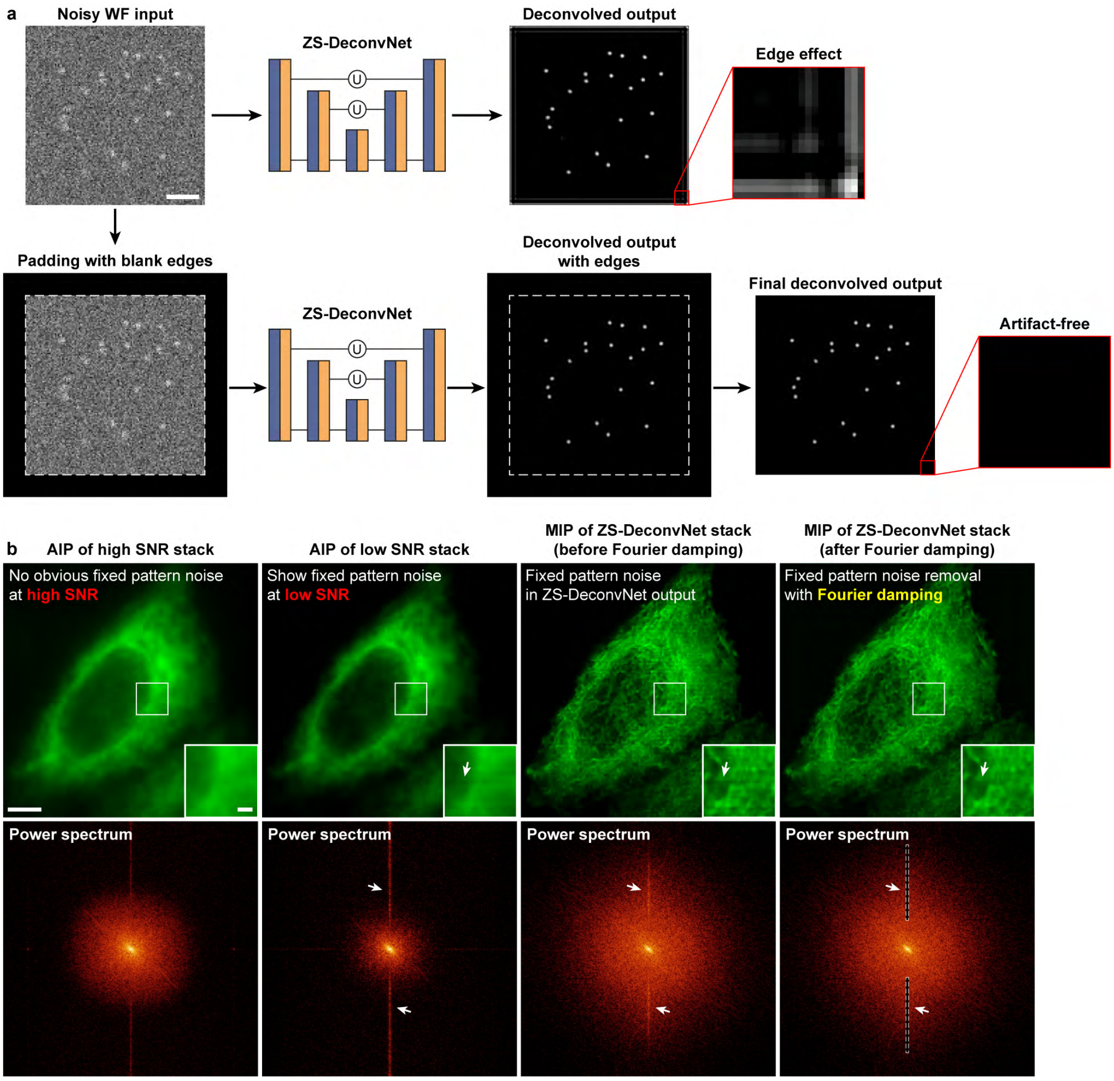

Supplementary Fig. 30 | Artifact elimination for ZS-DeconvNet. a, A representative case showing that the deconvolution-induced edge artifact (upper row) and the artifact elimination by padding blank edges surrounding the input image and cutting them off after network processing (lower row). **b**, Representative average intensity projections (AIPs) of ER acquired by LLSM at high (first column) and low (second column) SNR conditions, and corresponding MIPs of 3D ZS-DeconvNet enhanced image stacks before (third column) and after (fourth column) the Fourier damping operation. The power spectrums of each image are shown below. The emergence and removal of fixed pattern noises is highlighted by white arrows in spatial and Fourier domains and the Fourier apodization masks are labelled with white dashed rectangles. Scale bar, 1.5 µm (a), 5 µm (b), 1 µm (zoom-in region of b).

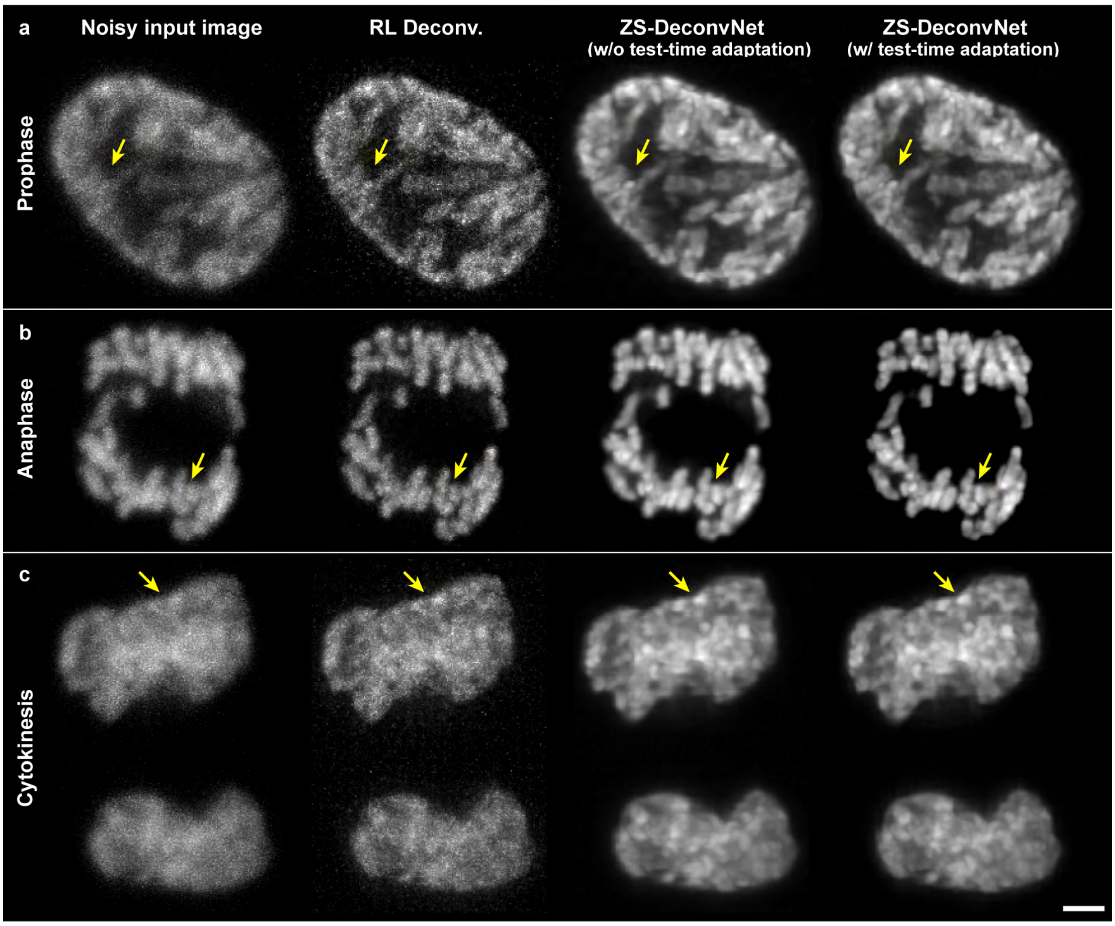

Supplementary Fig. 31 | Test-time adaptation for ZS-DeconvNet. Representative chromosome images (max intensity projection) of prophase (**a**), anaphase (**b**), and cytokinesis (**c**) selected from the time-lapse data of cell mitosis corresponding to Fig. 3e, f and Supplementary Video 5, which were imaged and processed by LLSM (the first column), RL deconvolution (the second column), 3D ZS-DeconvNet without (the third column) / with (the fourth column) test-time adaptation. Scale bar, 3 μm.

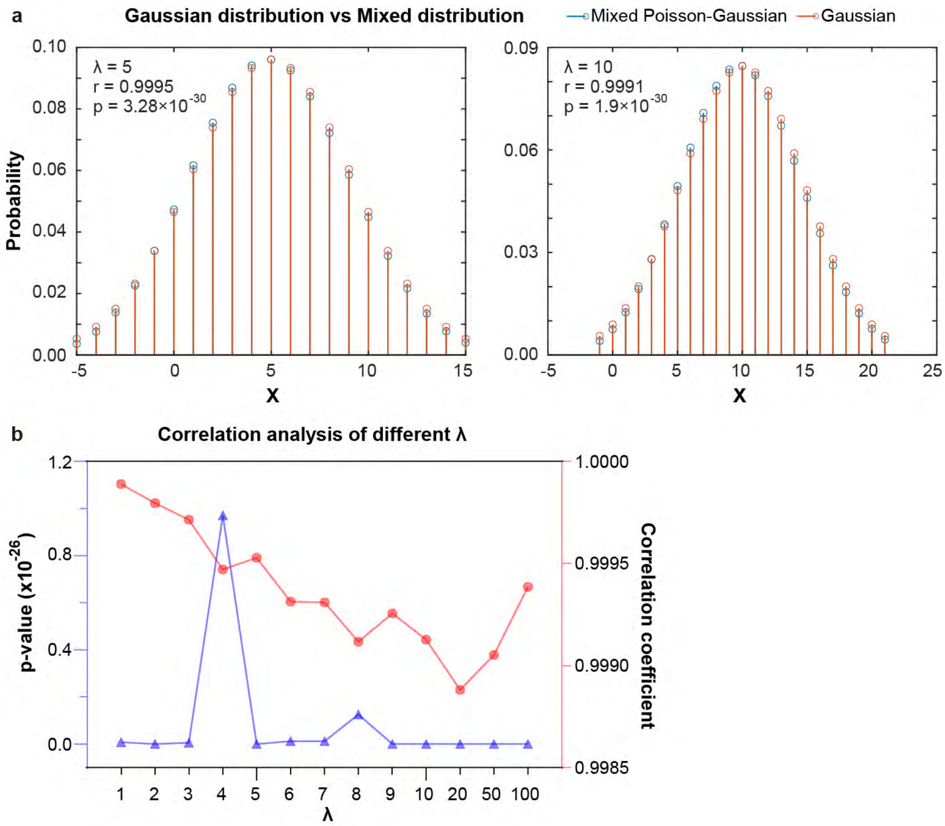

Supplementary Fig. 32 | Gaussian approximation to mixed Poisson-Gaussian distribution. a, Probability distribution of the Gaussian distribution $\mathcal{N}(\lambda, \lambda + \sigma_G^2)$ and mixed Poisson-Gaussian distribution with $\lambda = 5$ (left panel) and $\lambda = 10$ (right panel), respectively. **b**, Curves of the Pearson correlation and corresponding p-values between the approximated Gaussian distribution and the mixed Poisson-Gaussian distribution across different λ ranging from 1 to 100. These statistical results demonstrate a strong consistency between the Gaussian distribution $\mathcal{N}(\lambda, \lambda + \sigma_G^2)$ and the mixed Poisson-Gaussian distribution in fluorescence imaging. Source data are provided as a Source Data file.

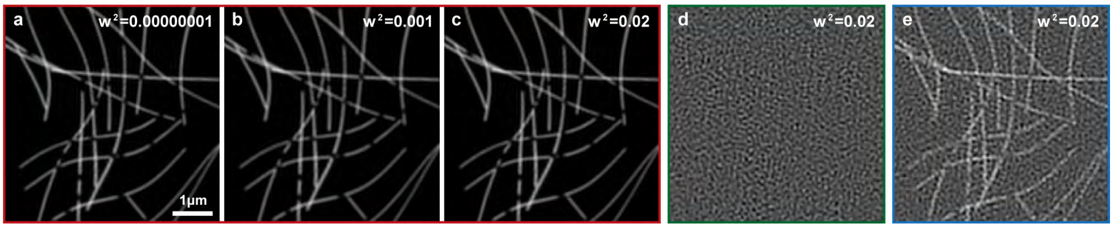

$$\frac{\sum_{d,m} O^*(\boldsymbol{k}+m\boldsymbol{p}_d) I'_{d,m}(\boldsymbol{k}+m\boldsymbol{p}_d)}{\sum_{d,m} |O(\boldsymbol{k}+m\boldsymbol{p}_d)|^2+\omega^2} A(\boldsymbol{k}) - \frac{\sum_{d,m} O^*(\boldsymbol{k}+m\boldsymbol{p}_d) N'_{d,m}(\boldsymbol{k}+m\boldsymbol{p}_d)}{\sum_{d,m} |O(\boldsymbol{k}+m\boldsymbol{p}_d)|^2+\omega^2} A(\boldsymbol{k}) = \hat{Y}(\boldsymbol{k})$$

Supplementary Fig. 33 | Different reconstruction components of a noisy SR-SIM image. a-c, Noise-free SR-SIM reconstructions filtered by a wiener parameter ω^2 of 1×10^{-8} (left), 1×10^{-3} (middle), and 0.02 (right), respectively. **d**, Noise-induced reconstruction artifacts of the noisy SR-SIM image $\hat{\mathbf{Y}}(\mathbf{k})$. **e**, Noisy SR-SIM image $\hat{\mathbf{Y}}(\mathbf{k})$ containing both the noise-free reconstructed components (c) and the noise-induced reconstruction artifacts (d).

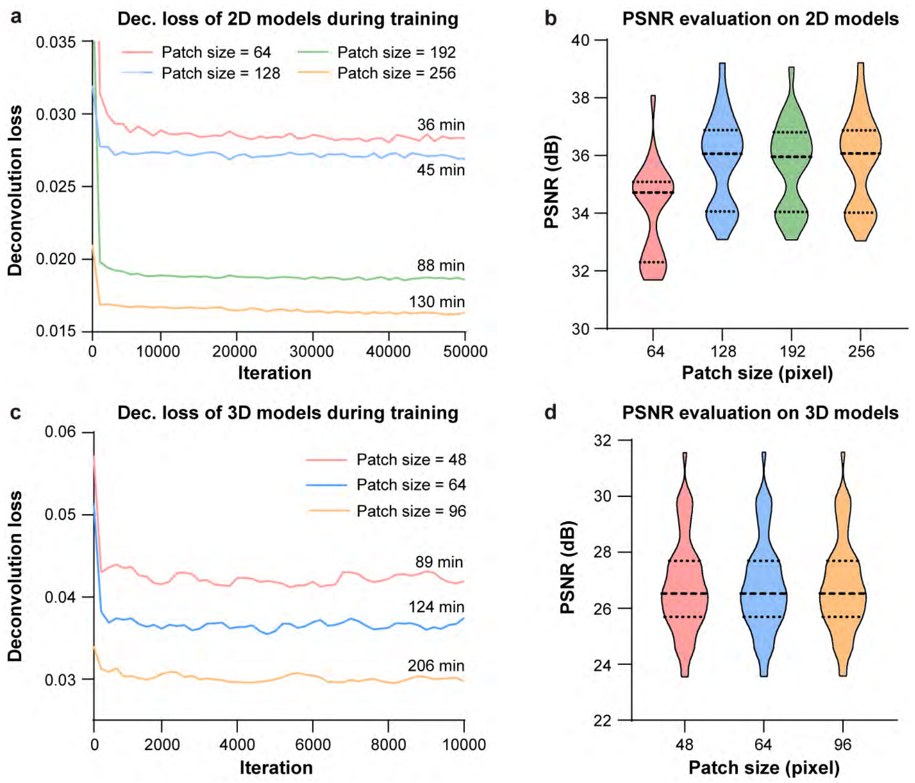

Supplementary Fig. 34 | Evaluation of 2D and 3D ZS-DeconvNet models trained with different patch sizes. a,b, Deconvolution loss curves and PSNR evaluations of four 2D ZS-DeconvNet models trained with four different patch sizes of 64×64, 128×128, 192×192, and 256×256 pixels, respectively (n=24 for PSNR evaluations). The training and testing were performed on the dataset of experimentally acquired lysosome images. **c,d**, Deconvolution loss curves and PSNR evaluations of three 3D ZS-DeconvNet models trained with different patch sizes of $48 \times 48 \times 13$, $64 \times 64 \times 13$, and $96 \times 96 \times 13$ voxels, respectively (n=60 for PSNR evaluations). The training and testing were performed on the dataset of experimentally acquired mitochondria images. The total training time of each model is labelled besides the loss curves in a and c. Source data are provided as a Source Data file.

Supplementary Tables

Supplementary Table 1. Implementation details of ZS-DeconvNet

	Imaging method	Network model type	Initial learning rate	Training patch size	Training batch size	Total training iterations	Training time (hours)
Fig. 1a, c<br>Supplementary Fig. 6b<br>Supplementary Fig. 8c<br>Supplementary Fig. 10a<br>Supplementary Fig. 11a, b<br>Supplementary Fig. 28a-c<br>Supplementary Fig. 29a	TIRF	ZS-DeconvNet	5×10^{-5}	128×128	4	80,000	1.6
Fig. 2a-d<br>Supplementary Fig. 11e<br>Supplementary Videos 2, 3	TIRF	ZS-DeconvNet	5×10^{-5}	128×128	4	50,000	1
Fig. 2e, f, i<br>Supplementary Fig. 13<br>Supplementary Video 4	TIRF	ZS-DeconvNet	5×10^{-5}	128×128	4	50,000	1
Fig. 3c<br>Supplementary Fig. 10b<br>Supplementary Fig. 11f<br>Supplementary Fig. 14<br>Supplementary Fig. 15f<br>Supplementary Fig. 16<br>Supplementary Fig. 17<br>Supplementary Fig. 28b<br>Supplementary Fig. 29b<br>Supplementary Fig. 30b	LLSM	3D ZS-DeconvNet	1×10^{-4}	$64 \times 64 \times 13$	3	10,000	2
Fig. 3e, f<br>Supplementary Fig. 31<br>Supplementary Video 5	LLSM	3D ZS-DeconvNet	1×10^{-4}	$64 \times 64 \times 13$	3	10,000	2

Fig. 4a-d<br>Supplementary Fig. 19<br>Supplementary Video 7	Confocal microscopy	3D ZS-DeconvNet	1×10^{-4}	$48 \times 48 \times 5$	3	10,000	1.1
Fig. 4e-h<br>Supplementary Video 8	3D wide-field microscopy	3D ZS-DeconvNet	1×10^{-4}	$64 \times 64 \times 13$	3	10,000	2
Fig. 5b, c<br>Supplementary Fig. 21<br>Supplementary Fig. 22a, b	TIRF-SIM<br>GI-SIM	ZS-DeconvNet-SIM	5×10^{-5}	128×128	4	50,000	1
Fig. 5d, e<br>Supplementary Fig. 22c	LLS-SIM	3D ZS-DeconvNet-SIM	1×10^{-4}	$64 \times 64 \times 13$	3	10,000	2
Supplementary Fig. 7a	TIRF	ZS-DeconvNet	5×10^{-5}	128×128	4	50,000	1
Supplementary Fig. 7d	TIRF	ZS-DeconvNet	5×10^{-5}	128×128	4	50,000	1
Supplementary Fig. 18<br>Supplementary Video 6	LLSM	3D ZS-DeconvNet	1×10^{-4}	$64 \times 64 \times 13$	3	10,000	2
Supplementary Fig. 23	3D-SIM	ZS-DeconvNet-SIM	1×10^{-4}	$64 \times 64 \times 7$	3	10,000	1.3
Supplementary Fig. 24	TIRF-SIM	ZS-DeconvNet-SIM	5×10^{-5}	128×128	4	50,000	1
Supplementary Fig. 25	TPM	3D ZS-DeconvNet	1×10^{-4}	$64 \times 64 \times 13$	3	10,000	2

Supplementary Table 2. Computation time of ZS-DeconvNet and other deconvolution methods

	Imaging method	Image size (pixels/voxels)	ZS-DeconvNet		Sparse deconvolution		RL deconvolution
			Network model type ①	Computation time (sec) ②	Tunable parameters ③	Computation time (sec)	Computation time (iterations) (sec)
Fig. 1c	TIRF	Lyso: 512 × 512 × 1	ZS-DeconvNet (×2)	0.05	(0, 30, 0.1, 15, 5)	9.24	0.13 (10)
		MTs: 512 × 512 × 1			(1, 25, 0.5, 300, 10)	23.78	
Fig. 2a-d Supplementary Videos 2, 3	TIRF	1024 × 1024 × 1	ZS-DeconvNet (×2)	0.23	\	\	0.59 (10)
Fig. 2e, f, i Supplementary Fig. 13 Supplementary Video 4	TIRF	488: 768 × 768 × 1	ZS-DeconvNet (×2)	0.13	\	\	0.23 (5)
		560: 768 × 768 × 1					0.29 (10)
Fig. 3c	LLSM	Mito: 763 × 512 × 151	3D ZS-DeconvNet	46.01	(0, 60, 0.1, 100, 15)	77.14	10.48 (10)
		F-actin: 761 × 512 × 301		51.25	(0, 150, 0.5, 100, 5)	106.29	20.83 (10)
		ER: 761 × 512 × 301		51.25	(0, 60, 0.5, 100, 10)	121.34	20.50 (10)
Fig. 3e, f Supplementary Video 5	LLSM	488: 382 × 382 × 151	3D ZS-DeconvNet	14.01	(0, 25, 0.5, 100, 10)	29.43	\
		560: 382 × 382 × 151			(0, 60, 0.5, 100, 5)	26.50	
		642: 382 × 382 × 151			(0, 25, 0.5, 100, 5)	26.67	
Fig. 4a-d Supplementary Fig. 19 Supplementary Video 7	Confocal microscopy	375: 526 × 526 × 147	3D ZS-DeconvNet (×2)	34.85	(1, 35, 0.1, 200, 50)	570.43	69.94 (10)
		488: 526 × 526 × 147			(1, 45, 0.1, 200, 50)	580.17	
		560: 526 × 526 × 147			(1, 25, 0.1, 200, 50)	553.38	
		642: 526 × 526 × 147			(1, 25, 0.1, 200, 50)	536.57	
Fig. 4e-h Supplementary Video 8	3D WF microscopy	1024 × 1024 × 94	3D ZS-DeconvNet	42.82	\	\	\
Fig. 5b, c Supplementary Fig. 21 Supplementary Fig. 18a, b	TIRF-SIM GI-SIM	1024 × 1024 × 1	ZS-DeconvNet-SIM (×2)	0.23	(1, 90, 1, 100, 3)	29.15	\
Fig. 5d, e	LLS-SIM	F-actin: 625 × 625 × 301	3D ZS-DeconvNet-SIM	56.18	\	\	\
		Mito: 540 × 540 × 151		20.68			

Supplementary Fig. 7a	TIRF	1024 × 1024 × 1	ZS-DeconvNet (×2)	0.23	\	\	\
Supplementary Fig. 7d	TIRF	512 × 512 × 1	ZS-DeconvNet (×2)	0.05	\	\	\
Supplementary Fig. 18<br>Supplementary Video 6	LLSM	512 × 948 × 119	3D ZS-DeconvNet	45.23	\	\	\
Supplementary Fig. 22c	LLS-SIM	640 × 530 × 301	3D ZS-DeconvNet-SIM	50.61	\	\	\
Supplementary Fig. 23	3D-SIM	Lyso: 512 × 512 × 11	ZS-DeconvNet-SIM (×2)	3.51	\	\	\
		MTs: 512 × 512 × 13		3.64			
		Beads: 512 × 512 × 25		5.77			
Supplementary Fig. 24	TIRF-SIM	800 × 800 × 1	ZS-DeconvNet-SIM (×2)	0.14	(1, 7, 1, 100, 2-20)	20.7-21.7	\
Supplementary Fig. 25	TPM	512 × 512 × 40	3D ZS-DeconvNet (×2)	11.76	\	\	\

① ZS-DeconvNet and ZS-DeconvNet-SIM models with an up-sampling layer are labelled by (×2), which double the images size for high lateral sampling rate.

② All three methods compared here were tested on a workstation with an Intel Core i7-11700 processor and an NVIDIA RTX 3090 graphic processing card (GPU). All computations were carried out on the GPU.

③ The system-related parameters used in the sparse deconvolution algorithm were set according to the optical system and image properties for each experiment. The tunable parameters are listed in the order of (spatially up-sampling flag, fidelity, sparsity, sparse iteration, deblur iteration), which were carefully tuned to balance the structural sharpness, noise suppression, and the computational time, following the instructions provided with the corresponding MATLAB software.

Supplementary Table 3. Imaging conditions of live-cell experiments

	Imaging method	Sample	Label	Excitation NA	Excitation λ (nm)	Exposure time per raw image (ms)	Total acquisition time	Illumination intensity	Cycle time (Acquisition + resting time) (sec)	Time points
Figs. 2a, b<br>Supplementary Video 2	TIRF microscopy	COS-7	lifeact-mEmerald<br>myosion2-Halo-JF549	1.41	488, 560	5	0.19s	488: 2 W/cm^2<br>560: 10.8 W/cm^2	5	110
Figs. 2c, d<br>Supplementary Video 3	TIRF microscopy	COS-7	lifeact-mEmerald<br>myosion2-Halo-JF549	1.41	488, 560	5	0.19s	488: 1.7 W/cm^2<br>560: 10.8 W/cm^2	5	825
Fig. 2e, f, i<br>Supplementary Video 4	TIRF microscopy	SUM-159	EGFP-Rab11<br>Lamp1-Halo-JF549	1.35	488, 560	1	0.086s	488: 119-412 W/cm^2<br>560: 57.1-149 W/cm^2	0.33	1499
Fig. 3e, f<br>Supplementary Video 5	LLSM	HeLa	Calnexin-mEmerald<br>H2B-Halo-JF642<br>Mito-dsRed	0.25, 0.14	488, 560, 642	10	5.904s	488: 1.39 μW<br>560: 0.63 μW<br>642: 0.14 μW	10	937
Fig. 4e-h<br>Supplementary Video 8	3D WF microscopy	*C. elegans* embryo	wyEx51119, jcIs1<br>qxIs257	1.35	488, 560	10	8.705s	488: 2.4 mW<br>560: 3.6 mW	30	213
Supplementary Fig. 18<br>Supplementary Video 6	LLSM	HeLa	SC35-mEmerald<br>H2B-mCherry	0.25, 0.14	488, 560	10	3.706s	488: 2.46 μW<br>560: 9.29 μW	30	318

Supplementary Table 4. Descriptions and suggested values of hyper-parameters in ZS-DeconvNet

Hyper-parameter		Description	Suggested value
Parameters defined in loss function	PSF	PSF of the optical system used for calculating deconvolution loss	Experimentally acquired PSF
	μ	Scalar weight to balance denoising loss and deconvolution loss	0.5 for all
	λ	Scalar weight to balance the Hessian regularization	0.02 for 2D models; 0.1 for 3D models
	γ	Scalar weight to balance the gap amending regularization	1 for all
Parameters defined in recorruption	α	Noise magnification factor	[1, 2] for all
	β_1	Poissonian factor	[0.5, 1.5] for all
	β_2	Gaussian factor	Estimated from input images or blank frames

Supplementary References

1. Pang, T., Zheng, H., Quan, Y. & Ji, H. in Proceedings of the IEEE/CVF Conference on Computer Vision and Pattern Recognition 2043-2052 (2021).
2. Qiao, C. et al. Rationalized deep learning super-resolution microscopy for sustained live imaging of rapid subcellular processes. *Nature Biotechnology* (2022).
3. Gustafsson, M.G. et al. Three-dimensional resolution doubling in wide-field fluorescence microscopy by structured illumination. *Biophys J* **94**, 4957-4970 (2008).
4. Liu, S. et al. sCMOS noise-correction algorithm for microscopy images. *Nature Methods* **14**, 760-761 (2017).
5. Wu, Y. & Shroff, H. Faster, sharper, and deeper: structured illumination microscopy for biological imaging. *Nature Methods* **15**, 1011-1019 (2018).
6. Qiao, C. et al. Evaluation and development of deep neural networks for image super-resolution in optical microscopy. *Nature Methods* **18**, 194-202 (2021).
7. Lehtinen, J. et al. Noise2noise: Learning image restoration without clean data. *arXiv preprint arXiv:1803.04189* (2018).
8. Schindelin, J. et al. Fiji: an open-source platform for biological-image analysis. *Nature Methods* **9**, 676-682 (2012).
9. Weigert, M. et al. Content-aware image restoration: pushing the limits of fluorescence microscopy. *Nature Methods* **15**, 1090-1097 (2018).
10. Noise2Void - Learning Denoising from Single Noisy Images. *CVPR* (2019).
11. Ouyang, W. et al. BioImage Model Zoo: A Community-Driven Resource for Accessible Deep Learning in BioImage Analysis. *bioRxiv* (2022).
12. Li, Y. et al. Incorporating the image formation process into deep learning improves network performance. *Nature Methods* (2022).
13. Huang, X. et al. Fast, long-term, super-resolution imaging with Hessian structured illumination microscopy. *Nature Biotechnology* **36**, 451-459 (2018).
14. Guo, Y. et al. Visualizing Intracellular Organelle and Cytoskeletal Interactions at Nanoscale Resolution on Millisecond Timescales. *Cell* **175**, 1430-1442 e1417 (2018).
15. Li, X. et al. Spatial redundancy transformer for self-supervised fluorescence image denoising. *Nature Computational Science* **3**, 1067-1080 (2023).
16. Zhang, G. et al. Bio-friendly long-term subcellular dynamic recording by self-supervised image enhancement microscopy. *Nature Methods* **20**, 1957-1970 (2023).
17. Ning, K. et al. Deep self-learning enables fast, high-fidelity isotropic resolution restoration for volumetric fluorescence microscopy. *Light: Science & Applications* **12**, 204 (2023).
18. Li, X. et al. Three-dimensional structured illumination microscopy with enhanced axial resolution. *Nature Biotechnology* (2023).
19. Wang, Z. et al. High-speed image reconstruction for optically sectioned, super-resolution structured illumination microscopy. *Advanced Photonics* **4**, 026003 (2022).
20. Wen, G. et al. High-fidelity structured illumination microscopy by point-spread-function engineering. *Light: Science & Applications* **10**, 1-12 (2021).
21. Zhao, W. et al. Sparse deconvolution improves the resolution of live-cell super-resolution fluorescence microscopy. *Nature Biotechnology* **40**, 606-617 (2021).

22. Prakash, M., Delbracio, M., Milanfar, P. & Jug, F. in International Conference on Learning Representations (2021).
23. Krull, A., Buchholz, T.-O. & Jug, F. in Proceedings of the IEEE/CVF conference on computer vision and pattern recognition 2129-2137 (2019).
24. Huang, T., Li, S., Jia, X., Lu, H. & Liu, J. in Proceedings of the IEEE/CVF Conference on Computer Vision and Pattern Recognition 14781-14790 (2021).
25. Culley, S. et al. Quantitative mapping and minimization of super-resolution optical imaging artifacts. *Nat Methods* **15**, 263-266 (2018).
