# Supplementary material for: Zero-shot learning enables instant denoising and super-resolution in optical fluorescence microscopy
Source: Nat Commun. 2024 May 16;15:4180. doi: 10.1038/s41467-024-48575-9 (PMC11099110; doi:10.1038/s41467-024-48575-9)
Supplement: Supplementary file 3 — Description of Additional Supplementary Files [file 41467_2024_48575_MOESM3_ESM.pdf]

Description of Additional Supplementary Files

Supplementary Movie 1 | Comparison of ZS-DeconvNet and DeepCAD-based deconvolution networks trained with time-lapse data of different framerates. Top row: simulated time-lapse images of moving tubular structures at framerates of 10Hz, 3Hz, and 1Hz, respectively. Middle and bottom rows: Restored images via DeepCAD-based deconvolution models (middle row) and ZS-DeconvNet (bottom row) trained with time-lapse data at corresponding framerates.

Supplementary Movie 2 | Remodeling dynamics of F-actin and Myosin IIA during adhesion process after dropping a U2OS cell onto the coverslip. ZS-DeconvNet enables time-lapse records of the adhesion and spreading dynamics of a U2OS cell co-expressing mEmerald-lifeact (cyan) and mCherry-myosin IIA (yellow) for 110 time points at ~150 nm resolution and 5 sec intervals.

Supplementary Movie 3 | Remodeling dynamics of F-actin and Myosin II in a second U2OS cell after dropped onto the coverslip. ZS-DeconvNet enhanced TIRF imaging of 825 time points at 5 sec intervals in another U2OS cell co-expressing mEmerald-lifeact (cyan) and mCherry-myosin-IIA (yellow), recording the crawling behavior of the adhesion cell around the contact site to explore the neighborhood before spreading.

Supplementary Movie 4 | Dynamics of recycling-endosomes (REs) and lysosomes or late endosomes (Lyso/LEs) revealed by ZS-DeconvNet. The rapid dynamics of REs and Lyso/LEs are captured at the spatiotemporal resolution of 150 nm and 3 Hz for ~1,500 time points in a gene-edited SUM159 cell endogenously expressing EGFP-Rab11 (Green) and mCherry-Lamp1 (magenta). Right panels present the magnified images of the boxed region in the left, showing the dynamic behaviors of Lyso/LEs (top) and REs (bottom) of interests and their motion trajectories.

Supplementary Movie 5 | Subcellular dynamics and interactions of mitochondria and ER during mitosis visualized via 3D ZS-DeconvNet. SNR and resolution enhanced three-color volumetric imaging via 3D ZS-DeconvNet for 937 time points at 10 sec intervals in a HeLa cell

stably expressing calnexin-mEmerald (ER in grey), H2B-mCherry (chromosome in orange) and Mito-Halo (mitochondria in cyan).

Supplementary Movie 6 | Visualizing disassemble and reassemble process of nuclear speckles during mitosis as seen by 3D ZS-DeconvNet enhanced LLSM. Two-color volumetric super-resolution imaging of nuclear speckles (green) and chromosomes (magenta) labelled with mEmerald-SC35 and H2B-mCherry, respectively, at the speed of 30 sec per volume over 318 time points within a FOV of a group of HeLa cells, recording the entire multi-phase separation dynamics of nuclear speckles during mitosis with a high spatiotemporal resolution.

Supplementary Movie 7 | Comparison of different deconvolution methods on a four-color confocal stack of an early mouse embryo. 3D rendering of an early mouse embryo immunostained for microtubule bridges (cyan, left panel), chromosomes (orange, left panel), actin rings (magenta, right panel), and apical domain (green, panel), showing the contrast and resolution comparison across the mouse embryo, from raw confocal microscopy, RL deconvolution, sparse deconvolution, and 3D ZS-DeconvNet.

Supplementary Movie 8 | Volumetric dynamics of lysosomes and hypodermal cell fusion during the development of a *C. elegans* embryo revealed by 3D ZS-DeconvNet. Long-term volumetric SR imaging of a *C. elegans* embryo via 3D ZS-DeconvNet enhanced wide-filed microscopy for over 213 time points at 30 sec intervals with negligible photobleaching and phototoxicity.

Supplementary Movie 9 | Demo of training a new ZS-DeconvNet model on opened images with the Fiji plugin. Screen captures of the training and inference procedures of the ZS-DeconvNet Fiji plugin on two different low SNR dataset (images of lysosomes and endoplasmic reticulum) with two types of operating systems (Windows 10 and Ubuntu 18.04.6 LTS).
