# Supplementary material for: Zero-shot learning enables instant denoising and super-resolution in optical fluorescence microscopy
Source: Nat Commun. 2024 May 16;15:4180. doi: 10.1038/s41467-024-48575-9 (PMC11099110; doi:10.1038/s41467-024-48575-9)
Supplement: Supplementary file 13 — Reporting Summary [file 41467_2024_48575_MOESM13_ESM.pdf]

nature portfolio

Corresponding author(s): Qionghai Dai, Dong Li

Last updated by author(s): May 2, 2024

Reporting Summary

Nature Portfolio wishes to improve the reproducibility of the work that we publish. This form provides structure for consistency and transparency in reporting. For further information on Nature Portfolio policies, see our Editorial Policies and the Editorial Policy Checklist.

Statistics

For all statistical analyses, confirm that the following items are present in the figure legend, table legend, main text, or Methods section.

n/a	Confirmed	
☐	☒	The exact sample size (n) for each experimental group/condition, given as a discrete number and unit of measurement
☐	☒	A statement on whether measurements were taken from distinct samples or whether the same sample was measured repeatedly
☐	☒	The statistical test(s) used AND whether they are one- or two-sided<br>*Only common tests should be described solely by name; describe more complex techniques in the Methods section.*
☒	☐	A description of all covariates tested
☐	☒	A description of any assumptions or corrections, such as tests of normality and adjustment for multiple comparisons
☐	☒	A full description of the statistical parameters including central tendency (e.g. means) or other basic estimates (e.g. regression coefficient) AND variation (e.g. standard deviation) or associated estimates of uncertainty (e.g. confidence intervals)
☐	☒	For null hypothesis testing, the test statistic (e.g. F, t, r) with confidence intervals, effect sizes, degrees of freedom and P value noted<br>*Give P values as exact values whenever suitable.*
☒	☐	For Bayesian analysis, information on the choice of priors and Markov chain Monte Carlo settings
☐	☒	For hierarchical and complex designs, identification of the appropriate level for tests and full reporting of outcomes
☒	☐	Estimates of effect sizes (e.g. Cohen's d, Pearson's r), indicating how they were calculated

Our web collection on statistics for biologists contains articles on many of the points above.

Software and code

Policy information about availability of computer code

Data collection	The 2D-SIM data of CCPs and MTs used for evaluating ZS-DeconvNet is from the publicly accessible dataset BioSR (https://doi.org/10.6084/m9.figshare.13264793). All other data were collected using our home-built systems and home-written control softwares in which the details can be found in the Methods section. The methods and conclusion reported in this work is not specific to the control program, but can be generalized to other optical imaging systems.
Data analysis	The statistical evaluation of proposed methods and analysis of biological data were performed with Matlab 2017b and Fiji.<br>The deep learning models were trained, validated and tested using python3.9.7 and Tensorflow2.5.0. The python codes of ZS-DeconvNet, the Fiji plugin, several representative pre-trained models, as well as some example data for training and testing are already publicly accessible on the tutorial homepage (https://tristazeng.github.io/ZS-DeconvNet-page/) of ZS-DeconvNet and Github repository (https://github.com/TristaZeng/ZS-DeconvNet).

For manuscripts utilizing custom algorithms or software that are central to the research but not yet described in published literature, software must be made available to editors and reviewers. We strongly encourage code deposition in a community repository (e.g. GitHub). See the Nature Portfolio guidelines for submitting code & software for further information.

Data

Policy information about availability of data

All manuscripts must include a data availability statement. This statement should provide the following information, where applicable:

- Accession codes, unique identifiers, or web links for publicly available datasets
- A description of any restrictions on data availability
- For clinical datasets or third party data, please ensure that the statement adheres to our policy

The SIM data of CCPs and MTs used for evaluating ZS-DeconvNet is from the publicly accessible dataset BioSR (https://doi.org/10.6084/m9.figshare.13264793). Other data that are generated and presented in Figs. 1-5, Supplementary Figs. 1-34, and Supplementary Videos 1-9 in this study are available upon requests. Source data are provided with this paper.

Research involving human participants, their data, or biological material

Policy information about studies with human participants or human data. See also policy information about sex, gender (identity/presentation), and sexual orientation and race, ethnicity and racism.

Reporting on sex and gender	Sex and gender were not considered in study design and no finds were sex- or gender-dependent.
Reporting on race, ethnicity, or other socially relevant groupings	No socially constructed or socially relevant categorization variable(s) were used.
Population characteristics	No human research participants.
Recruitment	No human research participants.
Ethics oversight	No human research participants.

Note that full information on the approval of the study protocol must also be provided in the manuscript.

Field-specific reporting

Please select the one below that is the best fit for your research. If you are not sure, read the appropriate sections before making your selection.

[x] Life sciences [ ] Behavioural & social sciences [ ] Ecological, evolutionary & environmental sciences

For a reference copy of the document with all sections, see nature.com/documents/nr-reporting-summary-flat.pdf

Life sciences study design

All studies must disclose on these points even when the disclosure is negative.

Sample size	Sample size was not predetermined based on statistical calculations. But for every experiment in this study, we generally performed 30~120 replications to ensure reproducibility. The sample size (n) of each experiment is provided in the figure captions in the main text and Supplementary Materials.
Data exclusions	No data was excluded from the analysis.
Replication	The number of repetitions for each experiment is provided in the 'Statistics and Reproducibility' section and the figure legends of the main manuscript and supplementary information files and all repetitions showed similar characteristics and performance.
Randomization	Samples were randomly assigned by independent persons.
Blinding	Data acquisition and analysis were being blinded to the experimental groups.

Reporting for specific materials, systems and methods

We require information from authors about some types of materials, experimental systems and methods used in many studies. Here, indicate whether each material, system or method listed is relevant to your study. If you are not sure if a list item applies to your research, read the appropriate section before selecting a response.

Materials & experimental systems

n/a	Involved in the study	
x		Antibodies
	x	Eukaryotic cell lines
x		Palaeontology and archaeology
	x	Animals and other organisms
x		Clinical data
x		Dual use research of concern
x		Plants

Methods

n/a	Involved in the study	
x		ChIP-seq
x		Flow cytometry
x		MRI-based neuroimaging

Eukaryotic cell lines

Policy information about cell lines and Sex and Gender in Research

Cell line source(s)	COS-7, 293T, U2OS and HeLa cell lines were originally form ATCC. COS-7, 293T, U2OS and HeLa cell lines are gifts from Dr. Junjie Hu's lab in Institute of Biophysics. SUM 159 cell line is the gift from Dr. Tom Kirchhausen's lab in Harvard Medical School, Boston Children's Hospital. C. elegans strains were the gift from Dr. Xiaochen Wang in Caenorhabditis Genetics Center.
Authentication	These cell lines have been authenticated by the vendor.
Mycoplasma contamination	The cell lines used in this study are not contaminated by mycoplasma.
Commonly misidentified lines (See ICLAC register)	There is no misdentified cell line used in this study.

Animals and other research organisms

Policy information about studies involving animals; ARRIVE guidelines recommended for reporting animal research, and Sex and Gender in Research

Laboratory animals	Mice of C57BL/6J background were used in this study. Pre-implantation embryos were isolated from 5-6-week-old females, superovulated by intraperitoneal injection of 5 international units (IU) of pregnant mares' serum gonadotropin (PMSG; LEE BIOSOLUTIONS) and 5 IU human chorionic gonadotropin (hCG; Millipore) 48 h later, and mated with male mice. Zygotes were recovered at E0.5 in M2 medium (Millipore) and cultured in KSOM medium (Millipore) in CO2 incubator (Thermo Scientific) at 37℃ with 5% CO2 until the late 8-cell stage.
Wild animals	We did not use any wild animals.
Reporting on sex	Sex was not considered in study design and no data disaggregated for sex was collected.
Field-collected samples	We did not use any field-collected samples.
Ethics oversight	Experiments involving mouse tissue were performed in accordance with protocols approved by the Institutional Animal Care and Use Committee of CAS center for excellence in molecular cell science, Institute of Biochemistry and Cell Biology, Chinese Academy of Science.

Note that full information on the approval of the study protocol must also be provided in the manuscript.

Plants

Seed stocks	No plants were used in this study.
Novel plant genotypes	No plants were used in this study.
Authentication	No plants were used in this study.
